# The influences of carbon donor ligands on biomimetic multi-iron complexes for N_2_ reduction†

**DOI:** 10.1039/d0sc03447a

**Published:** 2020-08-06

**Authors:** Alexandra L. Nagelski, Majed S. Fataftah, Melissa M. Bollmeyer, Sean F. McWilliams, Samantha N. MacMillan, Brandon Q. Mercado, Kyle M. Lancaster, Patrick L. Holland

**Affiliations:** Department of Chemistry, Yale University 225 Prospect Street New Haven Connecticut 06520 USA patrick.holland@yale.edu; Department of Chemistry and Chemical Biology, Baker Laboratory, Cornell University Ithaca New York 14853 USA kml236@cornell.edu

## Abstract

The active site clusters of nitrogenase enzymes possess the only examples of carbides in biology. These are the only biological FeS clusters that are capable of reducing N_2_ to NH_4_^+^, implicating the central carbon and its interaction with Fe as important in the mechanism of N_2_ reduction. This biological question motivates study of the influence of carbon donors on the electronic structure and reactivity of unsaturated, high-spin iron centers. Here, we present functional and structural models that test the impacts of carbon donors and sulfide donors in simpler iron compounds. We report the first example of a diiron complex that is bridged by an alkylidene and a sulfide, which serves as a high-fidelity structural and spectroscopic model of a two-iron portion of the active-site cluster (FeMoco) in the resting state of Mo-nitrogenase. The model complexes have antiferromagnetically coupled pairs of high-spin iron centers, and sulfur K-edge X-ray absorption spectroscopy shows comparable covalency of the sulfide for C and S bridged species. The sulfur-bridged compound does not interact with N_2_ even upon reduction, but upon removal of the sulfide it becomes capable of reducing N_2_ to NH_4_^+^ with the addition of protons and electrons. This provides synthetic support for sulfide extrusion in the activation of nitrogenase cofactors.

## Abstract 2

High-spin diiron alkylidenes give insight into the electronic structure and functional relevance of carbon in the FeMoco active site of nitrogenase.

## Graphical abstract

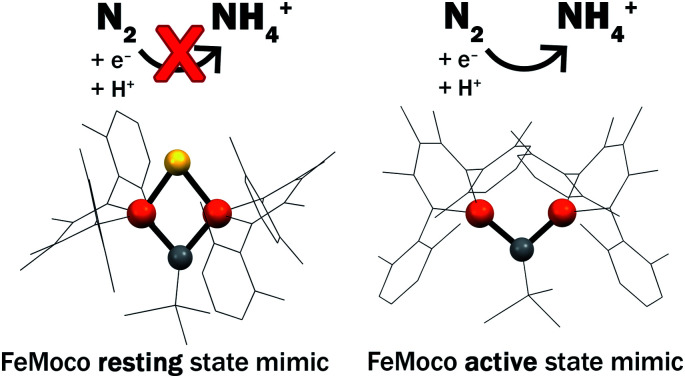

## Introduction

**Fig. 1:**
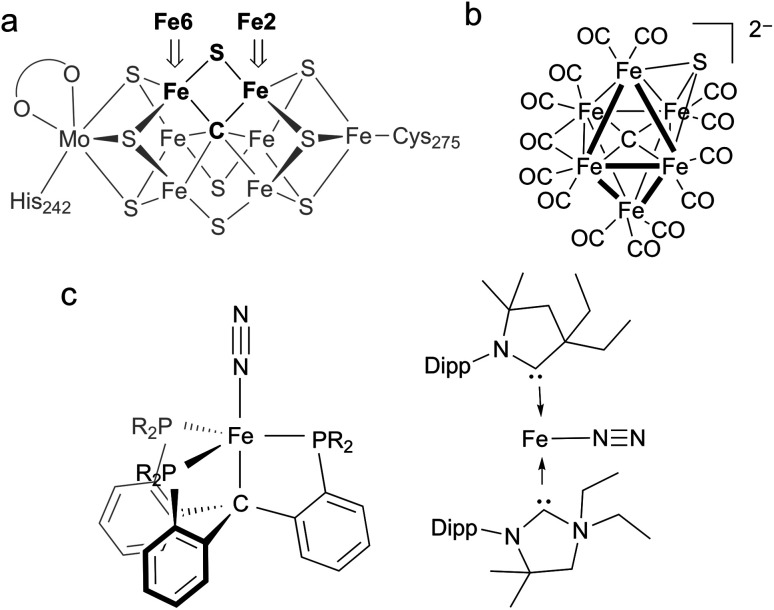
(a) Structure of the resting state of FeMoco with Fe2 and Fe6 labeled for clarity. (b) First example of a synthetic iron carbide sulfide complex. (c) Synthetic iron complexes featuring C-donors capable of N_2_ reduction to NH_4_^+^; the CP^iPr^_3_ complex on the left can be iron(0), iron(i), or iron(ii), while the CAAC complex on the right is iron(0).

Nitrogenases are enzymes that accomplish the impressive feat of reducing N_2_ to NH_4_^+^ at ambient temperatures and pressures. The active site of the most thoroughly studied nitrogenase is the iron–molybdenum cofactor (FeMoco), a unique iron–sulfur cluster composed of one molybdenum and seven iron atoms held together with a number of bridging atoms (Fig. 1a).^1^ A range of kinetic, mutagenesis, and spectroscopic studies support N_2_ binding at the iron atoms of FeMoco,^2–8^ but the structures of intermediate species in the mechanism of N_2_ reduction remain unclear.^1^

A distinguishing feature of the FeMoco is the central carbide (formally C^4−^), which is bound to six iron atoms in the resting state.^9,10^ Isotopic labeling studies show that the carbide is not exchanged during turnover.^11^ X-ray emission spectroscopy has shown that the iron-carbide bonds are highly covalent.^12^ The active-site clusters of nitrogenases are the only known examples of carbides in biological systems, and they are also the only catalysts for N_2_ reduction in nature, implying the carbide serves an essential role in this transformation. However, the specific role of the carbide during the catalytic cycle for N_2_ reduction remains obscure. One hypothesis for N_2_ binding to FeMoco proposes cleavage of hemilabile Fe–S bonds during catalysis, in which case the carbide may function to preserve structural integrity by anchoring the core structure of the cofactor.^7,8,13,14^ This idea is supported by several crystallographic studies showing that the belt sulfide S2B (which bridges Fe2 and Fe6) can be reversibly displaced from the cluster (Fig. 1a).^7,8,14,15^ These results suggest the Fe2/Fe6 locus as a primary substrate binding site in the cofactor. Another hypothesis proposes Fe–C bond cleavage during turnover to create an open coordination site on iron.^3,16^ This could be accompanied by C–H bond formation, an idea that is supported by recent work on a synthetic diiron complex containing a bridging carbyne.^17^ Other proposals involve direct interactions between the carbide and N_2_.^18–20^ The wide variety of these proposals underscores the limited understanding of the structural and electronic contributions of bridging carbon ligands to reactivity in iron–sulfur clusters.

Synthetic complexes offer useful insights as structural or functional models of nitrogenase, but accessing species with both carbon and sulfur donors has been challenging.^21^ In a recent study, Rauchfuss and coworkers reported the first synthetic example of an iron cluster with both carbide and sulfide (Fig. 1b), which have different bridging modes than FeMoco.^22^ In this compound, the CO ligands lead to low-spin iron centers that contrast with the high-spin iron centers found in FeMoco.

In functional synthetic models, mononuclear iron complexes have been used to gauge the N_2_-coordinating ability of compounds with Fe–C bonds.^16,17,23–28^ Of these complexes, only two systems produce NH_4_^+^ (3.3–4.6 equiv. NH_4_^+^/Fe) upon treatment with acid and reductant (Fig. 1c).^16,27^ A low-spin iron system, Fe(CP^iPr^_3_)N_2_, where CP^iPr^_3_ is tris(2-(diisopropylphosphino)phenyl)methyl, displays lengthening of Fe–C bonds during reduction. The other, (CAAC)_2_Fe (CAAC = cyclic (alkyl)(amino)carbene), is capable of mediating N_2_ reduction to NH_4_^+^.^16,27,29^ Studies of iron species with bridging carbon and sulfur ligands that are high-spin with greater electronic and structural fidelity to the Fe2/Fe6 site are needed to improve the understanding of how biological S- and C-based donors impact N_2_ reactivity.

Here, we present a new diiron complex that has both carbon and sulfur bridges between two high-spin iron centers, creating an Fe_2_CS diamond core that structurally overlays with a part of the core in FeMoco.^30–32^ Further, we systematically evaluate the electronic structure and N_2_ reducing ability of three related high-spin iron complexes with the carbon-based donors alkyl and alkylidene. Importantly, the high-spin iron alkyl and alkylidene complexes produce NH_4_^+^ from N_2_.

## Results

### Synthesis

**Scheme 1:**
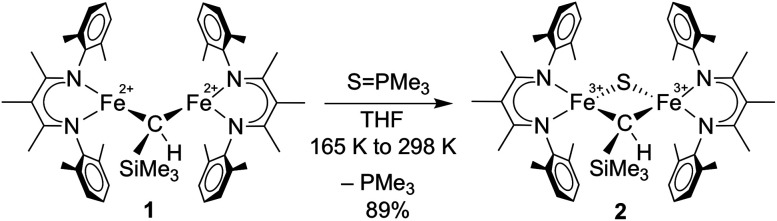
Conversion of [L^Me^Fe]_2_(μ-CHSiMe_3_) (**1**) to [L^Me^Fe]_2_(μ-S)(μ-CHSiMe_3_) (**2**).

**Fig. 2:**
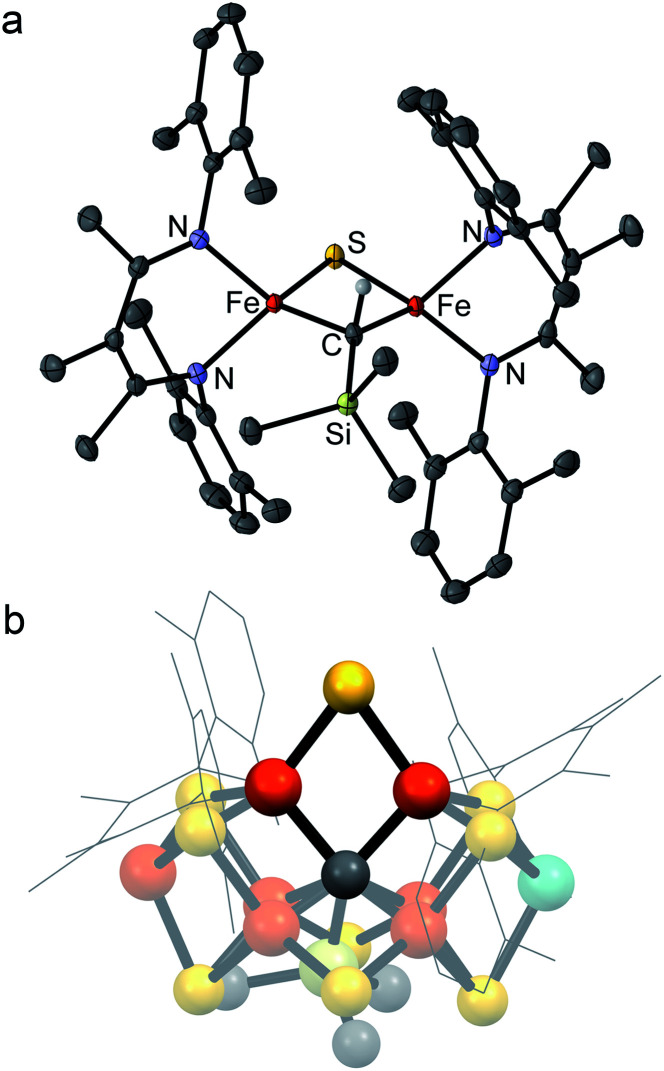
(a) Crystal structure of [L^Me^Fe]_2_(μ-S)(μ-CHSiMe_3_) (**2**) with thermal ellipsoids shown at 50% probability. Color scheme: iron in orange, nitrogen in blue, carbon in gray, silicon in green, and sulfur in yellow. Hydrogen atoms have been omitted for clarity except on the alkylidene ligand. (b) Structural overlay of **2** with Fe2/Fe6/S2B/C rhomb in the FeMoco (nitrogenase crystal structure PDB 3U7Q).

We previously reported the first high-spin iron complex with an unsupported alkylidene bridge, [L^Me^Fe]_2_(μ-CHSiMe_3_) (**1**, where L^Me^ = 3-methyl-2,4-bis(2,6-xylylimido)pentyl).^33^ Treatment of **1** with 1 equiv. of the sulfur atom transfer reagent Me_3_PS in a thawing THF solution forms one major species, [L^Me^Fe]_2_(μ-S)(μ-CHSiMe_3_) (**2**), which was isolated in 89% yield (Scheme 1). This material is 95% pure as judged by Mössbauer spectroscopy, and was sufficient for extensive characterization. Extracting the crude reaction into Et_2_O and cooling to 233 K yielded green crystals. The molecular structure of **2** (Fig. 2a) has one sulfide bridge and one CHSiMe_3_ bridge between two identically-bonded iron(iii) centers. The ^1^H NMR spectrum is consistent with each diketiminate environment having a single mirror plane perpendicular to the FeN_2_C_3_ plane, which agrees with the core symmetry but is surprising given the overall *C*_s_ symmetry expected for the molecule. While this may indicate dynamic cleavage of the Fe–C bonds, we have no other evidence for this behavior. It is also notable that the resonances in the ^1^H NMR spectrum are shifted and broadened, indicating population of paramagnetic states that is explored below through magnetic susceptibility measurements.

Several structural comparisons are striking. The average Fe–C bond length in the diiron(iii) complex **2** is 1.994(2) Å, which is 0.030(8) Å longer than the average Fe–C bond in the diiron(ii) complex **1** despite the higher oxidation state of iron. The sulfide bridge causes the Fe–C–Fe bond angle to decrease from 95.6(3)° in **1** to 81.74(6)° in **2**. The diamond core in **2** is contracted relative to the one in a previously reported bis(sulfide) diiron(iii) complex [L^Me^FeS]_2_, as in **2** the Fe–S bonds are 0.116(2) Å shorter and the Fe⋯Fe distance is 0.641(2) Å shorter.^34,35^ Importantly, the core of **2** overlays well with the rhomb containing Fe2 and Fe6 in FeMoco, as the Fe–C and Fe–S average bond lengths in both structures are similar (Fe–C_avg_ is 1.994(2) Å in **2***vs.* 2.00 Å in FeMoco; Fe–S_avg_ is 2.217(8) Å in **2** and 2.25 Å in FeMoco for the Fe2/Fe6 rhomb).^10^ The Fe⋯Fe distance in **2** is 2.6027(6) Å, which matches the distances between belt iron atoms in the crystal structure of FeMoco (2.61 Å) quite well. The overall Fe/Fe/S/C core in **2** overlays with the Fe2/Fe6/S2B/C core of the resting state FeMoco with an root-mean-square deviation (RMSD) of 0.08 Å (Fig. 2b). These comparisons indicate that despite the difference in carbon coordination number, the alkylidene in **2** serves as an accurate structural model for the carbide bridge in FeMoco.

**Scheme 2:**
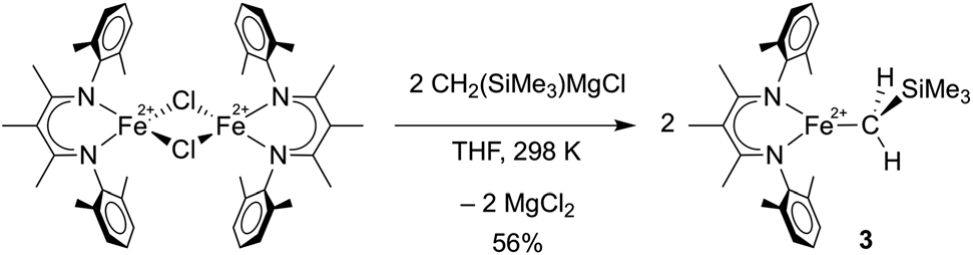
Preparation of L^Me^FeCH_2_SiMe_3_ (**3**).

To compare the influence of the alkylidene in **1** to a mononuclear alkyl analogue, we also prepared a three-coordinate iron(ii) alkyl complex with diketiminate supporting ligands using a known method.^36,37^ Adding 2.1 equiv. of MgBrCH_2_SiMe_3_ to a solution of [L^Me^FeCl]_2_ ^38^ in THF led to the trimethylsilylmethyl iron(ii) complex **3**, which could be isolated in 56% yield (Scheme 2). The X-ray crystallographic data of **3** reveal a planar three-coordinate iron center featuring an Fe–C bond length of 2.017(3) Å, which is comparable to the Fe–C bond lengths in other previously reported three-coordinate β-diketiminate iron(ii) alkyl species.^33,36,37,39,40^ Similar to these related compounds, **3** has a high-spin electronic configuration (*S* = 2), as judged by the Evans method in solution (*μ*_eff_ in C_6_D_6_ at 298 K is 5.4(2) *μ*_B_), and averaged *C*_2v_ symmetry evident in its ^1^H NMR spectrum indicating rapid rotation around the Fe–C bond.

### Reduction of N_2_ to NH_4_^+^ by C-bound iron complexes

**Scheme 3:**
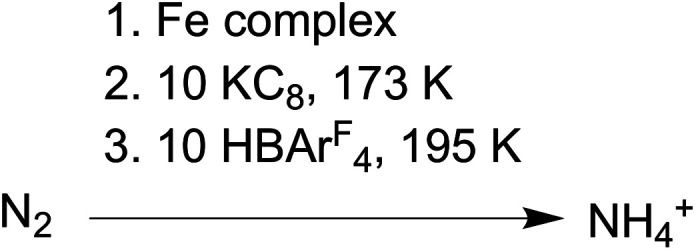
Conditions for N_2_ reduction experiments.

The conversion of N_2_ to NH_4_^+^ with the addition of reductant and acid was used to evaluate compounds **1–3** as functional models of nitrogenase. These studies used KC_8_ and [H(Et_2_O)_2_][BAr^F^_4_] (HBAr^F^_4_) under conditions similar to those in several catalytic systems (see ESI†).^27,41^ In our work, the reductant (10 equiv.) was added first to the complexes at 173 K and then the acid (10 equiv.) was added to the frozen mixture and stirred at 195 K (Scheme 3). This order of addition minimizes the potential for competitive protonation of the β-diketiminate ligand.^42^

Using Et_2_O as solvent, complexes **1** and **3** generate 50–70% yield of NH_4_^+^ per complex while **2** produces only trace amounts (left side of Table 1). Analogous experiments using **1** and **3** performed under an atmosphere of ^15^N_2_ yield exclusively ^15^NH_4_^+^ and under an atmosphere of Ar yield < 5% of NH_4_^+^/complex (Fig. S6, 7 and Table S1†). These control experiments demonstrate the NH_4_^+^ formed by **1** and **3** is derived from N_2_, not from the diketiminate ligands or impurities. We also tested N_2_ reduction in THF (right side of Table 1), but the NH_4_^+^ yields were roughly five times lower than the analogous experiments in Et_2_O. We hypothesize that this difference is a result of less favorable N_2_ binding in THF (*vide infra*).

**Table 1:** Ammonium yields from N_2_ mediated by iron complexes upon the addition of reductant (KC_8_) and acid (HBAr^F^_4_)^a^

	Et_2_O			THF		
	NH_4_^+^ per complex	% Yield per complex	% Yield per Fe	NH_4_^+^ per complex	% Yield per complex	% Yield per Fe
**1**	1.12 ± 0.06	56 ± 3	28 ± 1	0.29	15	7.4
**2**	0.10 ± 0.05	5 ± 3	2 ± 2	—	—	—
**3**	1.37 ± 0.17	68 ± 7	68 ± 7	0.25 ± 0.03	13 ± 2	13 ± 2
**4**	1.10 ± 0.03	55 ± 1	55 ± 1	0.19	9	9

a Ammonium determined using the indophenol method. Error represented as a range of multiple trials; lack of error bar indicates a single trial.

To explore the species responsible for NH_4_^+^ production from **1**, we conducted low temperature ^1^H NMR studies with smaller amounts of reductant and acid. Reduction of **1** with 1.6 equiv. of KC_8_ in THF-*d*_8_ at 203 K showed trace amounts (<10%) of **1** and **3**, but most of the mixture consisted of unidentified species that we were unable to isolate due to thermal decomposition above 203 K. Further attempts to isolate reduced forms of **1** were not successful (see ESI†). As a result, we cannot confidently attribute the N_2_ reduction by **1** to any one active species. However, we reason that since the conversion of **1** to **3** upon reduction is relatively low, the N_2_ reduction activity of **1** cannot be solely attributed to the formation of **3** under these conditions (see ESI† for further discussion). It is therefore evident that there is some reduced form of **1** (or a degradation product therefrom) that is capable of N_2_ binding and reduction to NH_4_^+^.

**Scheme 4:**
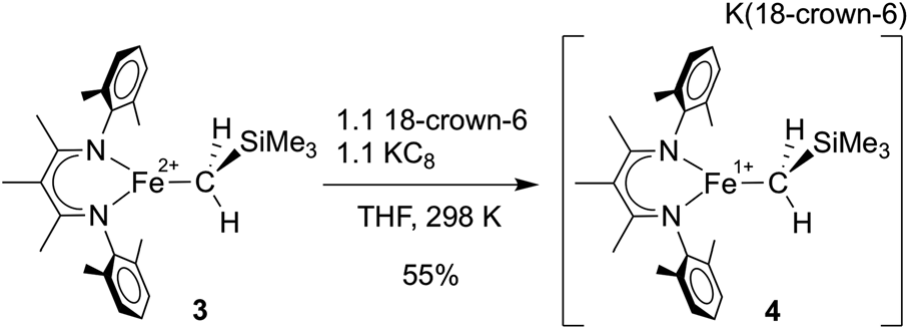
Preparation of [L^Me^FeCH_2_SiMe_3_][K(18-crown-6)] (**4**).

In separate reduction experiment, treatment of **3** with 1.2 equiv. of KC_8_ in the presence of 1.2 equiv. of 18-crown-6 in THF formed the iron(i) complex [L^Me^FeCH_2_SiMe_3_][K(18-crown-6)] (**4**) in 55% yield (Scheme 4). Treatment of **4** with additional reductant did not result in further chemical changes. It was possible to characterize **4** in detail, including an X-ray crystal structure that showed separated cations (in which two THF molecules are bound to K^+^ in addition to the 18-crown-6; see ESI†) and anions (in which the iron(i) ion is three-coordinate). The THF molecules are weakly bound to the K(18-crown-6) cation, as indicated by their absence in a low-quality crystal structure in which the K(18-crown-6)^+^ unit was coordinated to the supporting ligand. Microanalysis also indicated the absence of the THF molecules in the isolated solid.

We investigated the ability of **4** to produce NH_4_^+^ from N_2_ under conditions similar to those described above, and found that it generates NH_4_^+^ in comparable yields to **3** (Table 1). These experiments suggest that **4** is a feasible intermediate during the series of transformations leading to N_2_ reduction by **3**.

### N_2_ binding

**Fig. 3:**
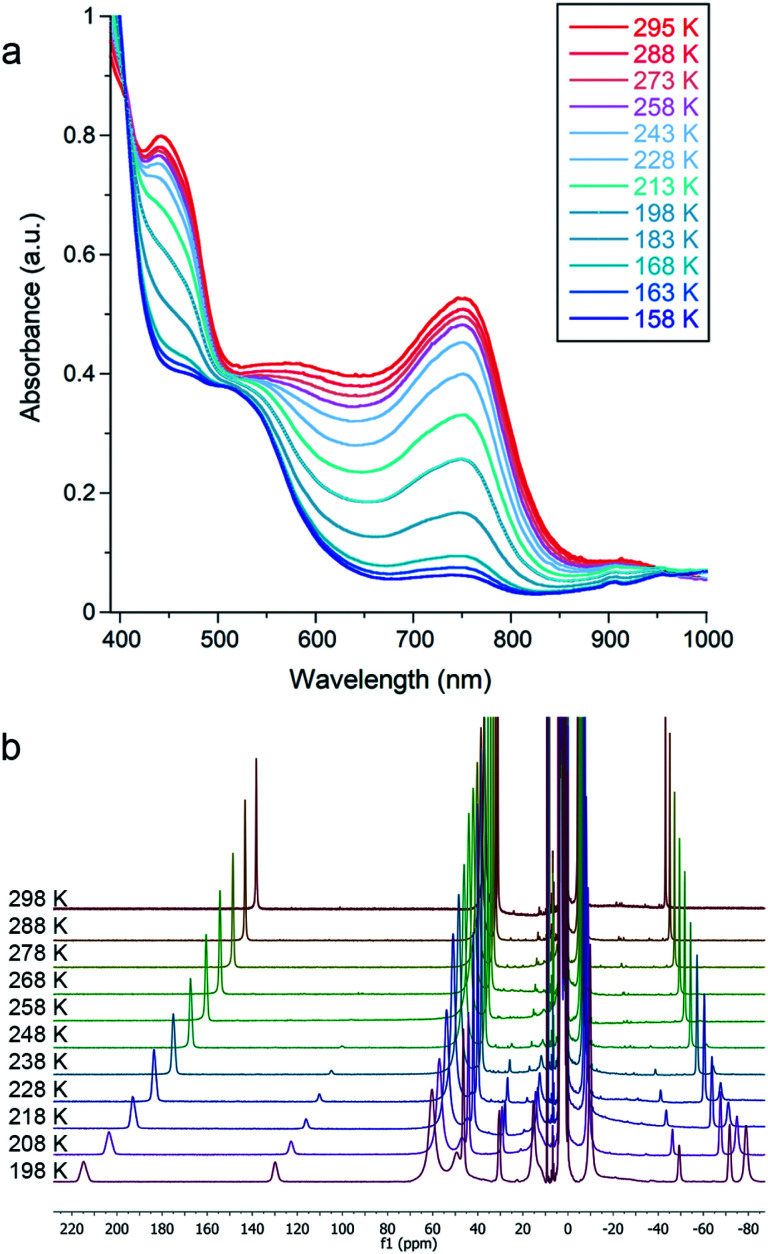
(a) Variable temperature electronic absorption spectra of **4** in Et_2_O under N_2_. Absorbance values were corrected to account for the change in density of the solvent with temperature. (b) Variable temperature ^1^H NMR spectra of **4** in THF-*d*_8_ under N_2_.

Cooling **1**, **2**, or **3** under N_2_ in THF-*d*_8_ yielded no spectroscopic changes (Fig. S8–S10 and S16†). In contrast, cooling an Et_2_O solution of **4** under 1 atm N_2_ resulted in a color change from green to red that was monitored using electronic absorption spectroscopy (Fig. 3a). Under 1 atm N_2_, a room-temperature solution of **4** in Et_2_O displayed prominent absorption bands at 450 and 750 nm whose intensity drastically decreased upon cooling. This marked change did not occur in upon cooling a sample under Ar (Fig. S18†), indicating that the changes come from N_2_ binding. Similar changes in the absorption spectrum were observed when cooling solutions of **4** in THF or 2-methyltetrahydrofuran (MeTHF) under N_2_ (Fig. S19–S21†). At 168 K, the decrease in the intensity of the bands corresponding to **4** was greatest in Et_2_O, followed by THF, then MeTHF (Fig. S17–21†). The higher conversion to **4–N2** in Et_2_O at a given temperature is attributable to the greater solubility of N_2_ in Et_2_O than in THF and MeTHF.^43,44^ Similar mononuclear complexes have demonstrated N_2_ bridging to a κ^1^-bound alkali cation.^45^ Thus, in addition to the differences in N_2_ solubility between these solvents, solvent coordination to the K(18-crown-6) cation could also contribute to the observed differences in N_2_ binding affinity of **4**. Further, the N_2_ reduction experiments in Et_2_O generate approximately four-fold greater NH_4_^+^ yields than analogous experiments run in THF (*vide supra*).

Due to decomposition at concentrations suitable for electronic absorption spectroscopy, we turned to ^1^H NMR spectroscopy for more reproducible quantification. Variable temperature experiments in Et_2_O-*d*_10_ allowed us to quantify the equilibrium between **4** and **4–N2**, which are in slow exchange on the NMR time scale. Van't Hoff plots for N_2_ binding to **4** gave Δ*H*° = −20 ± 1 kJ mol^−1^ and Δ*S*° = −57 ± 3 J mol^−1^ K^−1^, where the large negative entropy is characteristic of binding a gas (Fig. S12†). A parallel experiment performed in THF-*d*_8_ yielded the thermodynamic parameters Δ*H*° = −26 ± 1 kJ mol^−1^ and Δ*S*° = −93 ± 5 J mol^−1^ K^−1^ (Fig. 3b and S14†). These parameters are similar to those for N_2_ binding to the related β-diketiminate iron(i) phenyl complex and other reported iron and cobalt complexes (Table S2†).^27,46,47^

### Spin states and exchange coupling

**Fig. 4:**
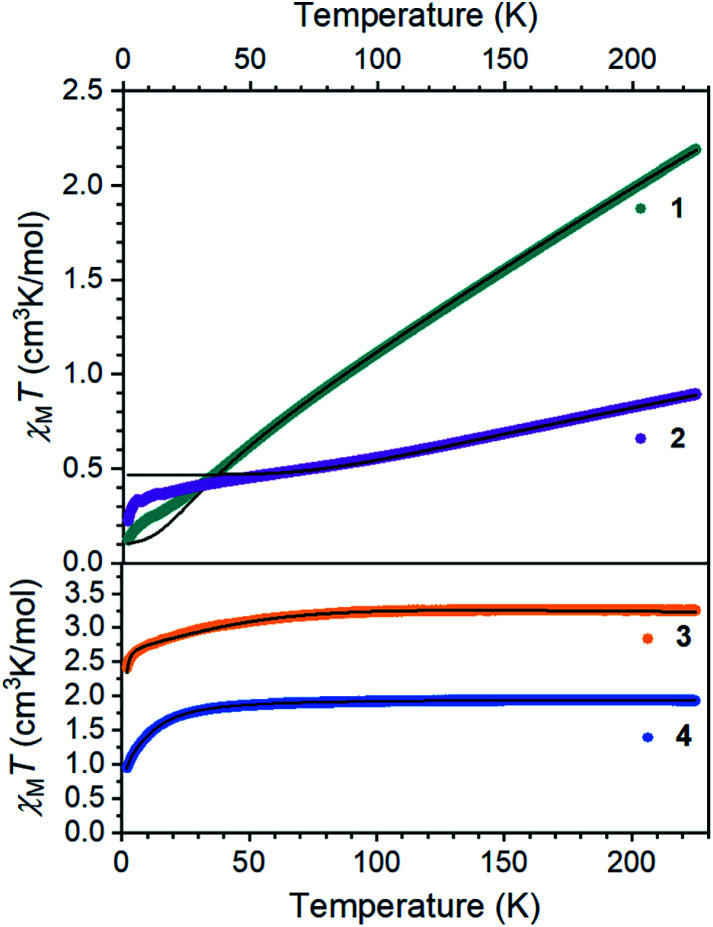
Dc magnetic susceptibility data for **1–4** collected under an applied magnetic field of 1000 Oe. Black lines represent fits to the data.

The electronic structures of **1** and **2** provide valuable insights into the influences of S and C bridges in iron–sulfur clusters. The Mössbauer spectrum^48^ of **1** at 80 K displays signals with an isomer shift of 0.62 mm s^−1^, consistent with high-spin iron(ii) centers.^33^ Inspection of the magnetic susceptibility of **1** reveals a sharp decrease in *χ*_M_*T* with decreasing temperature, reaching a value of 0.12 cm^3^ K mol^−1^ at 2 K (Fig. 4), indicating that the two iron(ii) centers are antiferromagnetically coupled. The value of *χ*_M_*T* depends linearly on T up to a value of 2.17 cm^3^ K mol^−1^ at 225 K, which is consistent with two high-spin iron(ii) centers that are antiferromagnetically coupled. To quantify the magnitude of the antiferromagnetic interaction, the data were fit to the Van Vleck equation according to the spin Hamiltonian: *Ĥ* = *D*(*Ŝ*_1z_^2^+ *Ŝ*_2z_^2^) + *E*[(*Ŝ*_1x_^2^ − *Ŝ*_1y_^2^) + (*Ŝ*_2x_^2^ − *Ŝ*_2y_^2^)] + (*g*_1_ + *g*_2_)μ_B_***SH*** − 2*J*(*Ŝ*_1_·*Ŝ*_2_). In this Hamiltonian, *D* and *E* are the axial and transverse zero-field splitting parameters, *Ŝ*_1_ and *Ŝ*_2_ are the spin operators, *g*_1_ and *g*_2_ are the isotropic *g*-values, and *J* is the magnitude of the exchange interaction. The best fit to the experimental data for **1** was accomplished with an exchange constant of *J* = −34(2) cm^−1^, and was relatively insensitive to the other parameters (see ESI†).

Next, we examined the spin states in the diiron(iii) complex **2**, which has both C and S bridges. Its zero-field Mössbauer spectrum at 80 K displays a doublet with *δ* = 0.26 mm s^−1^ and |Δ*E*_Q_| = 1.95 mm s^−1^ (Fig. S22†). The isomer shift is much lower than that of **1**, consistent with more oxidized iron centers. To support this assignment, we used density-functional (DFT) calculations at the B3LYP/def2-TZVP(Fe,Si,S,N,CHSiMe_3_)/def2-SVP(C,H) level to give geometry optimized structures in all six possible spin states, and these were used to predict Mössbauer spectra for each spin state using the spectroscopic validation we established for β-diketiminate complexes.^49^ A broken-symmetry model with two antiferromagnetically coupled high-spin iron(iii) centers gave the lowest energy structure, reproduced the crystallographic structure with a RMSD of 0.24 Å, and predicted Mössbauer parameters (*δ*_1_ = 0.35 mm s^−1^, |Δ*E*_Q_|_1_ = 1.62 mm s^−1^, *δ*_2_ = 0.36 mm s^−1^, |Δ*E*_Q_|_2_ = 1.67 mm s^−1^) that are within error of the experimental values; the other spin state possibilities gave isomer shift values that deviated from experiment by at least 0.20 mm s^−1^ or gave two distinct doublets (see ESI† for details). Other four-coordinate diiron(iii) sulfide complexes in the literature also have high-spin electronic configurations.^50–53^ The magnetic susceptibility of **2** displays a *χ*_M_*T* value of 0.91 cm^3^ K mol^−1^ at 225 K that drops with decreasing temperature, supporting an antiferromagnetic exchange interaction between the iron(iii) centers in **2** in agreement with the calculations. We modelled the dc susceptibility data with two high-spin iron(iii) sites using the spin Hamiltonian described above with an exchange constant of *J* = −120(10) cm^−1^ and isotropic *g* = 2.0 (see ESI†).

The mononuclear compounds display *χ*_M_*T* values of 3.26 (**3**) and 1.93 (**4**) cm^3^ K mol^−1^ at 225 K. These values are consistent with ground states of *S* = 2 for **3** (high-spin iron(ii)) and *S* = 3/2 for **4** (high-spin iron(i)). The value of *χ*_M_*T* decreases with decreasing temperature due to zero-field splitting. We fit the data with *D* values of −45.7(3) cm^−1^ for **3** and −14.9(2) cm^−1^ for **4**. These large zero-field splitting parameters are consistent with those observed in other three-coordinate iron(ii) complexes.^54–58^ The high-spin assignment is also consistent with the Mössbauer spectra of these mononuclear complexes, which display quadrupole doublets with *δ* = 0.43 mm s^−1^ and |Δ*E*_Q_| = 1.28 mm s^−1^ for **3**, and *δ* = 0.41 mm s^−1^ and |Δ*E*_Q_| = 2.23 mm s^−1^ for **4** (Fig. S23 and S24†). These values are consistent with other high-spin iron(ii) and iron(i) complexes in the literature, and DFT calculations similar to those described above validated these spin state assignments (see ESI†).^55,59–61^

**Fig. 5:**
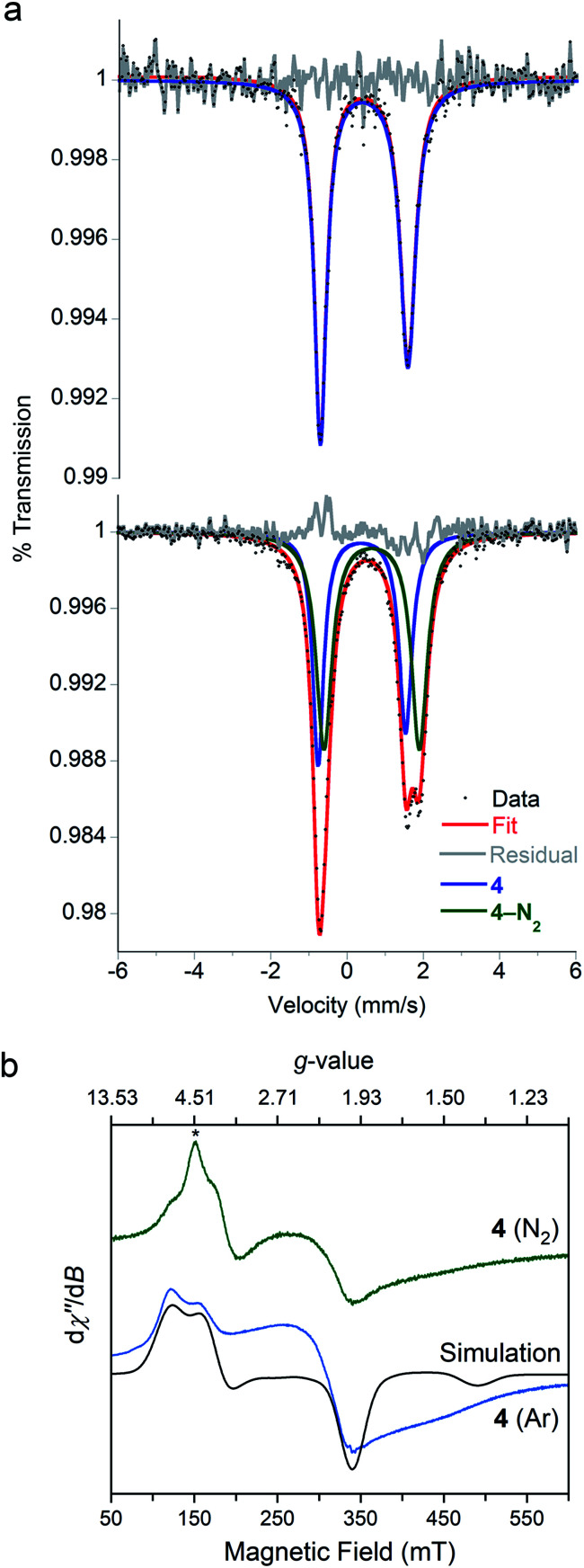
(a) Zero-field ^57^Fe Mössbauer spectra of a frozen solution of **4** in MeTHF at 80 K, frozen under 1 atm of argon (top) or 1 atm of **N2** (bottom). **4**: *δ* = 0.42 mm s^−1^ and |Δ*E*_Q_| = 2.29 mm s^−1^ (48% of N_2_ spectrum); **4–N2**: *δ* = 0.64 mm s^−1^ and |Δ*E*_Q_| = 2.55 mm s^−1^ (52% of N_2_ spectrum). (b) Overlay of the X-band (9.436 GHz) EPR spectra of **4** at 77 K collected under an atmosphere of Ar (blue) and N_2_ (green). The black line is a simulation of **4**–Ar spectrum that is consistent with a *S* = 3/2 ground state. The asterisk highlights the new resonance observed under N_2_.

We were interested in the spin state of **4–N2**, but our inability to isolate it prevented characterization by magnetometry. However, the Mössbauer spectrum of **4** flash frozen in N_2_-saturated MeTHF was collected at 80 K and displays an additional doublet that was not observed in a control experiment under Ar. We attribute this new signal to **4–N2** (Fig. 5a), for which the best fit has *δ* = 0.64 mm s^−1^ and |Δ*E*_Q_| = 2.55 mm s^−1^. The isomer shift is consistent with reported values of high-spin, four-coordinate iron(i) (*S* = 3/2) yet too high for reported values for iron(i) in low spin configurations (*S* = 1/2).^55,59–61^ Geometry optimizations were performed for both possible spin states of iron(i) in **4–N2**, and DFT calculations^49^ were used to predict Mössbauer parameters for each model. The *S* = 3/2 model predicted parameters (*δ* = 0.64 mm s^−1^, |Δ*E*_Q_| = 2.14 mm s^−1^) that are close to the experimental values, while the *S* = 1/2 model (*δ* = 0.36 mm s^−1^, |Δ*E*_Q_| = 1.02 mm s^−1^) deviated substantially from the experimental data.

To corroborate our assignment of spin states for **4** and **4–N2** as high-spin iron(i), we measured the X-band EPR spectrum of **4** frozen in MeTHF under N_2_ and Ar at 77 K (Fig. 5b). The EPR spectrum under Ar displays three broad resonances with *g*_eff_ values of 2.1, 3.8 and 5.6 and are consistent with a *S* = 3/2 ground state. We simulated the EPR spectrum with the spin Hamiltonian employed to model the dc susceptibility data with |*D*| and |*E*| values of 12.9 cm^−1^ and 1.7 cm^−1^, and *g* values of 2.36, 2.33, and 2.05. The EPR spectrum for a sample of **4** flash-frozen under N_2_ displays an additional feature at *g*_eff_ = 5.4 that we attribute to **4–N2**. The large effective *g*-value indicates that **4–N2** has a high-spin configuration (*S* = 3/2); however, due to the spectral convolution between **4** and **4–N2**, we could not adequately model the EPR spectrum to extract the spin Hamiltonian parameters for **4–N2**. The agreement with the DFT computations and the Mössbauer spectroscopy, however, supports this assignment of the iron(i) center as *S* = 3/2 in **4–N2**.

### Covalency of bonds

**Fig. 6:**
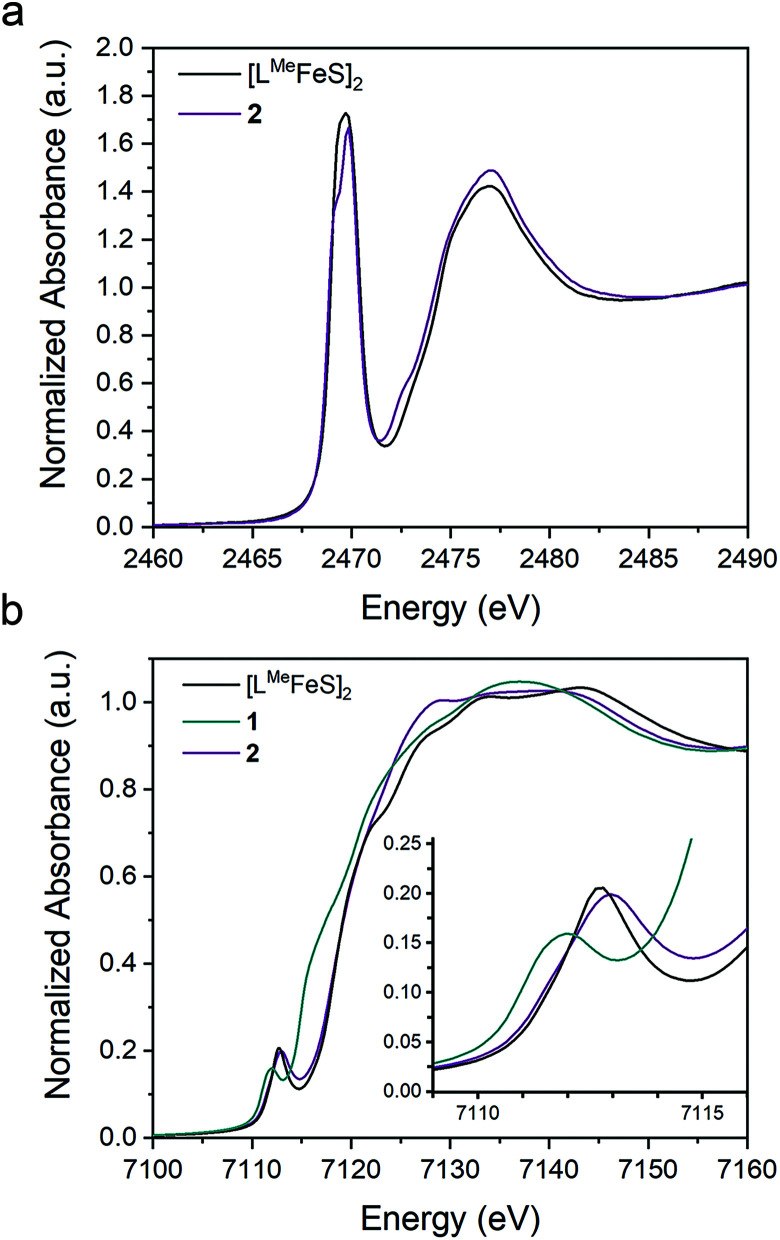
Overlay of the (a) S K-edge XAS spectra of **2** (purple) and [L^Me^FeS]_2_ (black) and (b) Fe K-edge XAS spectra of the **1** (teal), **2** (purple), and [L^Me^FeS]_2_ (black).

To assess the effect of replacing a bridging sulfide with an alkylidene on the Fe–S covalency, we compared sulfur K-edge X-ray absorption spectra (XAS) for **2** to the related bis-sulfide complex, [L^Me^FeS]_2_ (Fig. 6a), which also has two high-spin iron(iii) centers, but these centers are instead bridged by two sulfide ligands.^34^ We examined the pre-edge areas at 2470 eV determined by peak fitting of the S K-edges, in order to quantify the S 3p character in the unoccupied metal d orbitals.^62^ The contribution from the two bridging sulfide ligands in [L^Me^FeS]_2_ is 14% S 3p and the contribution from the single bridging sulfide ligand in **2** is 6%. Because the 3p character in [L^Me^FeS]_2_ is twice the value for **2** and reflects the contributions from two sulfides instead of one, it follows that the Fe–S covalency per bond is not significantly perturbed by the substitution of an alkylidene for one of the sulfide ligands. This interpretation of the XAS agrees with the similar isomer shifts observed for **2** and [L^Me^FeS]_2_, which reflect the electron density at the iron centers (Fig. S17†).

**Fig. 7:**
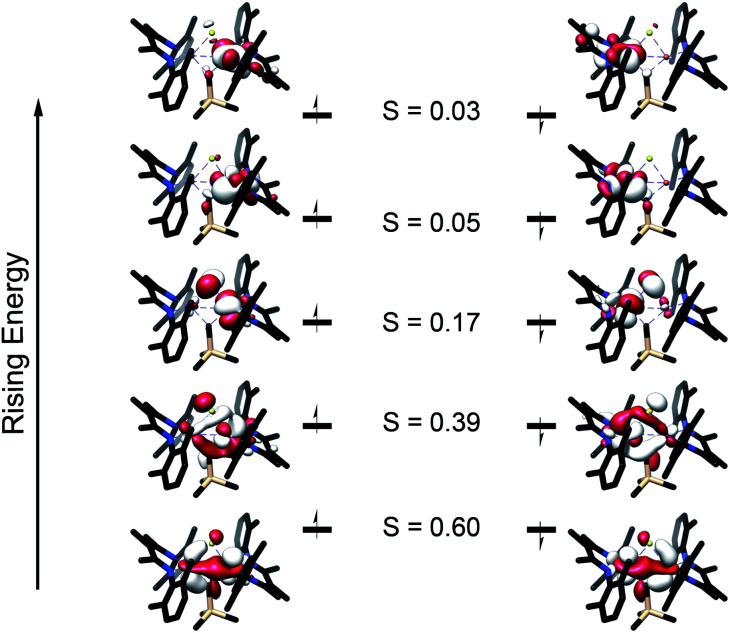
Truncated molecular orbital diagram of **2** generated from the broken symmetry UHF solution. UCOs are shown with the alpha orbitals on the left and the beta orbitals on the right. Orbitals are plotted at an isovalue of 0.03 au. *S* represents the overlap between the alpha and beta orbitals.

Broken symmetry DFT calculations indicated strong antiferromagnetic coupling between iron centers in both [L^Me^FeS]_2_ and **2** (Fig. 7). The overlap (*J*) was −84 cm^−1^ for [L^Me^FeS]_2_ and −313 cm^−1^ for **2**, but these values from a single reference DFT calculation relative to experiment are commonly overestimated.^63–66^ Calculated spectra (Fig. S41–43†) were thus obtained using the lowest energy solution with *S* = 0. It should be noted that the differences in intensities of the calculated S K-edges can be attributed to the difference in number of absorbers. Calculated S 3p character in the unoccupied d-orbitals was 11.94% for [L^Me^FeS]_2_ and 5.94% for **2**. These calculations agree remarkably well with the experimental data described in the previous paragraph, and confirm that the Fe–S covalency is the same for both compounds. These results contrast with those presented in a study by Pollock *et al.*, in which the replacement of a sulfide with an imido (N^*t*^Bu^2−^) in [Fe_2_S_2_Cl_4_]^2−^ decreased the iron–sulfur covalency.^50^

The Fe K-edge XAS obtained for **1**, **2** and [L^Me^FeS]_2_ are presented in Fig. 6b. These data reveal a significant shift in the rising edges between **1** (7115 eV) and **2** (7118 eV), as expected for more oxidized iron sites. The overlaying pre-edge and edge features of **2** and [L^Me^FeS]_2_ in Fe K-edge XAS also reflect similar electronic structures at the iron centers, in agreement with the S K-edge XAS data described above, despite the substitution of the sulfide for an alkylidene.

### Electronic structure

To further understand the nature of the bonding in these complexes, we analyzed the localized orbitals of the broken-symmetry DFT model using the intrinsic atomic orbital-intrinsic bond order (IAOIBO) method.^67^ From this analysis, the Mayer bond order^68^ (MBO) provides a convenient method to sum all of the contributions to the bond; it has been applied to FeMoco and related systems to understand the magnitude of bonding between two atoms.^68,69^ The MBOs for the Fe–C interactions are similar between **3** (0.87) and **1** (0.90). Upon the introduction of a sulfide ligand and oxidation of the iron centers, the Fe–C interactions of the bridging alkylidene in **2** display a similar MBO of 0.86. This MBO analysis demonstrates the similarity of Fe–C bonding across **1**, **2**, and **3**, despite the differences in metal nuclearity, oxidation state, and carbon donor identity.

**Fig. 8:**
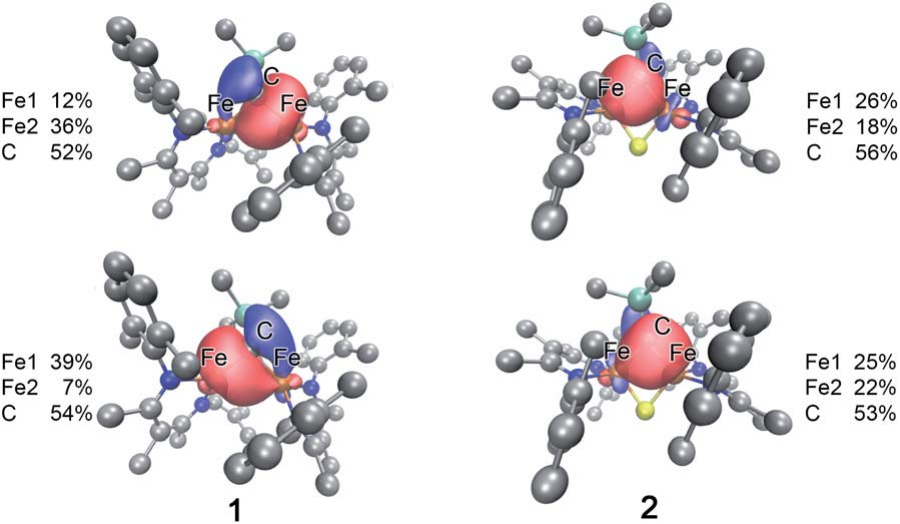
Isosurface plots of select Fe–C interactions in **1** (left) and **2** (right) with the total spin density for Fe and the alkylidene C listed for each plot.

Despite the comparable Fe–C MBO in **1** and **2**, we sought to understand the electronic implications of the Fe–C–Fe angle contraction from 95.6(3)° in **1** to 81.74(6)° in **2**. There is also a substantial distortion of the alkylidene carbon geometry away from tetrahedral moving from **1** (*τ*_4_ = 0.85) to **2** (*τ*_4_ = 0.65, see ESI†).^70^ Analysis of the localized Fe–C orbitals shows that in **1** the electron density in each orbital is more localized on one discrete Fe–C bond, while in **2** there is a delocalized bonding orbital with electron density between both iron centers and the alkylidene carbon (Fig. 8). The IAOIBO analysis of **2** shows the alkylidene exclusively forms σ bonds with the iron centers, while the sulfide forms one σ bond with each iron as well as π-bonding interactions. The covalency of the Fe–S bonds can be measured by summing the Fe–S bonding orbitals in **2**, giving 23% Fe character and 77% S character. In the bis-sulfide complex [L^Me^FeS]_2_ these are similar (24% Fe, 76% S), consistent with the S K-edge XAS data described above that show similar Fe–S covalency for the compounds. The MBOs of the Fe–S bonds are also identical (1.1) between the two compounds, despite the 0.116(2) Å shorter Fe–S bonds and 0.641(2) Å shorter Fe⋯Fe distance in **2** compared to [L^Me^FeS]_2_. Significantly, the Fe–S MBOs in **2** (1.1) are similar to the Fe–S MBO values for the belt sulfides in FeMoco (0.9).^69^ It is also interesting that the Fe⋯Fe MBO in **2** (0.35) is similar to the Fe2⋯Fe6 MBO in FeMoco (0.32).^69^ These comparable MBO values between **2** and FeMoco underscore the electronic similarities between the model complex and the biological cluster site.

**Fig. 9:**
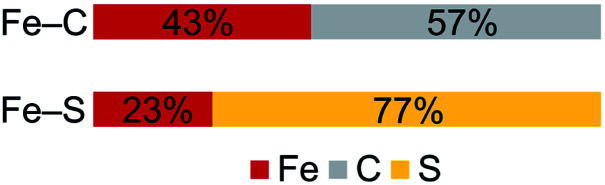
Computed average electron distribution in bonds in **2** from IAOIBO analysis.

In contrast to the copious literature study of Fe–S bonding in FeS clusters,^71–74^ the Fe–C bonding interactions in high-spin complexes remain poorly understood. The incorporation of both a bridging alkylidene and sulfide into **2** allows us to assess the relative covalency of Fe–S and Fe–C bonds. The IAOIBO analysis reveals the average Fe–C electron distribution in the Fe–C bonds of **2** to be 43% Fe and 57% C in character, suggesting that the Fe–C bonds are more covalent than the Fe–S bonds (Fig. 9). Though the limited number of complexes in our studies prevented us from clearly distinguishing the oxidation-state dependence of covalency of Fe–C bonds, the diiron(ii) alkylidene complex **1** reveals a similar total Fe/C electron distribution of 40% Fe and 60% C, which is consistent with the comparable Fe–C MBO values in **1** and **2**. This orbital analysis provides insight into Fe–C bonding in high-spin iron complexes relevant to FeMoco.

## Discussion

The diiron alkylidene sulfide complex **2** presented here incorporates structural elements relevant to nitrogenase, and the Fe/Fe/S2B/C diamond core overlays extremely well with the Fe2/Fe6/S/C rhomb in the resting state of FeMoco (Fig. 2b). Enzymatic studies implicate the Fe2 and Fe6 centers as a primary substrate binding site,^7,8,14,15,75^ and therefore it is particularly important that the core of our synthetic model structurally resembles these centers.

In addition to the local structural similarity, the electronic structure of **2** has significant similarities to this site in FeMoco. First, the oxidation states are identical, based on the diiron(iii) assignment for the Fe2/Fe6 sites in FeMoco based on SpReAD analysis.^30^ Se Kα-HERFD XAS studies on FeMoco with selective substitution of S2B with Se^31^ assign these iron centers as an antiferromagnetically-coupled diferric pair, which is consistent with QM/MM studies.^32^ In **2**, the iron sites have high-spin electronic configurations and display antiferromagnetic coupling between the iron centers, which agrees with calculations indicating that the belt irons Fe2 and Fe6 in the resting state of FeMoco are antiferromagnetically coupled pairs.^30–32^

The comparison of XAS data between **2** and the analogous doubly sulfide-bridged complex [L^Me^Fe(μ-S)]_2_ enables us to evaluate the influence of carbon donors within iron–sulfur clusters. This is significant because there are few other examples of high-spin iron–sulfur clusters with any carbon-based ligands.^46,76,77^ The sulfur pre-edge intensities from the S K-edge XAS data indicate that the marker sulfide does not change its covalency from the addition of the carbon-based bridge. The similarity in Mössbauer isomer shifts of the iron(iii) sites in **2** and its bis-sulfide analogue further emphasizes the similarity of C and S bridges in terms of their influences on the iron centers. Within **2**, though, the Fe–C bonds are more covalent than the Fe–S bonds and form exclusively σ-interactions in contrast to the π interactions contributed by sulfides (see ESI, Fig. S33–S35†). The contracted Fe–C–Fe angle in **2** gives a delocalized orbital that stretches over both iron atoms and the carbon atom (Fig. 8 above), contributing to the greater antiferromagnetic exchange coupling observed in **2**. An analogous superexchange interaction between the iron centers facilitated by the carbon bridge could help to rationalize the different coupling present in FeMoco compared to other FeS clusters.^12,78,79^

Naturally, there are differences between the carbon bridges in these synthetic complexes compared to FeMoco as well. For example, the average Fe–C MBO in complexes **1** and **2** (0.9) are higher than the average Fe–C MBO in FeMoco (0.32).^69^ The variation in the Fe–C MBO between complexes **1** and **2** and the FeMoco may be attributed to the coordination of the carbide to six iron centers (μ_6_) rather than the two iron centers in **1** and **2** (μ_2_).

The ability of the complexes to reduce N_2_ was assessed by adding KC_8_ and HBAr^F^_4_, a mixture that provides a substantial driving force for NH_4_^+^ formation (effective bond dissociation free energy [BDFE] of 0; chemical overpotential of 1220 kJ mol^−1^).^16,27,41,80,81^ The iron complexes **1**, **3**, and **4** reduce N_2_ to NH_4_^+^ to give 1.1–1.4 ± 0.1 equiv. NH_4_^+^ per complex, while complex **2** gives little NH_4_^+^. We ascribe the difference to the open coordination sites in **1** and **3**, while the iron centers in **2** are four-coordinate. The differences in oxidation state are less likely to be important because our N_2_ reduction experiments used a 10-fold excess of reductant relative to the complex. The necessity for a coordinatively unsaturated iron center in these N_2_ reduction studies parallels N_2_ reduction in nitrogenase, as enzymatic studies imply S2B dissociation upon binding substrates.^7,8,14^ It has been hypothesized that dissociation of S2B in the initial stages of FeMoco reduction could be the “trigger” that brings about N_2_ binding and reduction, and in this context we see that the formal loss of an S atom moving from **2** to **1** causes a change from very little N_2_ reduction activity (**2**) to significant N_2_ conversion to NH_4_^+^ (**1**). Thus, these synthetic complexes support the importance of sulfur dissociation for bringing about N_2_ reducing ability in iron–carbon–sulfur clusters.

It is informative to compare the reactivity of **1** and **3** with other diketiminate–iron complexes that lack C and S ligands. We previously reported that addition of four or more equivalents of reductant to [L^Me^Fe(μ-Cl)]_2_ under N_2_ forms complexes M_2_[L^Me^Fe(μ-N_2_)]_3_ (M = K, Rb, Cs).^82^ These clusters, which lack C and S ligands, possess iron centers in lower oxidation states (Fe^0^_2_Fe^1+^) yet they do not form measurable amounts of NH_4_^+^ upon the addition of acid. Similarly, previously reported diketiminate-supported iron complexes containing bridging FeNNFe cores do not react with acid to give NH_4_^+^.^83–85^ In contrast, we see here that the incorporation of C-based ligands and an open coordination site leads to the ability to reduce N_2_ to NH_4_^+^. Though we were unable to deconvolute the influence of the nuclearity and the carbon ligand identity on N_2_ reduction ability in these studies, it appears that the presence of an Fe–C bond is beneficial for N_2_ reduction. We note that two previous carbon-ligated iron systems from Peters and coworkers yield NH_4_^+^ (3.3–4.6 equiv. NH_4_^+^/Fe) as well.^16,27^

Though we isolated the carbon-ligated low-valent species relevant to the N_2_ reduction studies from **3**, the mechanism of N_2_ reduction by the diiron alkylidene complex **1** remains unclear. However, a recent article by Agapie and co-workers described a diiron μ-alkylidyne μ-hydride complex with a Fe/Fe/H/C core, which undergoes Fe–C bond cleavage and C–H bond formation to give various products including iron(ii) alkyl and alkylidene species with N_2_ bound.^17^ These may bear resemblance to the intermediates during N_2_ reduction by **1**. This gains significance because the Fe/Fe/H/C diamond core in the alkylidyne complex is in a more reduced state (closer to the level of N_2_-binding FeMoco intermediates) than the diiron alkylidene sulfide **2**. However, the mechanisms may be different since the strong-field phosphine ligand sphere in the alkylidyne complex is less electronically similar to the FeMoco than the weak-field ligand sphere present in **1**, **2**, and **3** that gives rise to high-spin iron centers.

## Conclusions

This paper has presented a series of complexes that helps to understand the influence of Fe–C bonding on the electronic structure and N_2_ reactivity of high-spin iron sites like those in the FeMoco of nitrogenase. The mononuclear iron(ii) alkyl **3** and the diiron(ii) alkylidene **1** can reduce N_2_ to NH_4_^+^ upon addition of acid and reductant, suggesting that Fe–C bonds are beneficial for N_2_ reduction. Importantly, the lack of a sulfur bridge is essential for N_2_ reduction activity, supporting the idea that sulfur dissociation is a reasonable step toward N_2_ binding in FeMoco.

The structural relevance of **1** to the resting state of FeMoco was extended by incorporating a sulfide ligand to give **2**, which has both a carbon donor and a sulfide as bridges. Complex **2** has nearly identical metrical parameters as the Fe2/Fe6/S2B/C rhomb within the FeMoco resting state structure, and the antiferromagnetic coupling of the iron(iii) centers in **2** aligns well with the analogous coupling of Fe2 and Fe6 in FeMoco. In both dinuclear complexes **1** and **2**, the bridging ligands facilitate electronic communication between the iron centers, giving rise to antiferromagnetic coupling. The addition of a bridging sulfide ligand in **2** enhances the antiferromagnetic coupling interaction. The IAOIBO picture of the Fe–C interactions in **1** and **2** depicts highly covalent Fe–C bonds which can mediate superexchange, suggesting that the presence of a carbon ligand may contribute to the different exchange interactions observed in FeMoco compared to other FeS clusters.

The study of the bonding interactions in these simplified structural models also shows a surprisingly strong similarity between Fe–C bonds and Fe–S bonds. First, S K-edge XAS experiments show that the substitution of an alkylidene for a bridging sulfide minimally influences the Fe–S covalency in the other bridge. Further, the similar Fe–C Mayer bond orders in **1–3** are similar despite differences in the identity of the carbon ligand, the iron oxidation state, and the complex nuclearity. This property of the Fe–C bonds is reminiscent of the well-known similarity of Fe–S interactions in different oxidation states of iron–sulfur clusters.^86^ The insights provided by these model complexes improve our understanding of the Fe–C interactions at high spin iron sites, which helps refine our hypotheses about the structural and electronic implications of the carbide in FeMoco, and the key determinants of N_2_ reduction by the enzyme.

## Conflicts of interest

There are no conflicts to declare.

## Supplementary Material

SC-011-D0SC03447A-s001

SC-011-D0SC03447A-s002

## References

[cit1] Hoffman B. M., Lukoyanov D., Yang Z.-Y., Dean D. R., Seefeldt L. C. (2014). Chem. Rev..

[cit2] Dos Santos P. C., Igarashi R. Y., Lee H.-I., Hoffman B. M., Seefeldt L. C., Dean D. R. (2005). Acc. Chem. Res..

[cit3] George S. J., Barney B. M., Mitra D., Igarashi R. Y., Guo Y., Dean D. R., Cramer S. P., Seefeldt L. C. (2012). J. Inorg. Biochem..

[cit4] Benton P. M. C., Laryukhin M., Mayer S. M., Hoffman B. M., Dean D. R., Seefeldt L. C. (2003). Biochemistry.

[cit5] Lee H.-I., Igarashi R. Y., Laryukhin M., Doan P. E., Dos Santos P. C., Dean D. R., Seefeldt L. C., Hoffman B. M. (2004). J. Am. Chem. Soc..

[cit6] Barney B. M., Igarashi R. Y., Dos Santos P. C., Dean D. R., Seefeldt L. C. (2004). J. Biol. Chem..

[cit7] Spatzal T., Perez K. A., Einsle O., Howard J. B., Rees D. C. (2014). Science.

[cit8] Spatzal T., Perez K. A., Howard J. B., Rees D. C. (2015). eLife.

[cit9] Lancaster K. M., Roemelt M., Ettenhuber P., Hu Y., Ribbe M. W., Neese F., Bergmann U., DeBeer S. (2011). Science.

[cit10] Spatzal T., Aksoyoglu M., Zhang L., Andrade S. L. A., Schleicher E., Weber S., Rees D. C., Einsle O. (2011). Science.

[cit11] Wiig J. A., Lee C. C., Hu Y., Ribbe M. W. (2013). J. Am. Chem. Soc..

[cit12] Rees J. A., Bjornsson R., Kowalska J. K., Lima F. A., Schlesier J., Sippel D., Weyhermüller T., Einsle O., Kovacs J. A., DeBeer S. (2017). Dalton Trans..

[cit13] Čorić I., Mercado B. Q., Bill E., Vinyard D. J., Holland P. L. (2015). Nature.

[cit14] Sippel D., Rohde M., Netzer J., Trncik C., Gies J., Grunau K., Djurdjevic I., Decamps L., Andrade S. L. A., Einsle O. (2018). Science.

[cit15] Kang W., Lee C. C., Jasniewski A. J., Ribbe M. W., Hu Y. (2020). Science.

[cit16] Creutz S. E., Peters J. C. (2014). J. Am. Chem. Soc..

[cit17] Arnett C. H., Agapie T. (2020). J. Am. Chem. Soc..

[cit18] McKee M. L. (2016). J. Phys. Chem. A.

[cit19] Siegbahn P. E. M. (2016). J. Am. Chem. Soc..

[cit20] Siegbahn P. E. M. (2018). J. Comput. Chem..

[cit21] Čorić I., Holland P. L. (2016). J. Am. Chem. Soc..

[cit22] Liu L., Rauchfuss T. B., Woods T. J. (2019). Inorg. Chem..

[cit23] Hickey A. K., Lutz S. A., Chen C.-H., Smith J. M. (2017). Chem. Commun..

[cit24] Lindley B. M., Jacobs B. P., MacMillan S. N., Wolczanski P. T. (2016). Chem. Commun..

[cit25] Lissel F., Schwarz F., Blacque O., Riel H., Lörtscher E., Venkatesan K., Berke H. (2014). J. Am. Chem. Soc..

[cit26] Ouyang Z., Cheng J., Li L., Bao X., Deng L. (2016). Chem.–Eur. J..

[cit27] Ung G., Peters J. C. (2015). Angew. Chem., Int. Ed..

[cit28] Bart S. C., Lobkovsky E., Chirik P. J. (2004). J. Am. Chem. Soc..

[cit29] Ung G., Rittle J., Soleilhavoup M., Bertrand G., Peters J. C. (2014). Angew. Chem., Int. Ed..

[cit30] Spatzal T., Schlesier J., Burger E.-M., Sippel D., Zhang L., Andrade S. L. A., Rees D. C., Einsle O. (2016). Nat. Commun..

[cit31] Henthorn J. T., Arias R. J., Koroidov S., Kroll T., Sokaras D., Bergmann U., Rees D. C., DeBeer S. (2019). J. Am. Chem. Soc..

[cit32] Benediktsson B., Bjornsson R. (2017). Inorg. Chem..

[cit33] Reesbeck M. E., Grubel K., Kim D., Brennessel W. W., Mercado B. Q., Holland P. L. (2017). Inorg. Chem..

[cit34] Reesbeck M. E., Rodriguez M. M., Brennessel W. W., Mercado B. Q., Vinyard D., Holland P. L. (2015). J. Biol. Inorg Chem..

[cit35] DeRosha D. E., Arnet N. A., Mercado B. Q., Holland P. L. (2019). Inorg. Chem..

[cit36] Vela J., Smith J. M., Lachicotte R. J., Holland P. L. (2002). Chem. Commun..

[cit37] Vela J., Vaddadi S., Cundari T. R., Smith J. M., Gregory E. A., Lachicotte R. J., Flaschenriem C. J., Holland P. L. (2004). Organometallics.

[cit38] Rodriguez M. M., Bill E., Brennessel W. W., Holland P. L. (2011). Science.

[cit39] Smith J. M., Lachicotte R. J., Holland P. L. (2002). Organometallics.

[cit40] Sciarone T. J. J., Meetsma A., Hessen B. (2006). Inorg. Chim. Acta.

[cit41] Fajardo J., Peters J. C. (2017). J. Am. Chem. Soc..

[cit42] MacLeod K. C., McWilliams S. F., Mercado B. Q., Holland P. L. (2016). Chem. Sci..

[cit43] Battino R., Rettich T. R., Tominaga T. (1984). J. Phys. Chem. Ref. Data.

[cit44] Gibanel F. L., López M. C., Royo F. M., Pardo J., Urieta J. S. (1993). Fluid Phase Equilib..

[cit45] McWilliams S. F., Bill E., Lukat-Rodgers G., Rodgers K. R., Mercado B. Q., Holland P. L. (2018). J. Am. Chem. Soc..

[cit46] Suess D. L. M., Tsay C., Peters J. C. (2012). J. Am. Chem. Soc..

[cit47] McWilliams S. F., Broere D. L. J., Halliday C. J. V., Bhutto S. M., Mercado B. Q., Holland P. L. Nature.

[cit48] Münck E., Aspects of ^57^Fe Mössbauer Spectroscopy, in Physical Methods in Bioinorganic Chemistry, ed. L. Que Jr, University Science Books, New York, 2000, pp. 287–319

[cit49] McWilliams S. F., Brennan-Wydra E., MacLeod K. C., Holland P. L. (2017). ACS Omega.

[cit50] Pollock C. J., Tan L. L., Zhang W., Lancaster K. M., Lee S. C., DeBeer S. (2014). Inorg. Chem..

[cit51] Marlin D. S., Olmstead M. M., Mascharak P. K. (2000). Inorg. Chim. Acta.

[cit52] Werncke C. G., Pfeiffer J., Müller I., Vendier L., Sabo-Etienne S., Bontemps S. (2019). Dalton Trans..

[cit53] Yao S., Meier F., Lindenmaier N., Rudolph R., Blom B., Adelhardt M., Sutter J., Mebs S., Haumann M., Meyer K., Kaupp M., Driess M. (2015). Angew. Chem., Int. Ed..

[cit54] McWilliams S. F., Bunting P. C., Kathiresan V., Mercado B. Q., Hoffman B. M., Long J. R., Holland P. L. (2018). Chem. Commun..

[cit55] Stoian S. A., Vela J., Smith J. M., Sadique A. R., Holland P. L., Münck E., Bominaar E. L. (2006). J. Am. Chem. Soc..

[cit56] Andres H., Bominaar E. L., Smith J. M., Eckert N. A., Holland P. L., Münck E. (2002). J. Am. Chem. Soc..

[cit57] Chiang K. P., Barrett P. M., Ding F., Smith J. M., Kingsley S., Brennessel W. W., Clark M. M., Lachicotte R. J., Holland P. L. (2009). Inorg. Chem..

[cit58] Christou G., Gatteschi D., Hendrickson D. N., Sessoli R. (2011). MRS Bull..

[cit59] Stoian S. A., Yu Y., Smith J. M., Holland P. L., Bominaar E. L., Münck E. (2005). Inorg. Chem..

[cit60] Hendrich M. P., Gunderson W., Behan R. K., Green M. T., Mehn M. P., Betley T. A., Lu C. C., Peters J. C. (2006). Proc. Natl. Acad. Sci. U. S. A..

[cit61] Gütlich P., Bill E. and Trautwein A. X., Mössbauer spectroscopy and transition metal chemistry: fundamentals and applications, Springer-Verlag, Berlin, 2010

[cit62] Neese F., Hedman B., Hodgson K. O., Solomon E. I. (1999). Inorg. Chem..

[cit63] David G., Wennmohs F., Neese F., Ferré N. (2018). Inorg. Chem..

[cit64] Neese F. (2009). Coord. Chem. Rev..

[cit65] Neese F., Petrenko T., Ganyushin D., Olbrich G. (2007). Coord. Chem. Rev..

[cit66] Yogendra S., Weyhermüller T., Hahn A. W., DeBeer S. (2019). Inorg. Chem..

[cit67] Knizia G. (2013). J. Chem. Theory Comput..

[cit68] Bridgeman A. J., Cavigliasso G., Ireland L. R., Rothery J. (2001). J. Chem. Soc., Dalton Trans..

[cit69] Benediktsson B., Bjornsson R. (2020). Inorg. Chem..

[cit70] Yang L., Powell D. R., Houser R. P. (2007). Dalton Trans..

[cit71] Harris T. V., Szilagyi R. K. (2014). J. Comput. Chem..

[cit72] Kowalska J. K., Hahn A. W., Albers A., Schiewer C. E., Bjornsson R., Lima F. A., Meyer F., DeBeer S. (2016). Inorg. Chem..

[cit73] Dey A., Roche C. L., Walters M. A., Hodgson K. O., Hedman B., Solomon E. I. (2005). Inorg. Chem..

[cit74] Mascharak P. K., Armstrong W. H., Mizobe Y., Holm R. H. (1983). J. Am. Chem. Soc..

[cit75] Seefeldt L. C., Dance I. G., Dean D. R. (2004). Biochemistry.

[cit76] Ye M., Thompson N. B., Brown A. C., Suess D. L. M. (2019). J. Am. Chem. Soc..

[cit77] Scott T. A., Berlinguette C. P., Holm R. H., Zhou H.-C. (2005). Proc. Natl. Acad. Sci. U. S. A..

[cit78] Thorhallsson A. T., Benediktsson B., Bjornsson R. (2019). Chem. Sci..

[cit79] Bjornsson R., Neese F., Schrock R. R., Einsle O., DeBeer S. (2015). J. Biol. Inorg Chem..

[cit80] Pappas I., Chirik P. J. (2016). J. Am. Chem. Soc..

[cit81] van der Ham C. J. M., Koper M. T. M., Hetterscheid D. G. H. (2014). Chem. Soc. Rev..

[cit82] Grubel K., Brennessel W. W., Mercado B. Q., Holland P. L. (2014). J. Am. Chem. Soc..

[cit83] Smith J. M., Sadique A. R., Cundari T. R., Rodgers K. R., Lukat-Rodgers G., Lachicotte R. J., Flaschenriem C. J., Vela J., Holland P. L. (2006). J. Am. Chem. Soc..

[cit84] Dugan T. R., MacLeod K. C., Brennessel W. W., Holland P. L. (2013). Eur. J. Inorg. Chem..

[cit85] Smith J. M., Lachicotte R. J., Pittard K. A., Cundari T. R., Lukat-Rodgers G., Rodgers K. R., Holland P. L. (2001). J. Am. Chem. Soc..

[cit86] Beinert H., Holm R. H., Münck E. (1997). Science.

