# Supplementary material for: The influences of carbon donor ligands on biomimetic multi-iron complexes for N2 reduction
Source: Chem Sci. 2020 Aug 6;11(47):12710–20. doi: 10.1039/d0sc03447a (PMC8163302; doi:10.1039/d0sc03447a)
Supplement: SC-011-D0SC03447A-s001 [file SC-011-D0SC03447A-s001.pdf]

Electronic Supplementary Material (ESI) for Chemical Science.
This journal is © The Royal Society of Chemistry 2020

Electronic Supplementary Information for

The Influence of Carbon Donor Ligands on Biomimetic Iron Complexes for N_2 Reduction

Alexandra L. Nagelski, Majed S. Fataftah, Melissa M. Bollmeyer, Sean F. McWilliams, Samantha N. MacMillan, Brandon Q. Mercado, Kyle M. Lancaster, and Patrick L. Holland

Table of Contents

Synthesis and Characterization **S2**
General considerations S2
Improved Synthesis of **1** S3
Synthesis of **2** S3
Synthesis of **3** S3
Synthesis of **4** S4
Discussion of **4–N$_2$** Structure S4
Ammonia Production Studies S5
Discussion of Active Species in N_2 Reduction of **1** S6
^{1}H NMR Spectra **S7**
Van't Hoff Analysis of **4** **S17**
Et_2O-d_{10} under N_2 ..... S17
THF-d_8 under N_2 ..... S19
UV-Visible Spectra **S24**
Mössbauer Spectra **S29**
IR Spectra **S32**
Magnetic Measurements **S35**
Experimental details S35
Computations **S41**
XAS Spectra **S53**
Data Collection S53
Data Processing S53
Crystallographic Data for **2–4** S59
Discussion of τ_4 Parameter in **1** and **2** S67
References S68

General Considerations. Unless otherwise noted, all manipulations were performed under an N_2 atmosphere using Schlenk techniques or in an M. Braun glovebox maintained at or below 1 ppm of O_2 and H_2O. Glassware was dried at 150 °C overnight. Celite, alumina, and 4 Å molecular sieves were dried at 200 °C under vacuum overnight. Pentane, hexanes, diethyl ether, and toluene were dried and scrubbed of O_2 by passage through activated alumina and activated Q5 columns from Glass Contour Co. THF was degassed by sparging for 15 minutes, and dried by passage through two activated alumina columns from Inert. All solvents were stored over activated 4 Å molecular sieves. Benzene-d_6 was dried and stored over activated alumina and then filtered before use. Et_2O-d_{10} and THF-d_8 were dried by vacuum transfer from potassium benzophenone ketyl solution and were stored over 4 Å molecular sieves.

Trimethylsilyldiazomethane ($N_2CHSiMe_3$, 2.0 M in hexanes) was purchased from Acros and Alfa Aesar and used as received. Trimethylsilylmethylmagnesium chloride (1.3 M in THF) was purchased from Acros and used as received. Isotopically labeled $^{15}N_2$ (98%) was purchased from Cambridge Isotope Laboratories. The following compounds were prepared according to published procedures: $[L^{Me}FeCl]_2$,[1] $[L^{Me}FeS]_2$,[2] KC_8,[3] $HBAr^F{}_4$,[4] $SPMe_3$,[5] and $L^{Me}H$.[1]

^1H NMR data were recorded on Agilent DD2 400 MHz, 500 MHz, or 600 MHz spectrometers. All resonances in the ^1H NMR spectra are referenced to residual protio solvents: benzene (δ 7.16 ppm), toluene (δ 2.09 ppm), or THF (δ 3.58 or 1.73 ppm). Resonances were singlets unless otherwise noted. IR data were recorded on a Bruker ALPHA spectrometer equipped with a Platinum-ATR attachment. UV-vis spectra were recorded on a Cary 60 spectrophotometer using Schlenk-adapted S-3 quartz cuvettes with a 1 cm or 2 mm optical path length, and cooled with a Unisoku CoolSpek UV USP-203A cryostat. Absorbances have been corrected for solvent density change with temperature.[6] Solution magnetic susceptibilities were determined by the Evans method.[7] Mössbauer measurements were recorded on a SeeCo MS4 Mössbauer spectrometer with alternating constant acceleration; isomer shifts are relative to iron metal at 298 K. The sample temperature was maintained in a Janis Research Company Inc. cryostat. The zero-field spectra were simulated by using Lorentzian doublets with Γ representing the line width fitting parameter. Elemental analyses were obtained from the CENTC Elemental Analysis Facility at the University of Rochester. Microanalysis samples were weighed with a PerkinElmer Model AD-6 Autobalance and their compositions were determined with a PerkinElmer 2400 Series II Analyzer, and handled in a VAC Atmospheres glovebox under argon.

EPR spectra were collected at X-band frequency (9.43 GHz) using a Bruker ELEXYS E500 spectrometer equipped with a SHQ resonator. The data were collected on samples of **4** under either an N_2 and Ar atmosphere, and flash frozen in liquid nitrogen at 77 K. The following parameters were used: microwave frequency, 9.436 GHz; modulation frequency, 100 KHz, modulation amplitude, 5 G; time constant, 10 ms; time sweep, 5.12 ms; microwave power, 0.12 mW. Data were simulated using EasySpin with the spin Hamiltonian $\hat{H} = D\hat{S}_z^2 + E(\hat{S}_x^2 - \hat{S}_y^2) + (g_x+g_y+g_z)\beta \boldsymbol{SH}$.[8] Simulation of the EPR spectrum of **4** was performed using the pepper function, and employed the following parameters: $|D| = 12.9$ cm^{-1}, $|E| = 1.7$ cm^{-1}, $g_x = 2.36$, $g_y = 2.33$, $g_z = 2.05$. The simulation was performed with a Voigtian lineshape, using a 5 mT Gaussian contribution, and a 20 mT Lorentzian contribution.

Improved Synthesis of $[L^{Me}Fe]_2(\mu\text{-}CHSiMe_3)$ (1).[9] A solution of $L^{Me}Fe(C_6H_6)$ (202.0 mg, 0.4455 mmol) was dissolved in THF (20 mL) and frozen in a –196 °C coldwell. Upon thawing, trimethylsilyldiazomethane (115 µL, 2.0 M in hexanes) was added dropwise to the stirring solution. The reaction solution was stirred while warming to room temperature for *ca.* 40 min, then placed under reduced pressure to remove the volatile materials. The reaction solid was dissolved in pentane (24 mL) and filtered through a glass frit covered with Celite. Concentration of this pentane solution to 10 mL and cooling to –42 °C for 6 hours gave brown crystals (117.9 mg, 62% yield). The ^1H NMR spectrum matched the literature.[9] Anal. Calcd for $C_{48}H_{64}Fe_2N_4Si$ (**1**): C, 68.89, H, 7.71, N, 6.70. Found: C, 68.89, H, 7.96, N, 6.85.

Synthesis of $[L^{Me}Fe]_2(\mu\text{-}S)(\mu\text{-}CHSiMe_3)$ (2). $SPMe_3$ (7.1 mg, 0.066 mmol) in THF (1.4 mL) was added dropwise to a thawing solution of **1** (55 mg, 0.066 mmol) in THF (3.4 mL), which caused an instant color change from amber to forest green. The reaction solution was stirred at room temperature for 35 minutes and the volatile materials were removed under vacuum. The reaction residue was extracted with Et_2O (16 mL) and filtered through a pipet filter containing a pad of Celite. The filtrate was placed under reduced pressure to remove the volatile materials, which yielded a green powder (51 mg, 89%). A solution of **2** in pentane (10 mL) was cooled to –42 °C for 2 days to give crystals suitable for X-ray analysis. ^1H NMR (500 MHz, C_6D_6, 298K, Fig. S2): 22 (6H, α-CH_3), 8.8 (4H, *meta*-CH or *para*-CH), 8.7 (4H, *meta*-CH or *para*-CH), 4.4 (12H, β-CH_3 or *ortho*-CH_3), 3.9 (12H, β-CH_3 or *ortho*-CH_3), 2.2 (9H, $SiMe_3$), 0.1 (12H, β-CH_3 or *ortho*-CH_3) ppm. One 4H resonance for a *meta*-CH or *para*-CH environment, and the CH on the bridging alkylidene, were not observed. These may be broadened or hidden under other peaks. ^1H NMR (500 MHz, THF-d_8, 298 K, Fig. S1): 22 (6H, α-CH_3), 8.8 (4H, *meta*-CH or *para*-CH), 8.6 (4H, *meta*-CH or *para*-CH), 4.2 (12H, β-CH_3 or *ortho*-CH_3), 4.0 (4H, *meta*-CH or *para*-CH), 3.7 (12H, β-CH_3 or *ortho*-CH_3), 1.8 (9H, $SiMe_3$), 0.2 (12H, β-CH_3 or *ortho*-CH_3) ppm. One 4H resonance for a *meta*-CH or *para*-CH environment, and the CH on the bridging alkylidene, were not observed. These may be broadened or hidden under other peaks. Zero-field Mössbauer (solid, 80 K, Fig. S22): δ = 0.26 mm s^{-1}, $|\Delta E_Q|$ = 1.95 mm s^{-1}, and $\Gamma_L = \Gamma_R$ = 0.28 mm s^{-1}. IR (solid, ATR, cm^{-1}, Fig. S25): 2956 (w), 2915 (w), 2853 (w), 1515 (m), 1461 (m), 1410 (m), 1319 (m), 1187 (m), 1091 (m), 963 (m), 826 (m), 763 (w), 619 (w), 507 (m), 409 (m). Anal. Calcd for $C_{48}H_{64}Fe_2N_4SiS$ (**2**): C, 66.35, H, 7.42, N, 6.45. Found: C, 65.40, H, 7.56, N, 6.15. Unfortunately, we were unable to isolate this compound in greater than 94% purity (evidenced from the presence of a 6% iron impurity visible in the Mössbauer spectrum; this impurity was not found in the ^1H NMR spectra).

Synthesis of $L^{Me}FeCH_2SiMe_3$ (3). Trimethylsilylmethylmagnesium chloride solution (0.46 mL of 1.3 M solution in THF) was added to a solution of $[L^{Me}FeCl]_2$ (0.233 g, 0.284 mmol) in THF (6 mL), resulting in an immediate color change from green-yellow to red. The reaction mixture was stirred overnight at room temperature. Dioxane (0.2 mL) was added to the reaction and stirred for 45 min. The volatile materials were removed under reduced pressure. The residue was extracted with pentane (8 mL) and removed under vacuum twice, then dissolved in hexanes (10 mL) and passed through a glass frit covered with Celite. The filtrate was concentrated to 3 mL and cooled to –42 °C for 4 h to yield bright orange crystals of **3** (0.162 g, 62% yield). ^1H NMR (400 MHz, C_6D_6,

298K, Fig. S4): 182 (3H, α-CH_3), 69 (6H, β-CH_3), 36 (9H, $SiMe_3$), -5 (4H, *meta*-CH), -59 (12 H, *ortho*-CH_3), -86 (2H, *para*-CH) ppm. ^1H NMR (500 MHz, THF-d_8, 298K, Fig. S3): 143 (3, α-CH_3), 17 (12H, *ortho*-CH_3), 14 (4H, *meta*-CH), 4.2 (9H, $SiMe_3$), -52 (2H, *para*-CH), -54 (6H, β-CH_3) ppm. Zero-field Mössbauer (solid, 80 K, Fig. S23): $\delta = 0.43$ mm s^{-1}, $|\Delta E_Q| = 1.28$ mm s^{-1}, $\Gamma_L = 0.39$, $\Gamma_R = 0.35$ mm s^{-1}. μ_{eff} (Evans, C_6D_6, 298 K) = 5.4(2) μ_B. IR (solid, ATR, cm^{-1}, Fig. S26): 2935 (m), 1520 (w), 1460 (m), 1412 (m), 1321 (w), 1236 (m), 1191 (m), 1091 (m), 985 (m), 875 (m), 864 (w), 846 (m), 815 (m), 766 (m), 710 (m), 671 (m), 511 (m). Anal. Calcd for $C_{26}H_{38}FeN_2Si$ (**3**): C, 67.52; H, 8.28; N, 6.06; Found: C, 67.43, H, 8.43; N, 5.92.

Synthesis of [$L^{Me}FeCH_2SiMe_3$][K(18-crown-6)] (4). A solution of 18-crown-6 (19.7 mg, 0.075 mmol) in THF (0.8 mL) was added to a stirring solution of $L^{Me}FeCH_2TMS$ (30.0 mg, 0.065 mmol) in THF (3.0 mL). The resulting solution of 18-crown-6 and **3** was added to KC_8 (10.0 mg, 0.074 mmol) and an immediate color change from orange to green was observed. The reaction was stirred at room temperature for 20 min and the volatile materials were removed under reduced pressure. The solid was rinsed with Et_2O (4 mL) and passed through a Celite pipet filter plugged with glass wool. The remaining residue was extracted with THF (3.4 mL) and passed through the same Celite pipet filter. The THF fraction was placed under reduced pressure to remove volatile materials. The resulting solid was rinsed with pentane (16 mL) and collected on a M frit (32 mg, 55% yield). ^1H NMR spectrum (500 MHz, THF-d_8, 298 K, Fig. S5): 138 (3H, α-CH_3), 37 (12H, *ortho*-CH_3), 31 (4H, *meta*-CH), 3.14 (24H, 18-crown-6), -4.48 (10H, $SiMe_3$). We were not able to locate the 6 protons corresponding to the β-CH_3 of the ligand. Zero-field Mössbauer (solid, 80 K, Fig. S24): $\delta = 0.41$ mm s^{-1}, $|\Delta E_Q| = 2.23$ mm s^{-1}, $\Gamma_L = 0.35$, $\Gamma_R = 0.41$ mm s^{-1}. IR (solid, ATR, cm^{-1}, Fig. S27): 2895 (m), 1584 (w), 1455 (m), 1417 (m), 1377 (m), 1351 (w), 1321 (m), 1248(m), 1197 (m), 1103 (m), 960 (w), 859 (m), 830 (m), 760 (m), 712(m), 663 (m), 490 (m). Anal. Calcd for $C_{38}H_{62}FeN_2SiO_6K$ (**4**): C, 59.59; H, 8.16; N, 3.66; Found: C, 59.26 H, 8.30; N, 3.34.

Discussion of Complex 4–N_2 Structure and Binding. We were unable to crystallize **4-N_2**. We hypothesize that N_2 binds to **4** in a terminal κ^1 fashion due to the similarity of its ^1H NMR and UV-vis spectra to the related complex [$L^{Me,iPr}FePh$][Na(15-crown-5)] which was crystallographically characterized with κ^1-bound N_2.[10] Similar mononuclear complexes have demonstrated N_2 bridging to a κ^1-bound alkali cation.[11] Alkali cations can aid N_2 polarization to increase charge transfer from the iron center, resulting in stabilization of the N_2 π^* orbital.[12] We speculate that the difference in N_2 binding affinity by solvent can be explained by considering solvent coordination to the (18-crown-6)K^+ cation.

Standard N_2 Reduction Procedure. The iron complex (16 μmol) was dissolved in Et_2O (6 mL) and added to a 100 mL resealable flask equipped with a stir bar. The solvent was removed under reduced pressure to give a solid film of complex. KC_8 (21 mg, 0.16 mmol) was suspended in Et_2O (2.5 mL) and added to the reaction at –100 °C. The reaction was stirred for 40 min at –100 °C and frozen in a –196 °C cold well. $HBAr^F_4$ (162 mg, 0.16 mmol) in Et_2O (1.0 mL) was added dropwise to the reaction flask. The reaction was stirred at –78 °C for 1 h, followed by stirring at room temperature for 45 min.

Ammonia Quantification. The reaction flask was cooled to –196 °C. Under a positive flow of N_2, a solution of NaO*t*Bu (25–30 mg) in CH_3OH (2 mL) was added dropwise to the flask in a –196 °C bath over 5 min. The flask was sealed and stirred at room temperature for 30 min, then frozen in a –196 °C bath and the headspace was evacuated. The volatile materials in the reaction mixture were vacuum transferred into a 50 mL bomb flask charged with HCl (3.0 mL of a 2.0 M solution in Et_2O). After the majority of the volatile materials transferred to the collection flask, the flask containing the reaction mixture was heated to 100 °C for 30 min to ensure that ammonia transfer was complete.*[13] The collection flask was sealed and stirred vigorously at room temperature for 30 minutes. The volatile materials were removed under reduced pressure to yield solid NH_4Cl. The indophenol method was used for quantification of NH_4^+ in this product.[14]
*Previous experiments demonstrated that vacuum transfer without heating was not sufficient to drive NH_3 off, resulting in apparent lower NH_3 yields.[13] Heating the reaction mixture to 100 °C for 20 minutes during the vacuum transfer improved the yields. Similarly, control experiments of the ammonia quantification were performed with stock solutions of NH_4Cl and additionally showed that heating the reaction flask during the vacuum transfer was crucial to obtain up to 96% of expected NH_4^+.

Procedure for formation of $^{15}NH_4Cl$. In a nitrogen-filled glovebox, the complex (16 μmol) was quantitatively transferred in Et_2O (6 mL) into a 25 mL three-neck flask (ground glass, 14/20) equipped with a stir bar. A stopcock adaptor was placed in the side opening, a solid addition arm containing solid KC_8 (21 mg, 0.16 mmol) was placed in the other side opening, and a stopper in the central position. The volatile materials were removed under vacuum to leave a thin film. In an argon-filled glovebox, the complex was dissolved in Et_2O (2.0 mL), and the stopper in the central opening was replaced with a 59.61 mL gas addition bulb filled with 1 atm of $^{15}N_2$. The flask was then frozen in a –196 °C cold well and the headspace was evacuated. The flask was then allowed to warm to room temperature and the gas addition bulb was opened to the reaction flask. The flask was cooled to –100 °C and solid KC_8 was added. The flask was stirred at –100 °C for 1 h and then frozen in a liquid N_2 coldwell. A solution of $HBAr^F_4$ (162 mg, 0.160 mmol) in Et_2O (1.0 mL) was added through the stopcock adaptor and the flask was frozen at –196 °C. The reaction mixture was stirred at –78 °C for 1 h, then at room temperature for 45 min. The flask was again frozen at –196 °C bath. Over a flow of N_2, NaO*t*Bu (25–30 mg, *ca.* 0.26 mmol) in CH_3OH (2 mL) was added dropwise to the flask in the –196 °C bath over 5 min. The flask was sealed and stirred at room temperature for at least 30 min, then frozen in a –196 °C bath and the headspace was evacuated. The volatile materials in the reaction mixture were vacuum transferred into a 50 mL bomb flask charged with HCl (3.0 mL of a 2.0 M solution in Et_2O). After the majority of the volatile materials

transferred to the collection flask, the flask containing the reaction mixture was heated to 100 °C for 30 min. The collection flask was sealed and stirred vigorously at room temperature for 30 min. The volatile materials were removed under reduced pressure to yield solid NH_4Cl. The presence of $^{15}NH_4Cl$ was verified by ^1H NMR spectroscopy in DMSO-d_6 (see Fig. S6-7).

Table S1 Ammonium Yields of Control Experiments under Ar in Et_2O

Complex	NH_4^+ Per Complex	% Yield Per Complex	% Yield Per Fe
**1**	0.09	4	2
**3**	0.07	3	3

Discussion of Active Species in N_2 Reduction of Complex 1

To explore the species responsible for NH_4^+ production from **1**, we conducted low temperature ^1H NMR studies with smaller amounts of reductant and acid (Fig. S16). Reduction of **1** with KC_8 in THF-d_8 at –70 °C showed the formation of trace amounts (<10%) of **1** and **3**, but most of the mixture consisted of unidentified species that we were unable to isolate. Protonation of **1** with 1 equiv $HBAr^F_4$ at –70 °C over 1 h resulted in approximately 10% of **3**, along with a mixture of unreacted **1** and an observed unknown species. These results suggest that the alkylidene bridge in **1** is cleaved during protonation, though the low yields prevent us from drawing reliable structural conclusions. Further, the treatment of **1** with 1.2 equiv of 18-crown-6, KC_8 and $HBAr^F_4$ in THF-d_8 at –70 °C formed a small amount of **3**, with approximately half of **1** remaining unreacted. Other iron-containing species are observed in the ^1H NMR spectra of these reactions, but we were unable to isolate and characterize them. As a result, we cannot confidently attribute the N_2 reduction by **1** to any one active species. However, we reason that since the conversion of **1** to **3** with protonation/reduction is only 10%, the observed N_2 reduction activity of **1** cannot be solely attributed to the formation of **3** under these conditions. It is therefore evident that there is some reduced form of **1** (or a degradation product therefrom) that is capable of binding N_2 and reducing it to NH_4^+.

^{1}H NMR Spectra

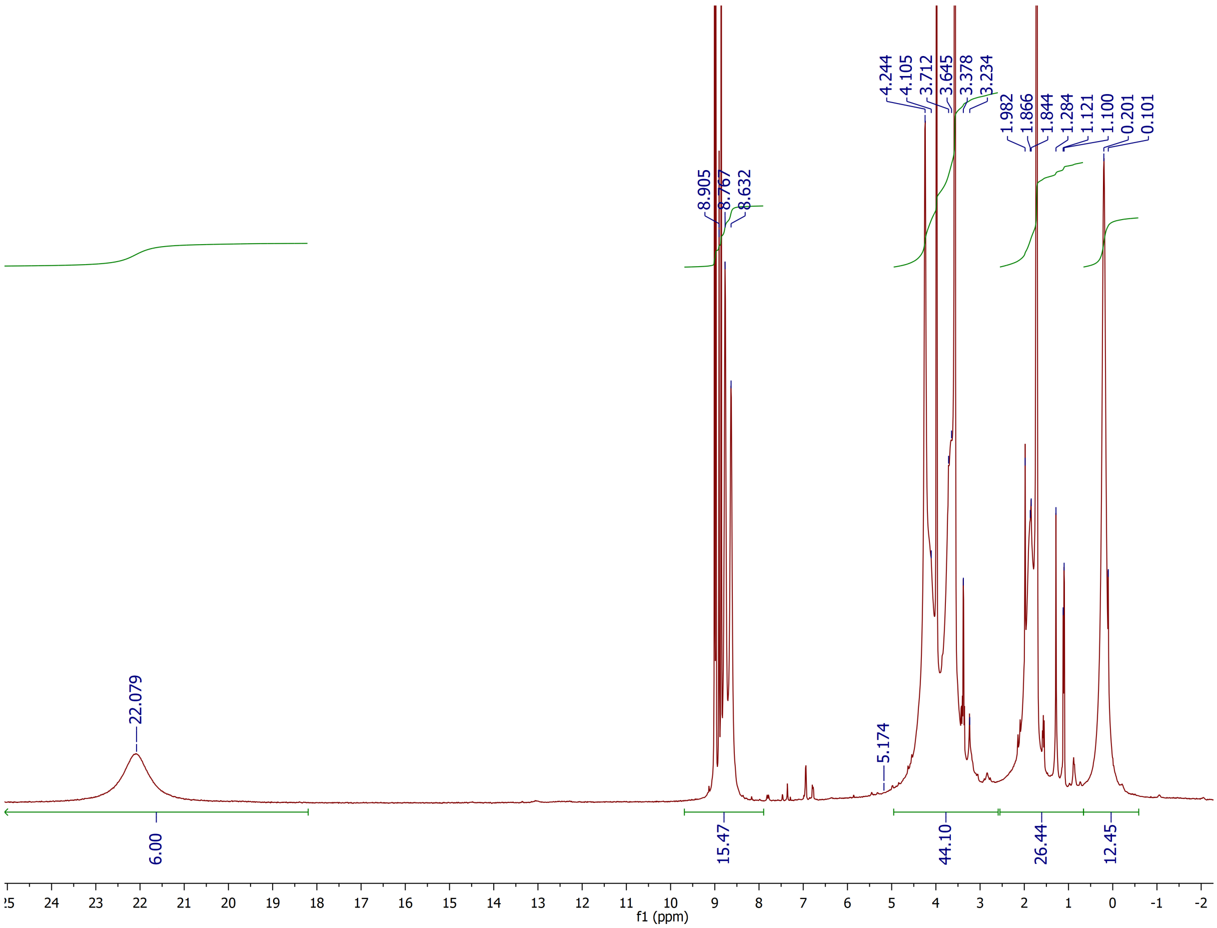

Figure S1. ^{1}H NMR spectrum of **2** in THF-d_8. The peaks from 8.8–9 ppm are from degradation from a nickelocene capillary placed in the NMR tube.

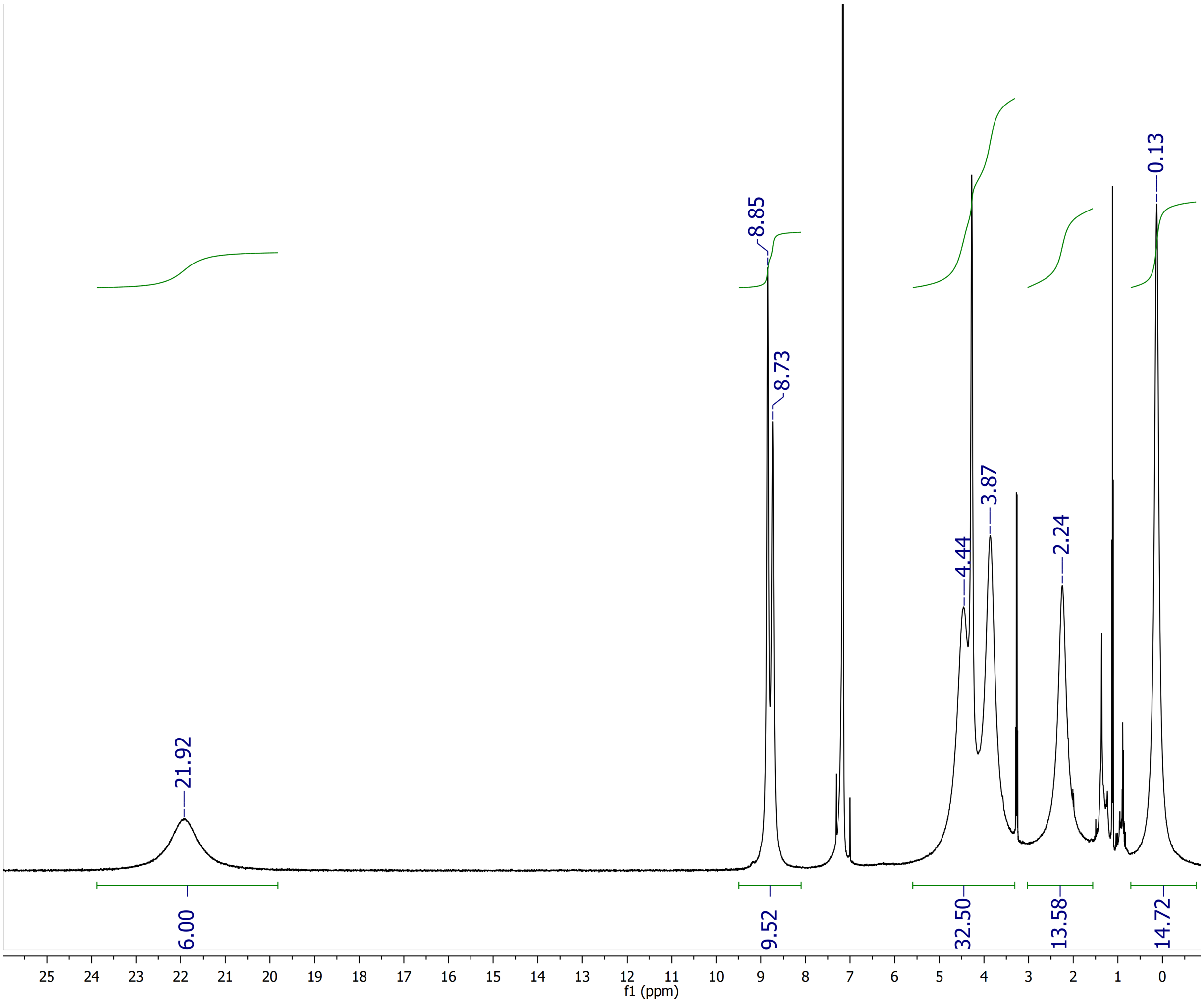

Figure S2. ^{1}H NMR spectrum of **2** in C_6D_6

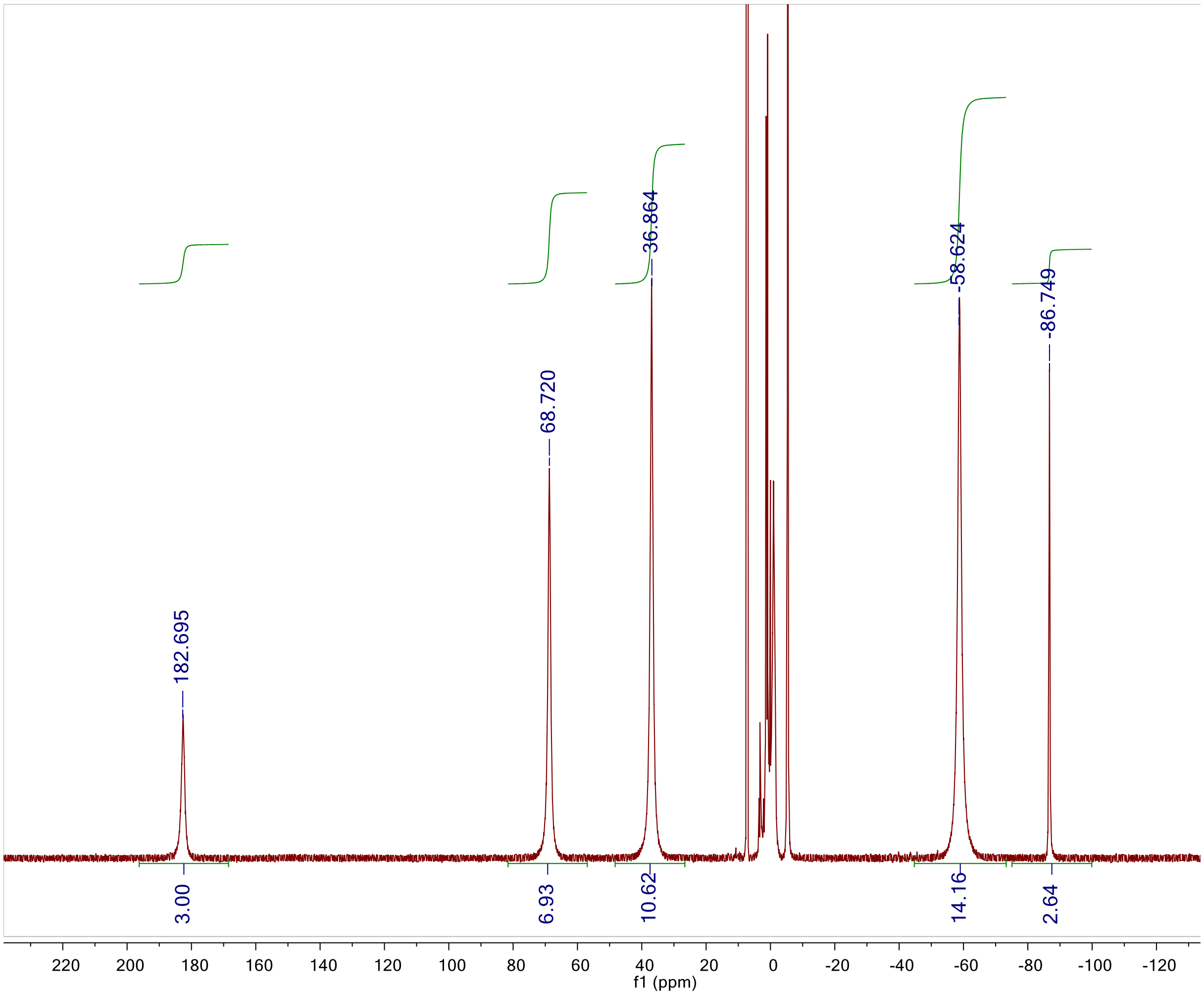

Figure S3. ^{1}H NMR spectrum of **3** in THF-d_8

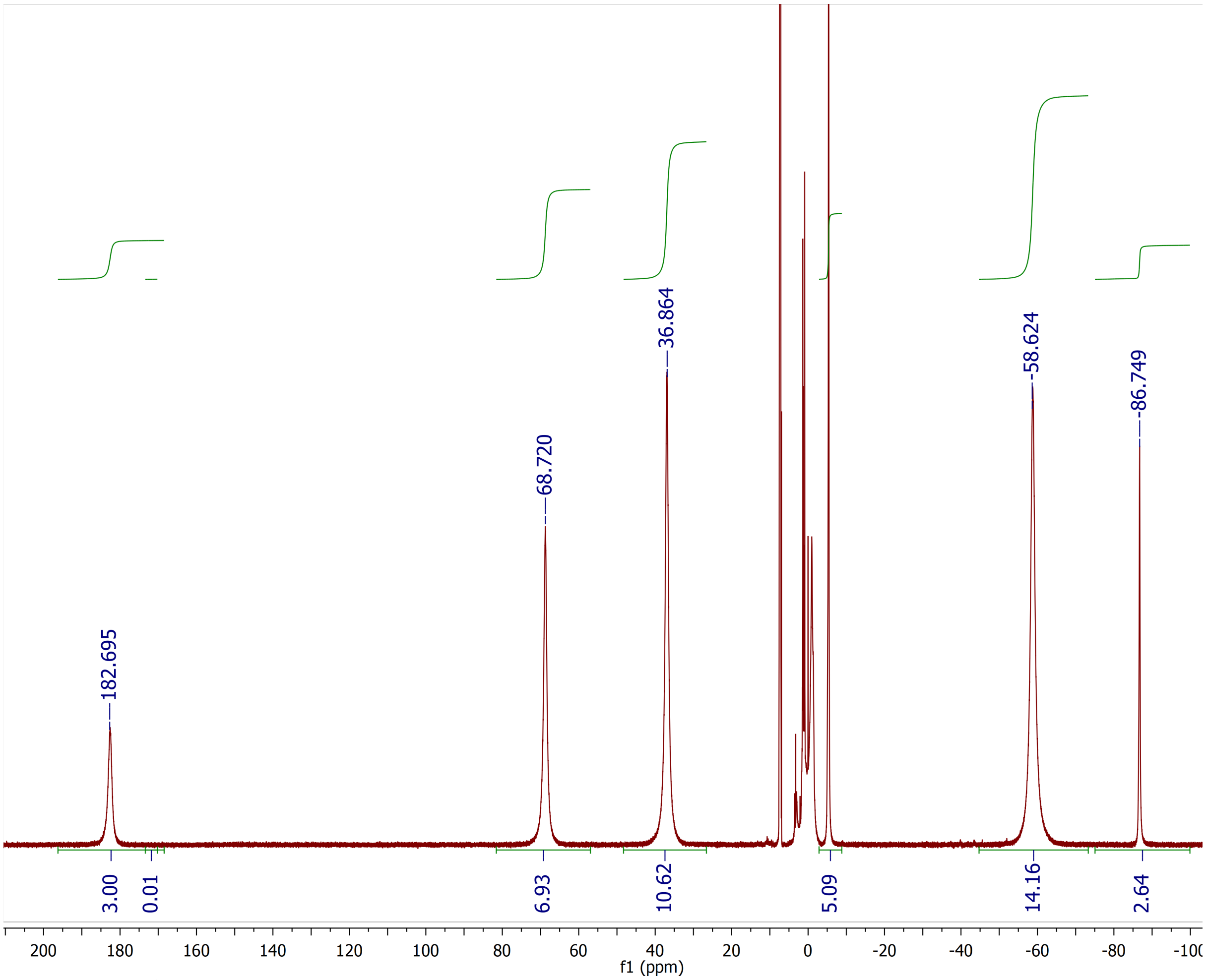

Figure S4. ^1H NMR spectrum of **3** in C_6D_6

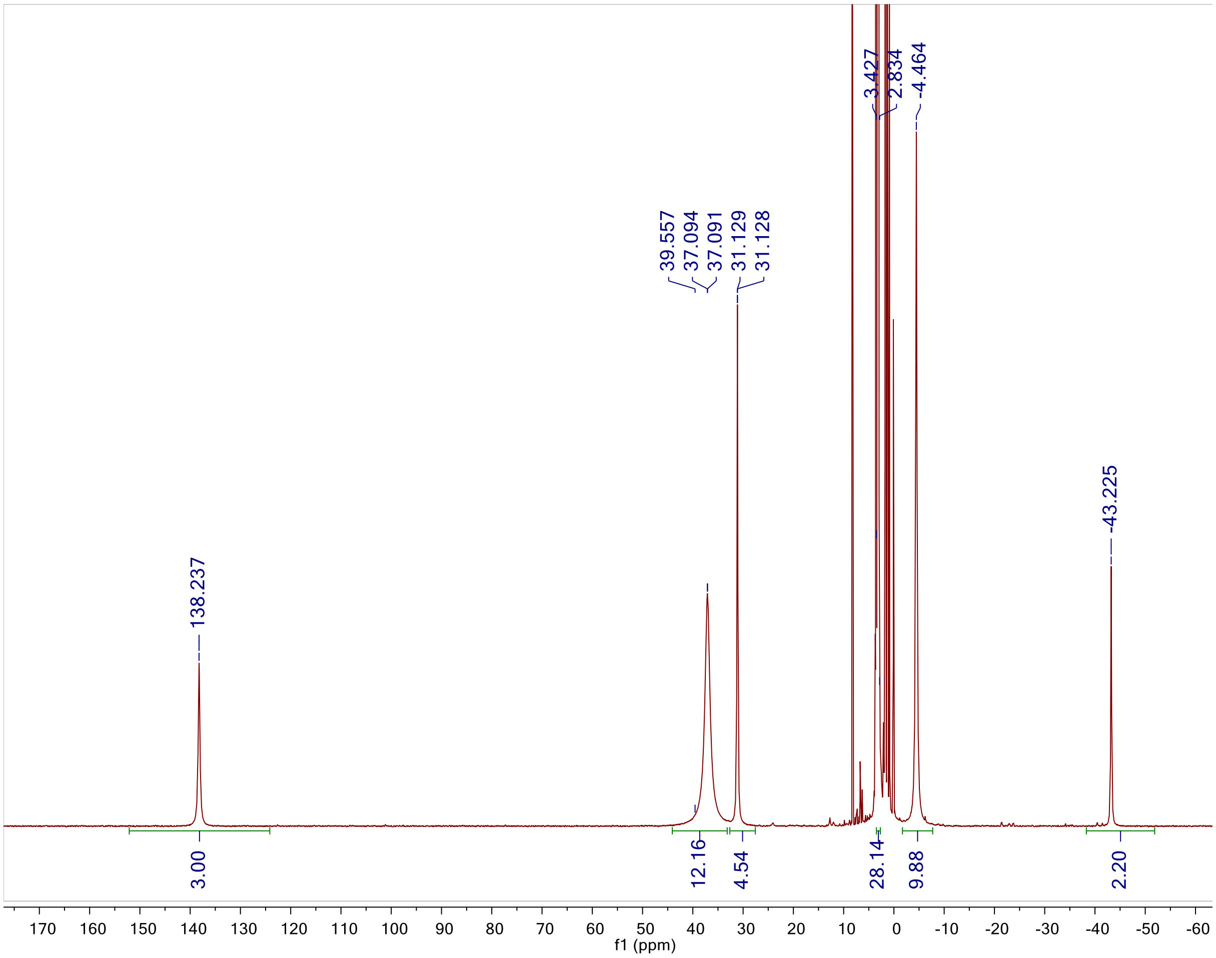

Figure S5. ^{1}H NMR spectrum of **4** in THF-d_8

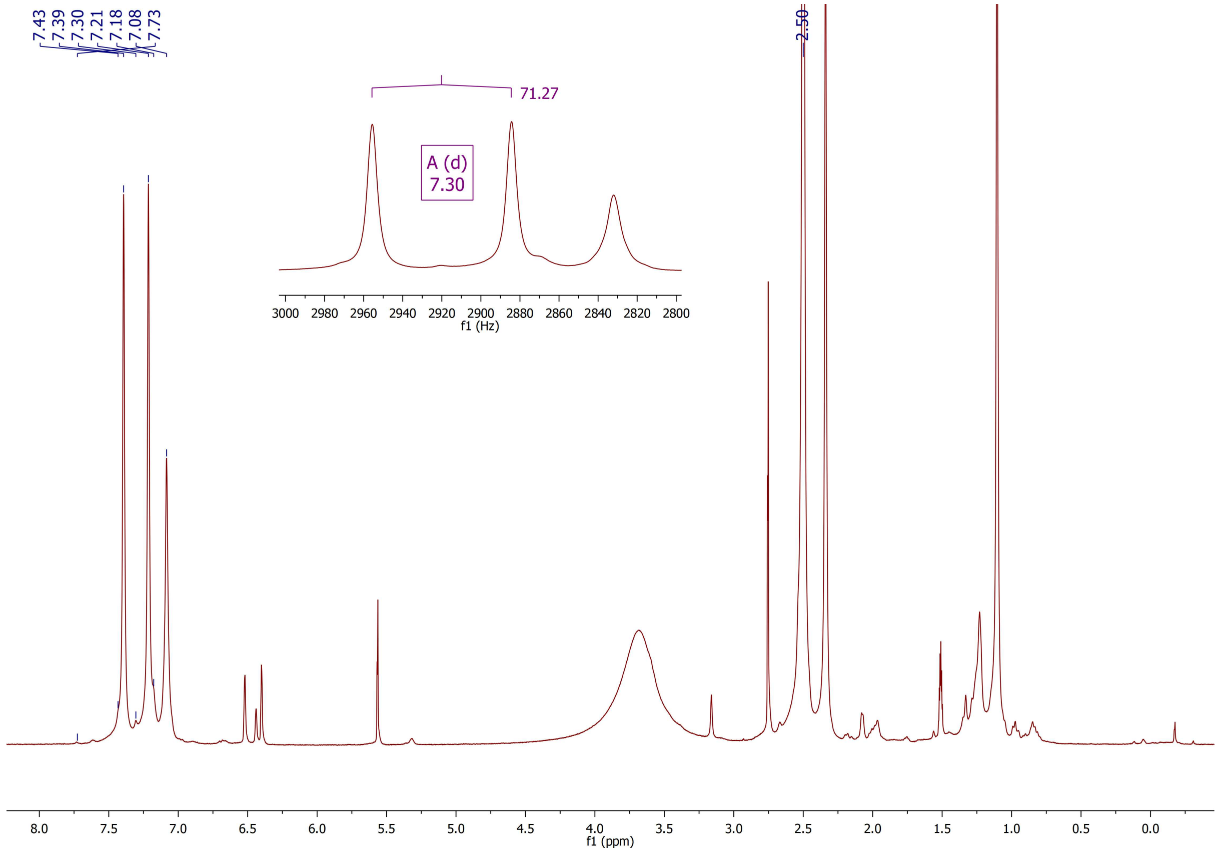

Figure S6. ^{1}H NMR spectrum of $^{15}NH_4Cl$ from $^{15}N_2$ reduction experiment of **1** in DMSO-d_6.

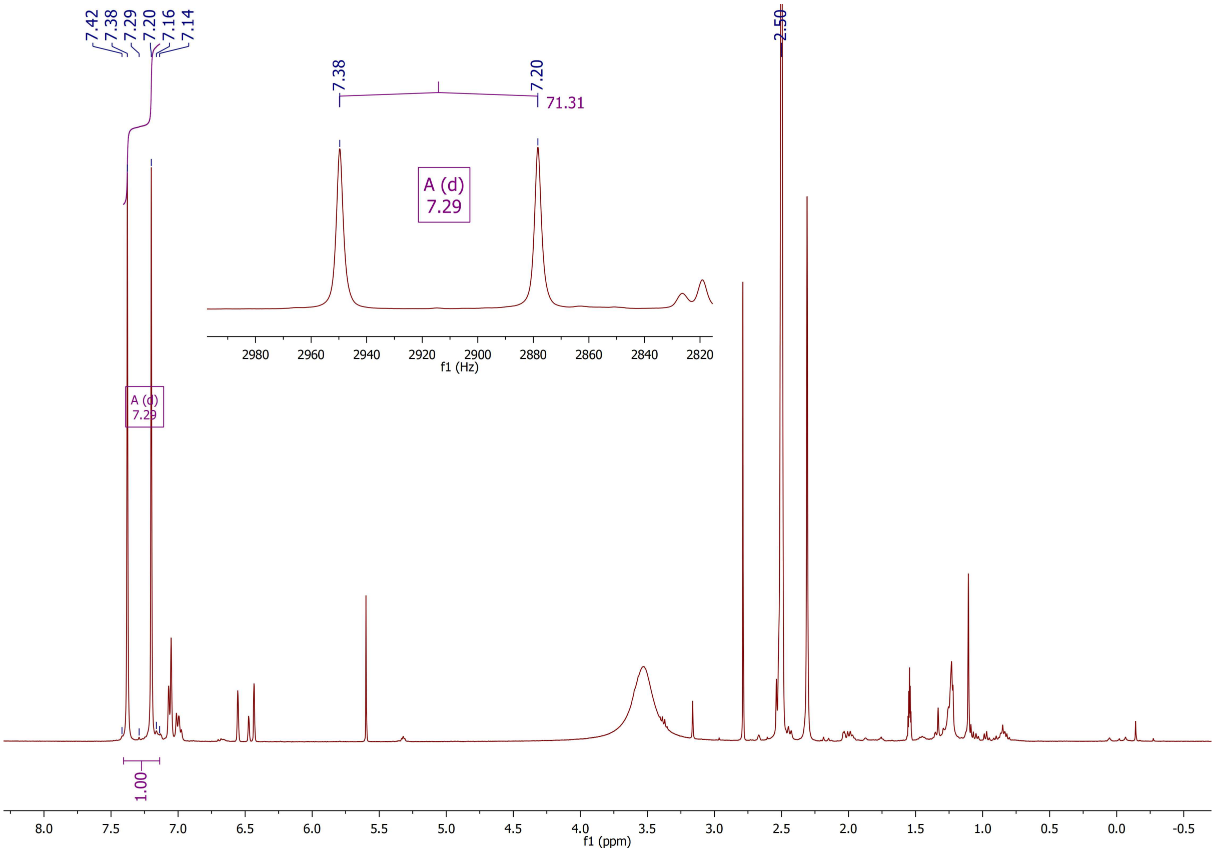

Figure S7. ^{1}H NMR spectrum of $^{15}NH_4Cl$ from $^{15}N_2$ reduction experiment of **3** in DMSO-d_6.

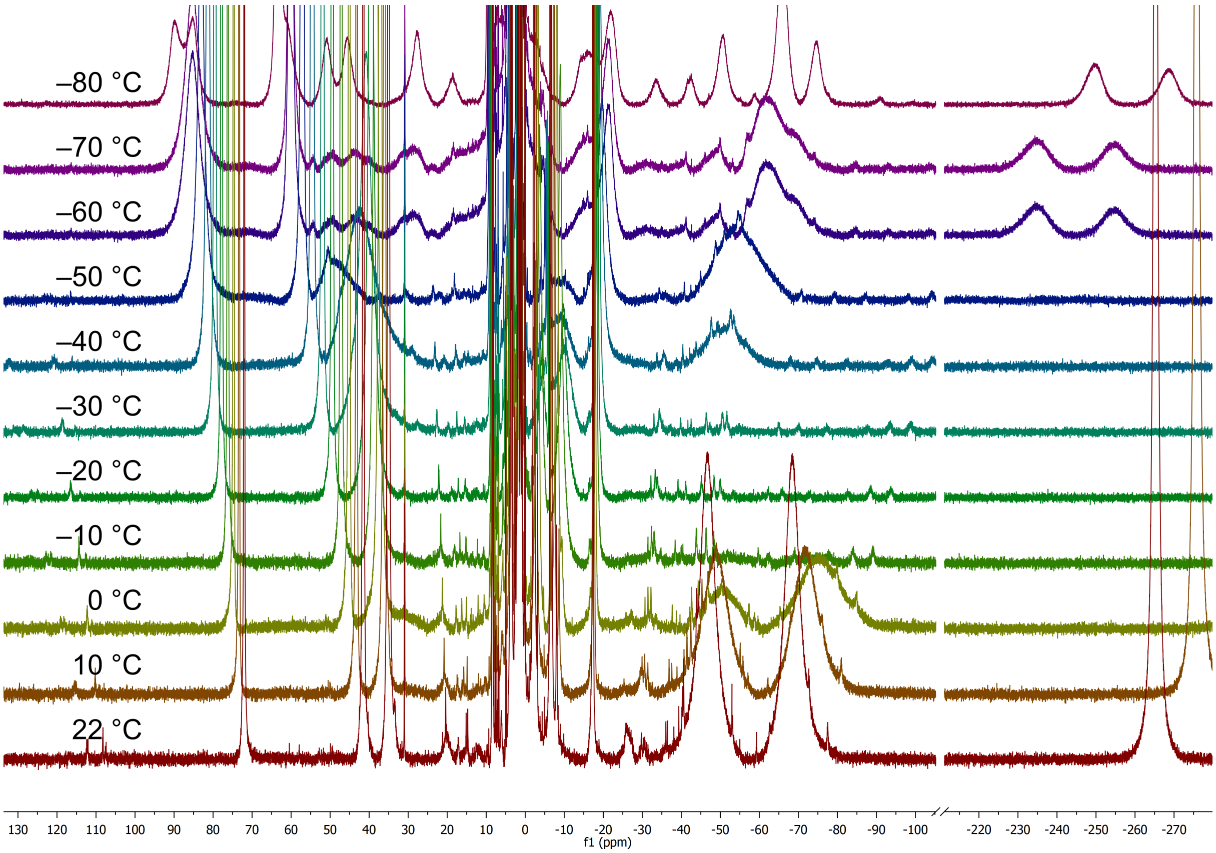

Figure S8. Stacked ^{1}H NMR overlay of **1** in THF-d_8 under N_2 from 22 °C to –80 °C

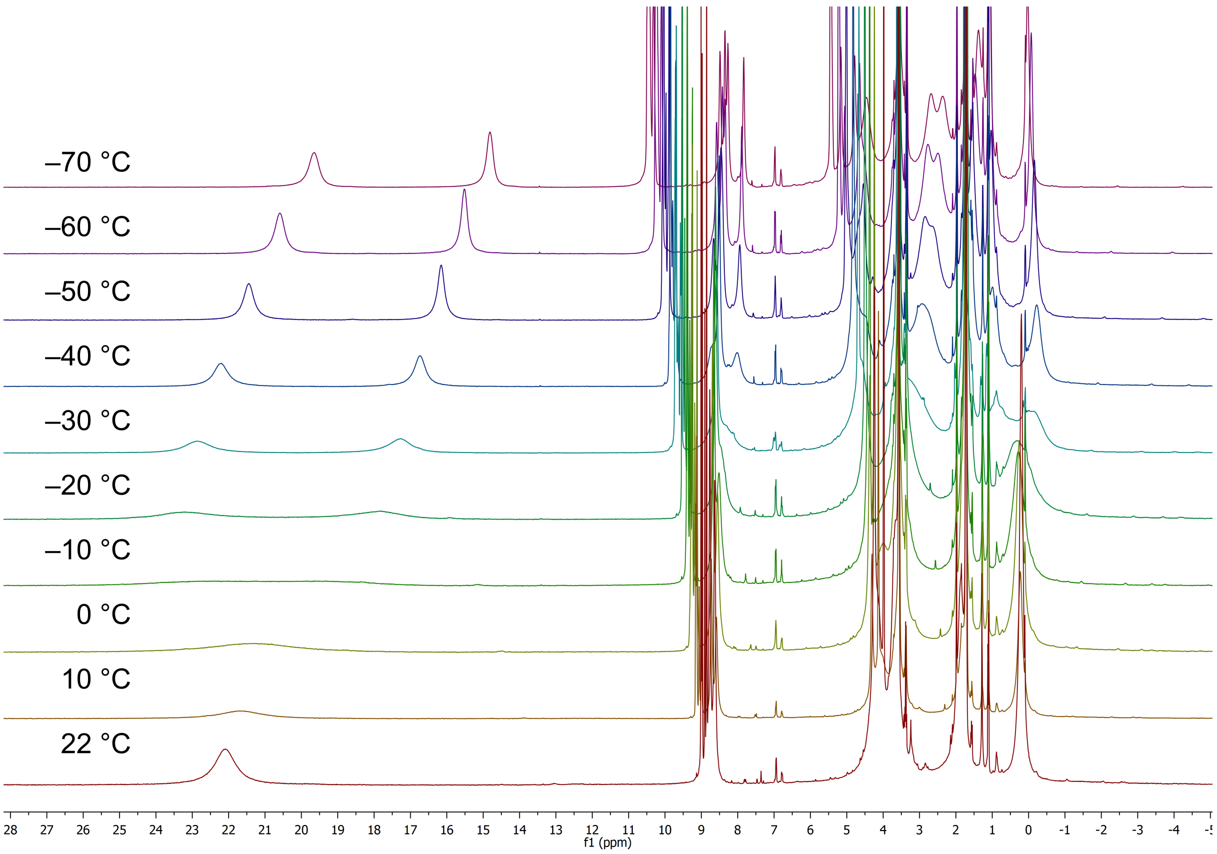

Figure S9. Stacked ^1H NMR overlay of **2** in THF-d_8 under N_2 from 22 °C to –70 °C

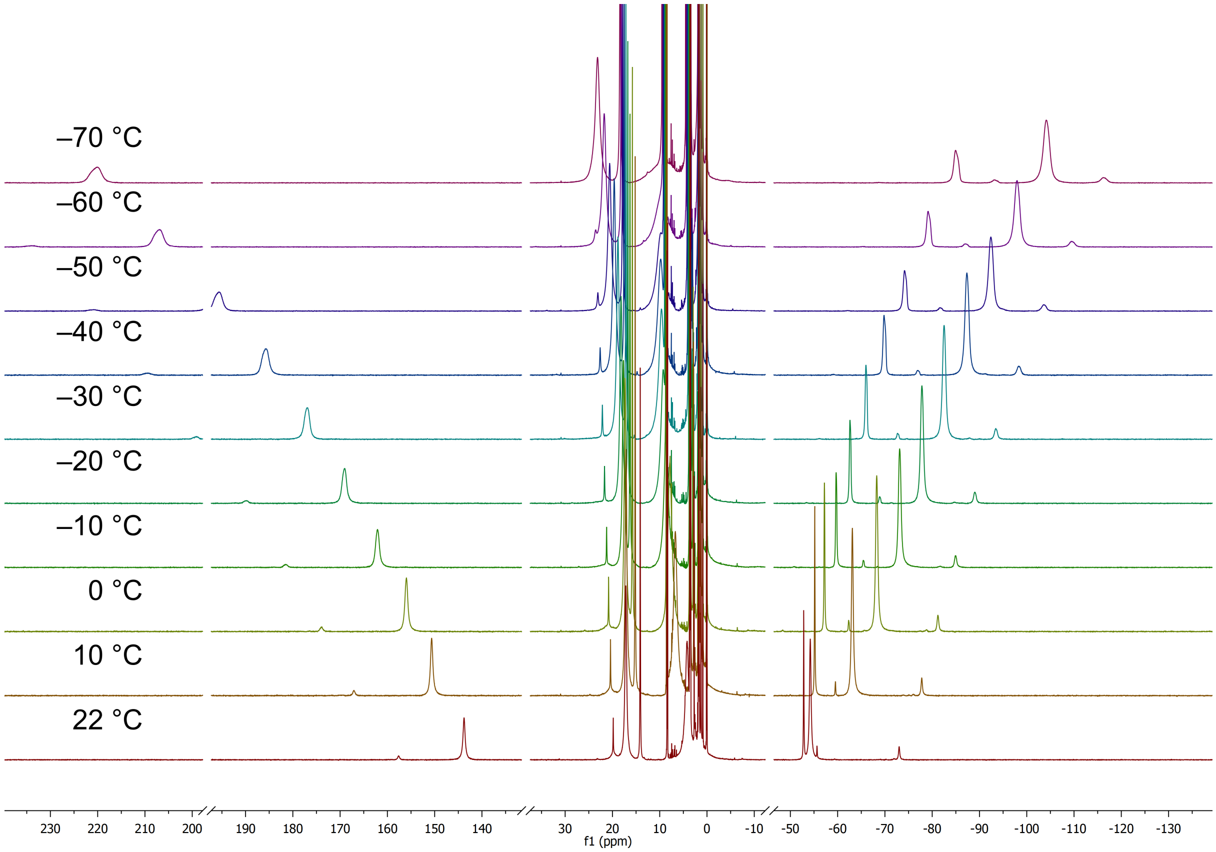

Figure S10. Stacked ^{1}H NMR spectra of **3** in THF-d_8 under N_2 from 22 °C to –70 °C

Van't Hoff Analysis

In Et_2O-d_{10}

Experimental Details: A solution of **4** (5.0 mg, 5.5 µmol) in Et_2O-d_{10} (0.5 mL) was transferred into a J. Young NMR tube with a headspace of N_2, and analyzed by ^1H NMR spectroscopy from –35 °C to –80 °C (Figure S11). The concentrations of **4** and **4–N$_2$** were determined by integration relative to a nickelocene capillary. K_{eq} was calculated according to Eq. 1. [N_2] in various solvents was calculated from the mole fraction solubility of N_2 in Et_2O as a function of temperature.[15] The reaction enthalpy ($\Delta H°$) and entropy ($\Delta S°$) were obtained from the slope and *y* intercept of the van't Hoff plot (Fig. S12) according to Eq. 2 and Eq. 3, respectively (*R* is the ideal gas constant).

4 + $N_2 \rightleftharpoons$ **4–N$_2$**

Eq. 1 $$K_{eq} = \frac{[\mathbf{4}-\boldsymbol{N_2}]}{[\mathbf{4}][N_2]}$$

Eq. 2 $$Slope = -\Delta H°/R$$

Eq. 3 $$Y - intercept = \Delta S°/R$$

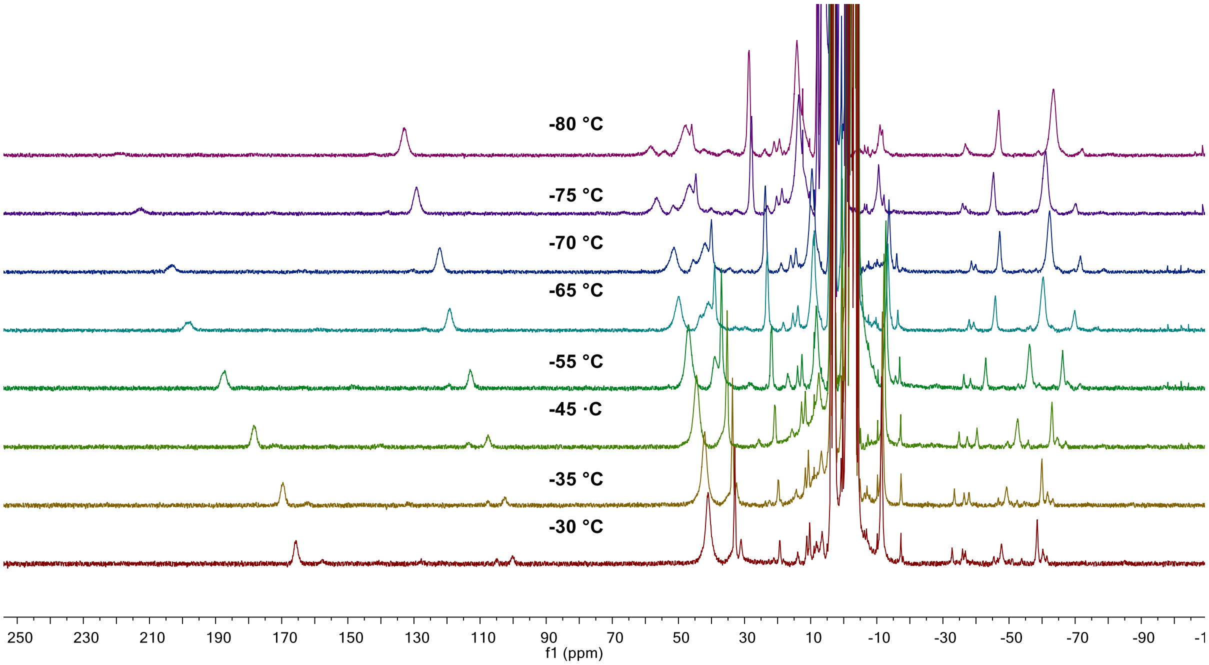

Figure S11. Stacked ^{1}H NMR spectra of **4** in Et_2O-d_{10} from –35 °C to –80 °C

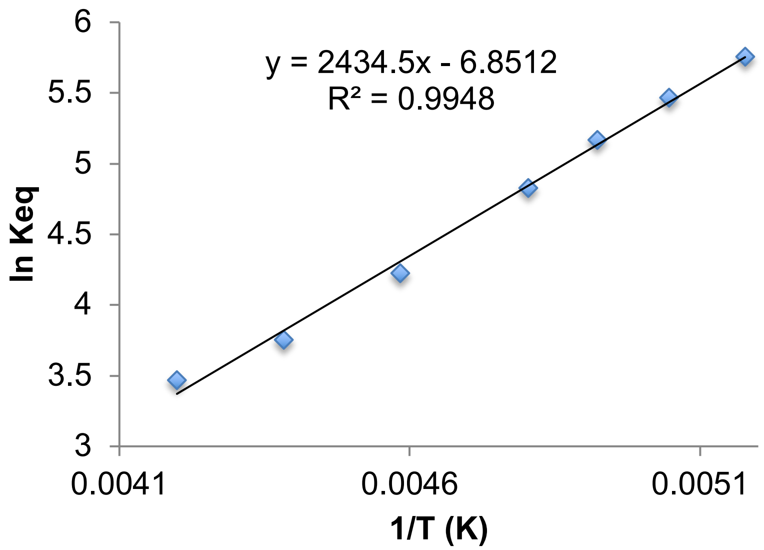

Temp (°C)	K_{eq} (M^{-1})
–80	316
–75	237
–70	175
–65	125
–55	68.3
–45	42.6
–35	32.1

Figure S12. van't Hoff plot for the equilibrium mixture of **4**, N_2 and **4–N₂** in Et_2O-d_{10} (left) and the K_{eq} values (M^{-1}) at various temperatures (right).

Van't Hoff Analysis in THF-d_8

Experimental Details: A solution of **4** (5.7 mg, 5.5 μmol) in THF-d_8 (0.5 mL) was transferred into a J. Young NMR tube, and analyzed by ^{1}H NMR spectroscopy at from 22°C to –80 °C (Figure S13). The concentrations of **4** and **4–N$_2$** were determined by integration relative to a nickelocene capillary. K_{eq} was calculated according to eq. 1. [N$_2$] in various solvents was calculated from the mole fraction solubility of N_2 in THF as a function of temperature.[15] We were unable to find literature measurements of the N_2 solubility in THF below room temperature, so we assumed that the temperature dependence of N_2 solubility in THF varied as in Et_2O, but adjusting the room-temperature value to that reported.[15] The reaction enthalpy ($\Delta H°$) and entropy ($\Delta S°$) were obtained from the slope and *y* intercept of the van't Hoff plot (Fig. S14) according to Eq. 2 and Eq. 3, respectively (*R* is the ideal gas constant).

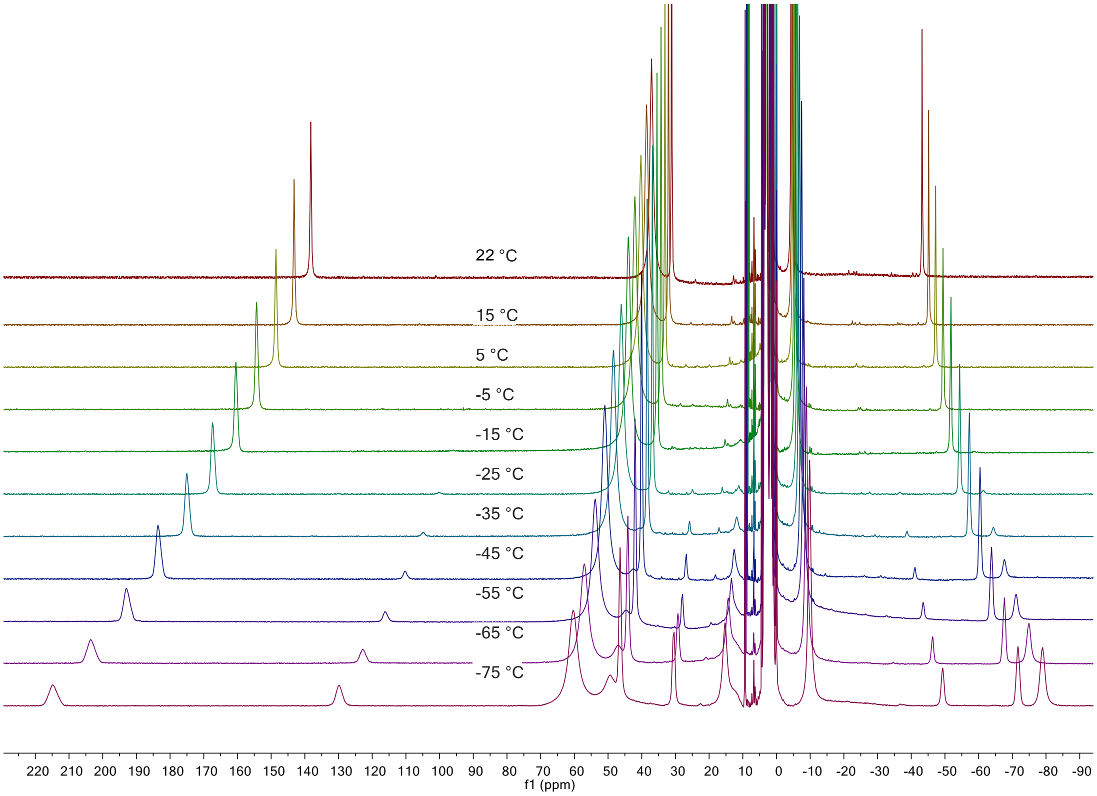

Figure S13. Stacked ^{1}H NMR spectra of **4** in THF-d_8 under N_2 from 22 °C to −75 °C

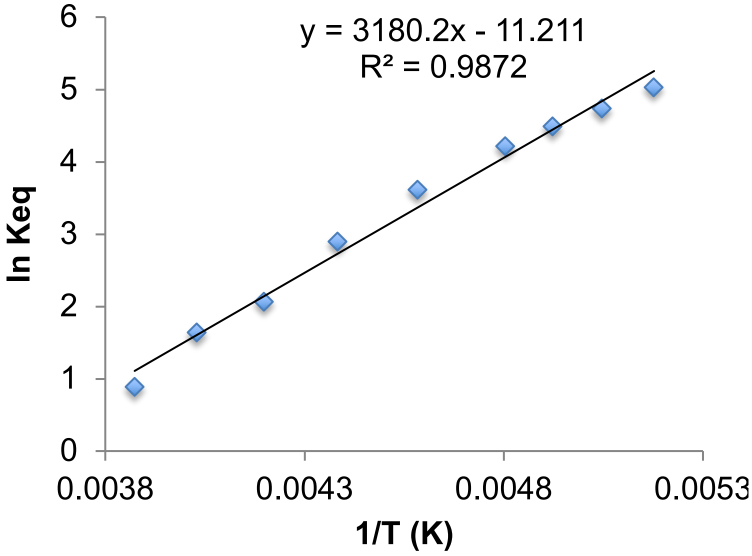

Temp (°C)	K_{eq} (M^{-1})
–80	152
–75	114
–70	88.5
–65	67.7
–55	36.9
–45	18.1
–35	7.86
–25	5.14
–15	2.43

Figure S14. van't Hoff plot for the equilibrium mixture of **4**, N_2 and **4–N$_2$** in THF-d_8 (left) and the K_{eq} values (M^{-1}) at various temperatures (right).

Table S2 Comparison of Thermodynamic Parameters of N_2 Binding to Other Published Complexes[10, 16, 17]

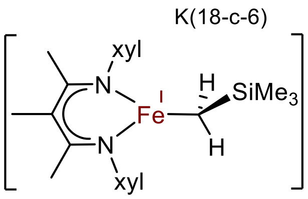

4

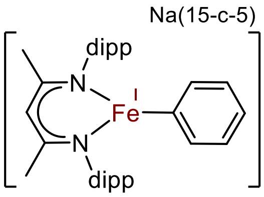

McWilliams *et al.*

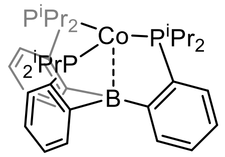

Suess *et al.*

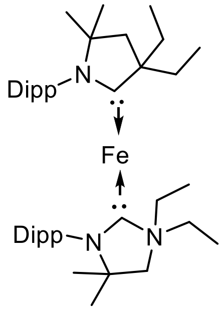

Ung *et al.*

	**4**		$[L^{Me,iPr}FePh][Na(15\text{-}c\text{-}5)]$	$Co(BP^{iPr}_3)$	$Fe(CAAC)_2$
Solvent	THF	Et_2O	THF	Toluene	Pentane
$\Delta H°$ (kJ mol^{-1})	–26 ± 1	–20 ± 1	–17 ± 2	–58 ± 3	–92.5*
$\Delta S°$(J mol^{-1} K^{-1})	–93 ± 5	–57 ± 3	–55 ± 10	–134 ± 21	–34*

* No error bars reported

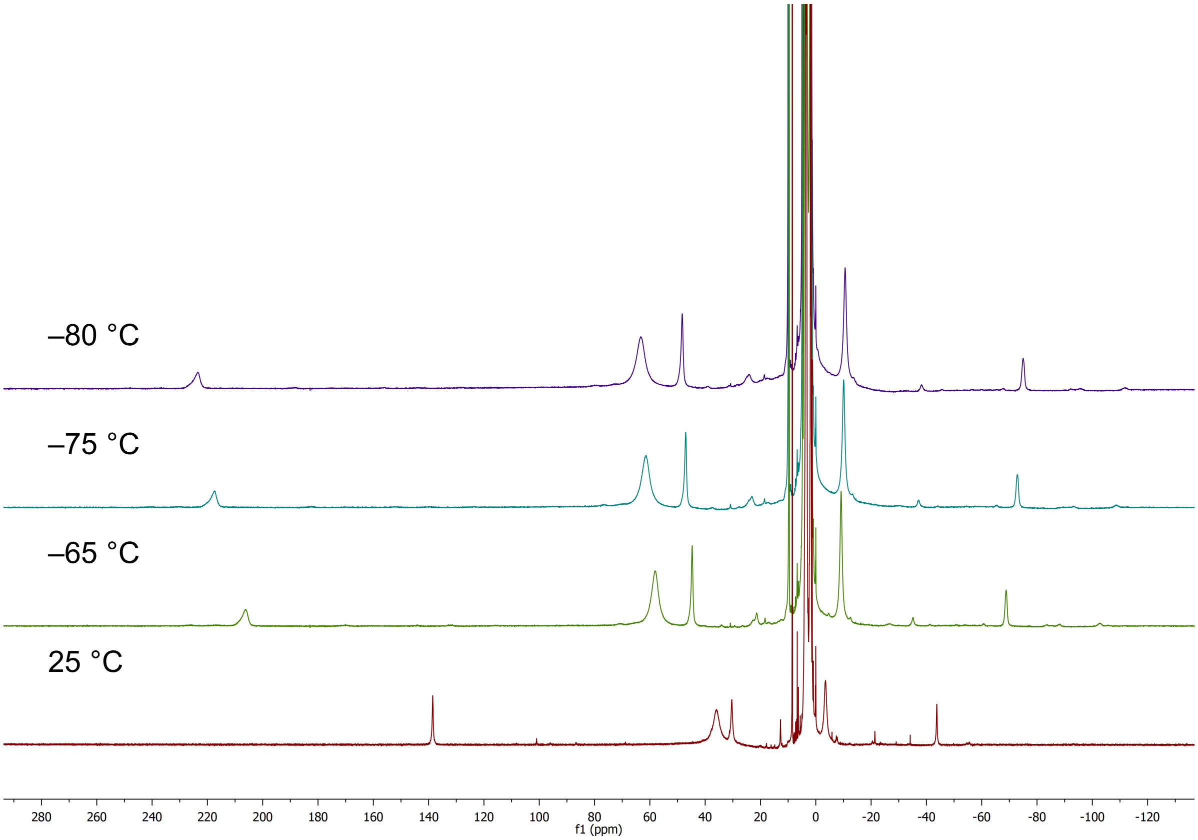

Figure S15. Stacked ^{1}H NMR spectra of **4** in THF-d_8 under Ar at various temperatures.

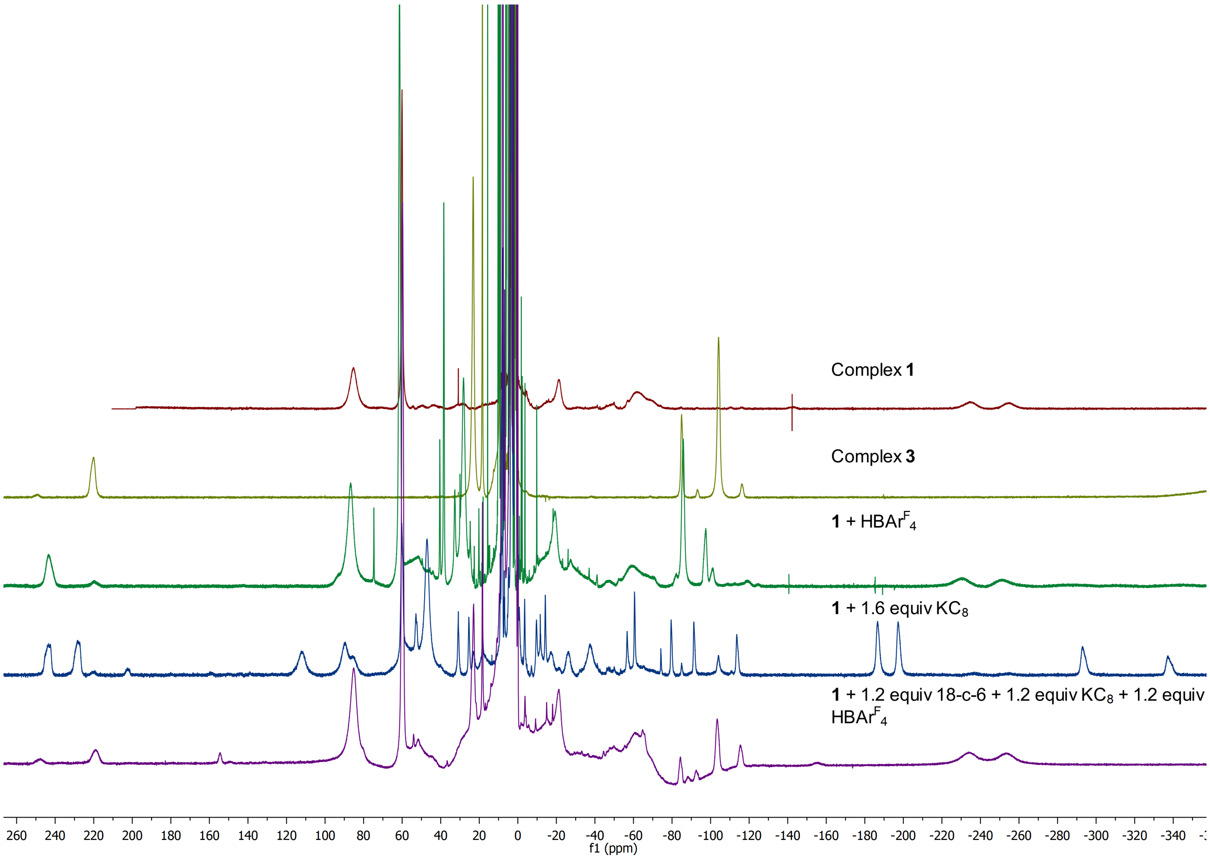

Figure S16. Stacked ^{1}H NMR spectra of various reactions of **1** in THF-d_8 under N_2 at –70 °C.

UV-Vis Spectra

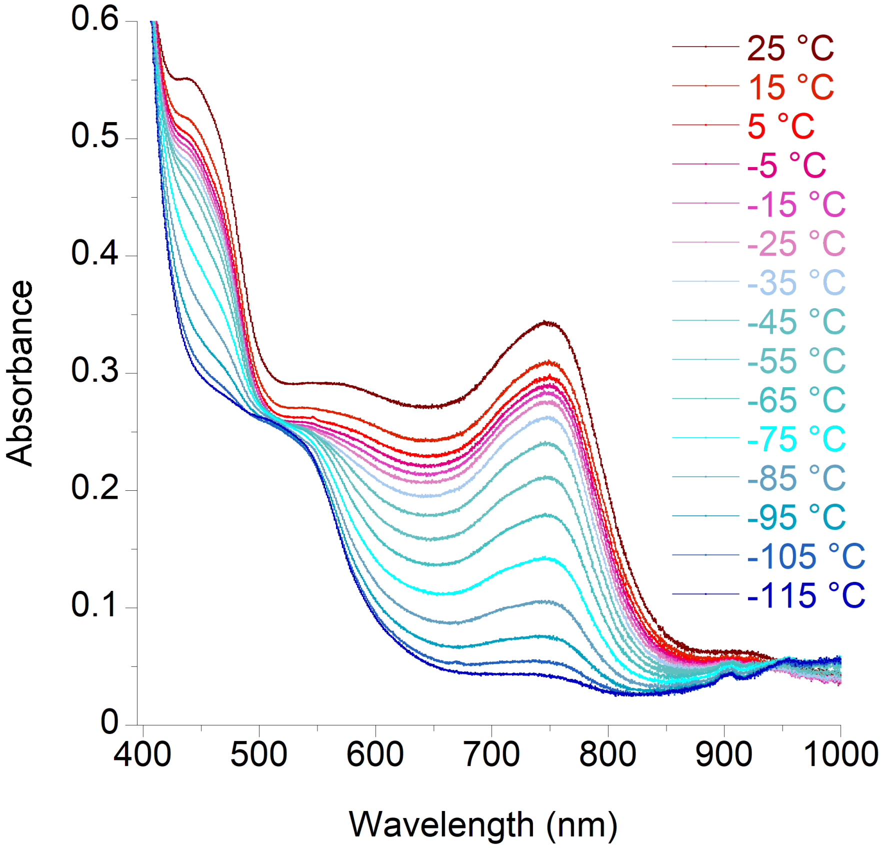

Figure S17. UV-visible spectra of **4** in Et_2O under N_2 at various temperatures. The changes observed under Ar are much less (see Fig. S18). This is a parallel experiment to the one included in Fig. 3a in the paper to show reproducibility and the sensitivity of the dilute solution of **4** to thermal decomposition. For this reason, the thermodynamic parameters of N_2 binding were determined via ^1H NMR spectroscopy measured at various temperatures.

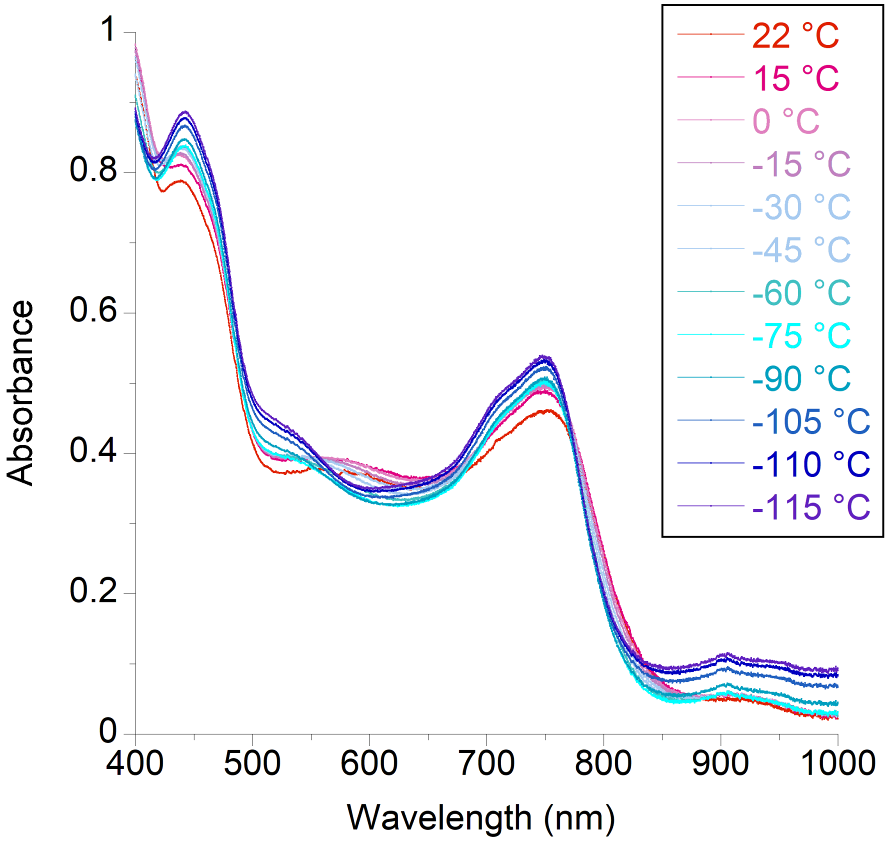

Figure S18. UV-visible spectra of **4** in Et_2O under Ar at various temperatures. The UV-Vis features change much less than the parallel experiment under N_2 (Fig. 3, Fig. S17).

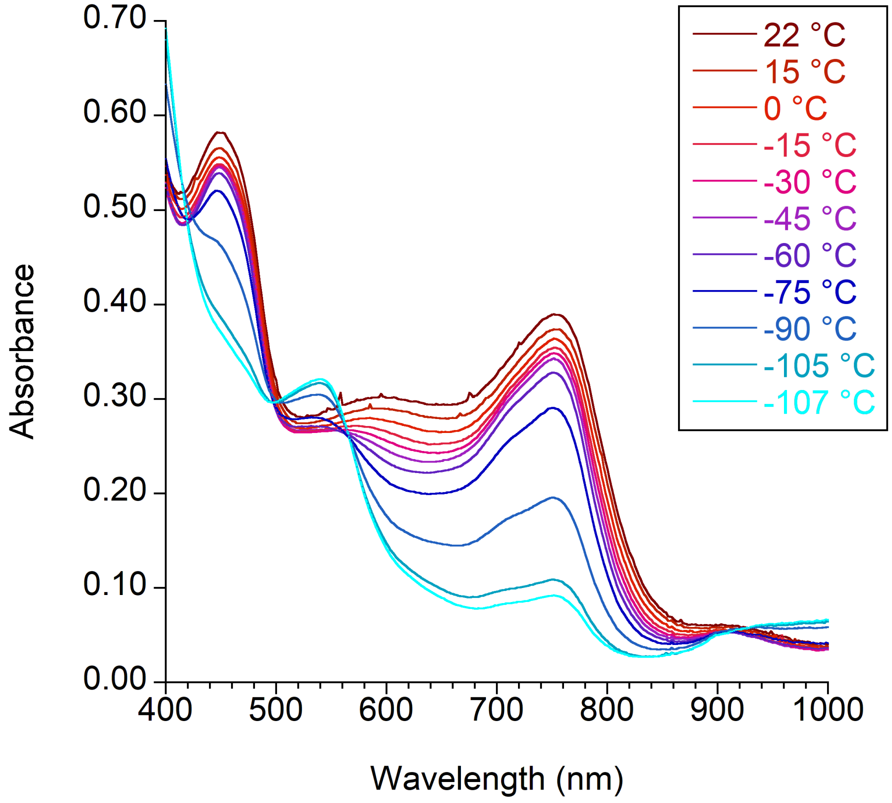

Figure S19. UV-visible spectra of **4** in THF under N_2 at various temperatures.

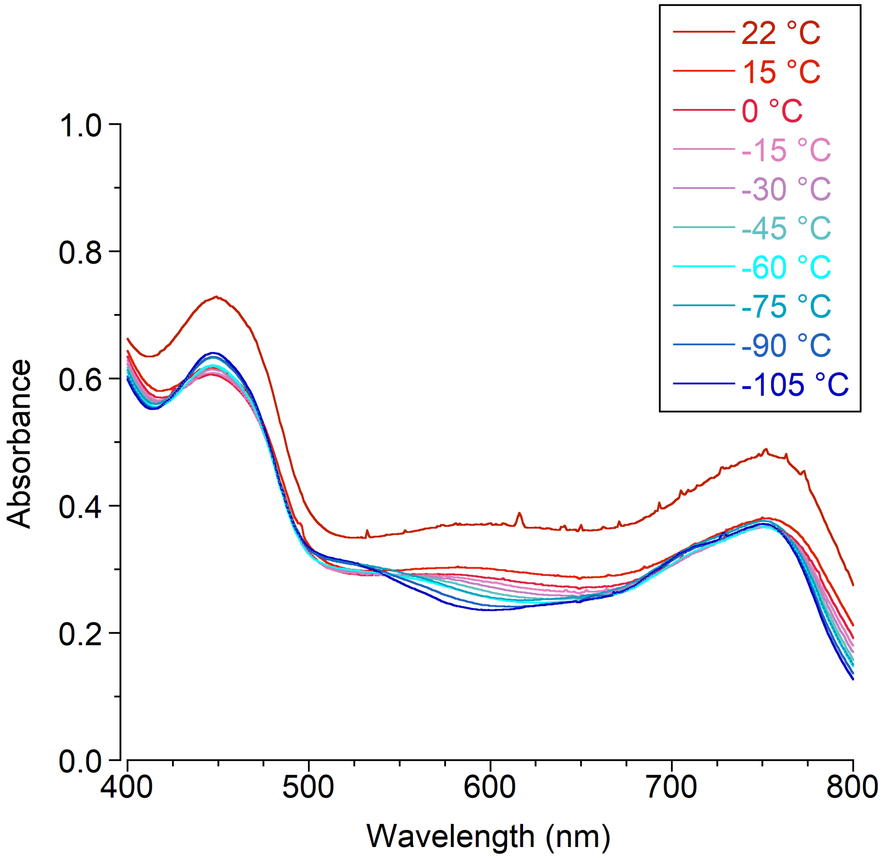

Figure S20. UV-visible spectra of **4** in THF under Ar at various temperatures. The UV-Vis features change much less than the parallel experiment under N_2 (Fig. S19).

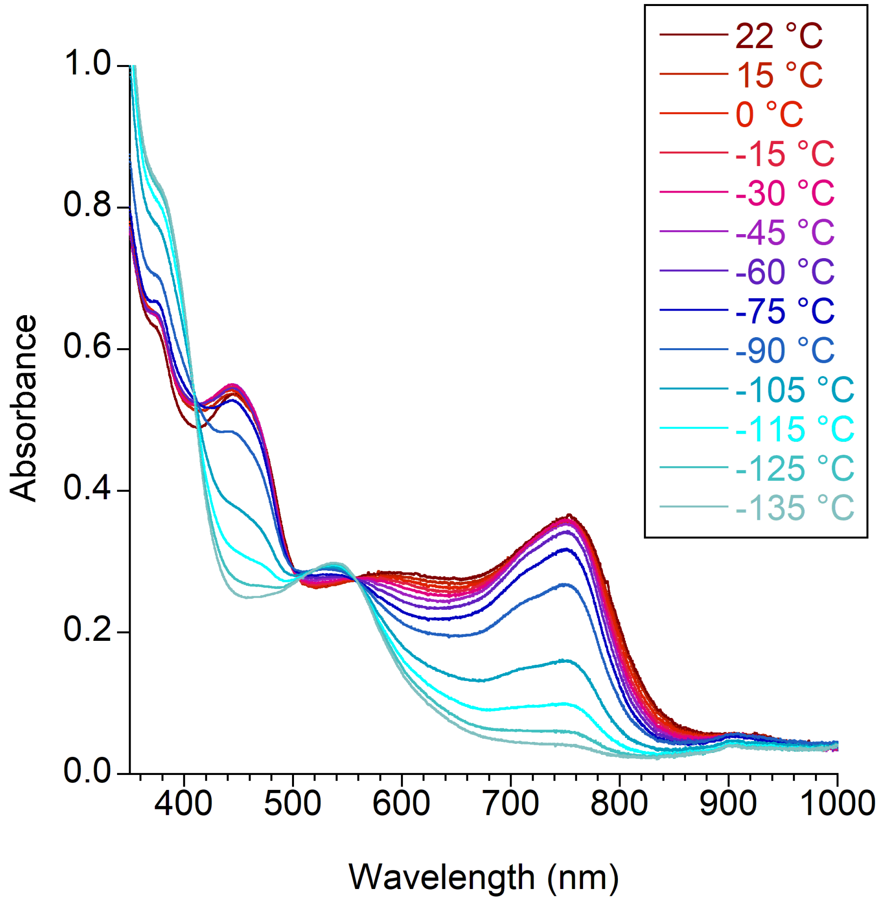

Figure S21. UV-visible spectra of **4** in MeTHF under N_2 at various temperatures.

Mössbauer Spectra

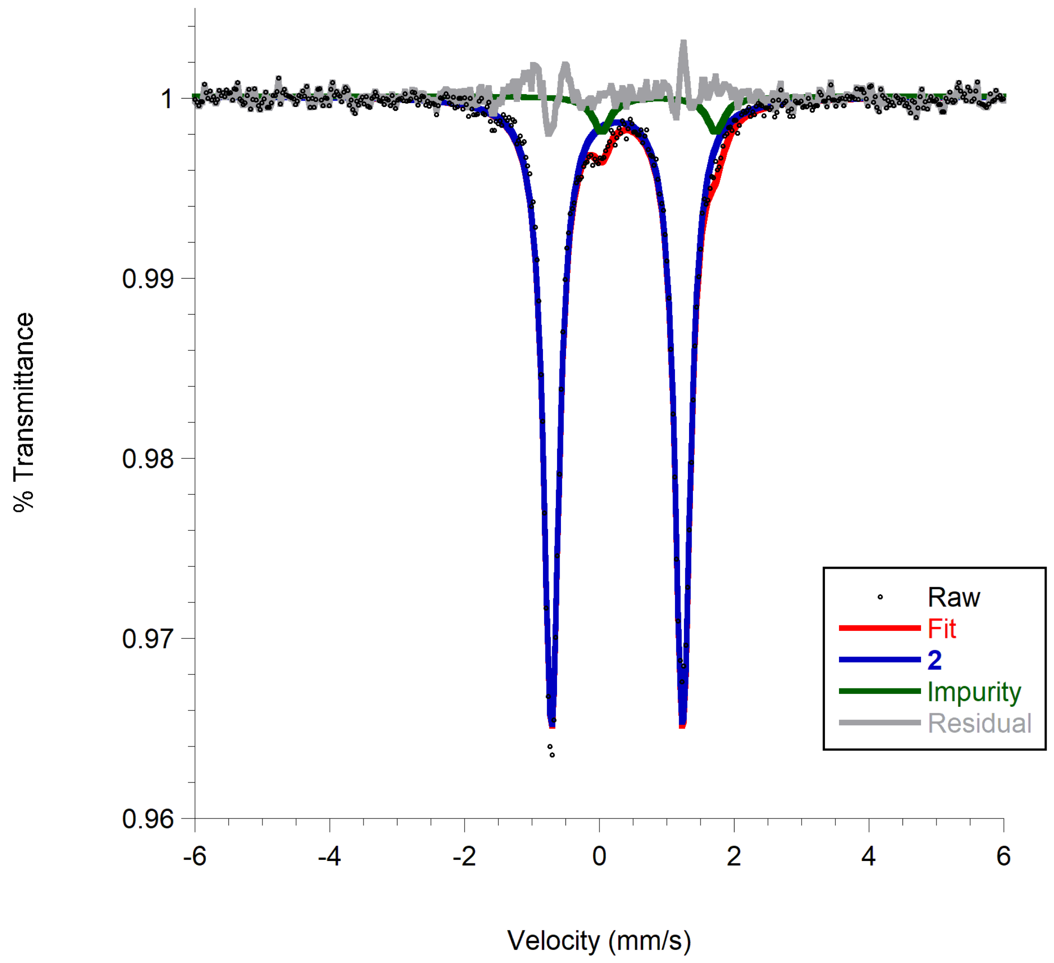

Figure S22. Zero-field Mössbauer spectrum of solid **2** recorded at 80 K. This figure shows the fit where the black circles represent the data, the red line is the two-component simulation, the blue line is **2** with the parameters $\delta = 0.26$ mm s^{-1}, $|\Delta E_Q| = 1.95$ mm s^{-1}, (94%), the green line is an impurity with the parameters $\delta = 0.88$ mm s^{-1}, $|\Delta E_Q| = 1.69$ mm s^{-1}, (6%), and the gray line is the residual.

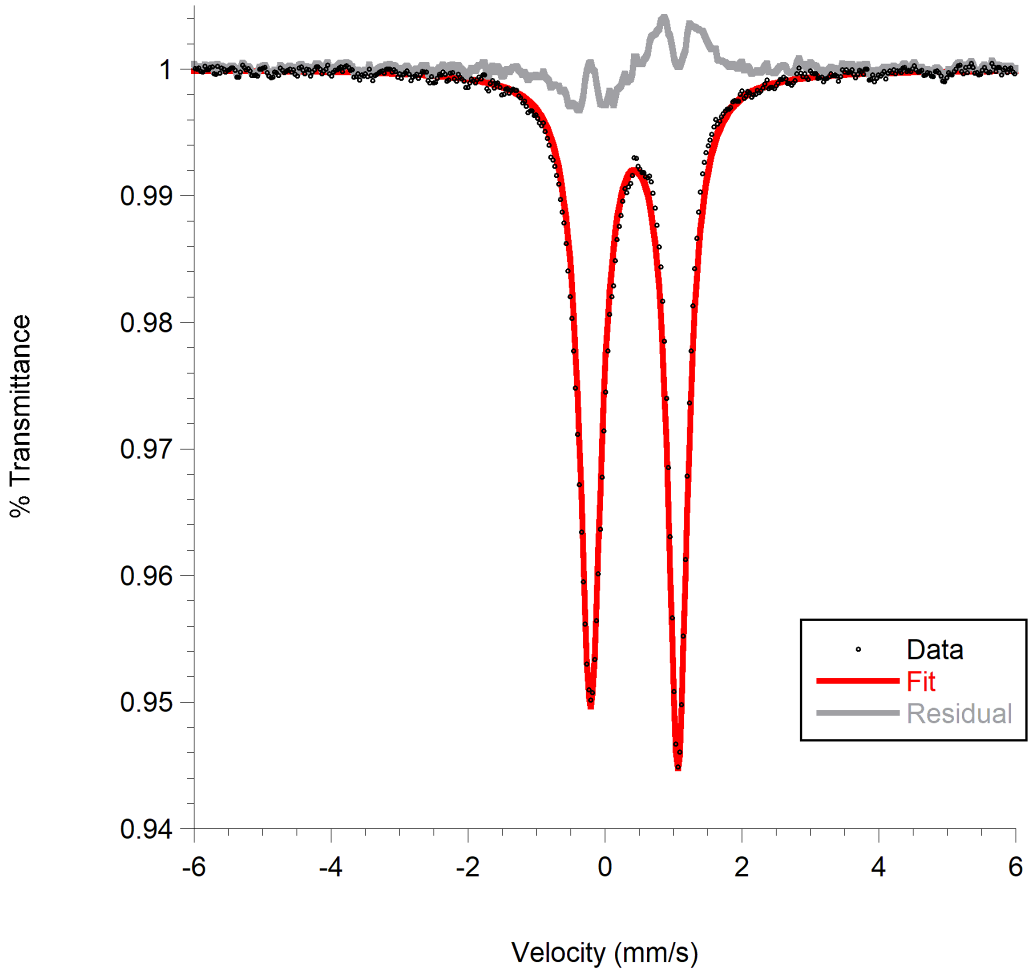

Figure S23. Zero-field Mössbauer spectrum of solid **3** recorded at 80 K. This figure shows the fit where the black circles represent the data, the red line is the one-component simulation with parameters δ = 0.43 mm s^{-1}, $|\Delta E_Q|$ = 1.28 mm s^{-1}, Γ_L = 0.39, Γ_R = 0.35, and the gray line is the residual.

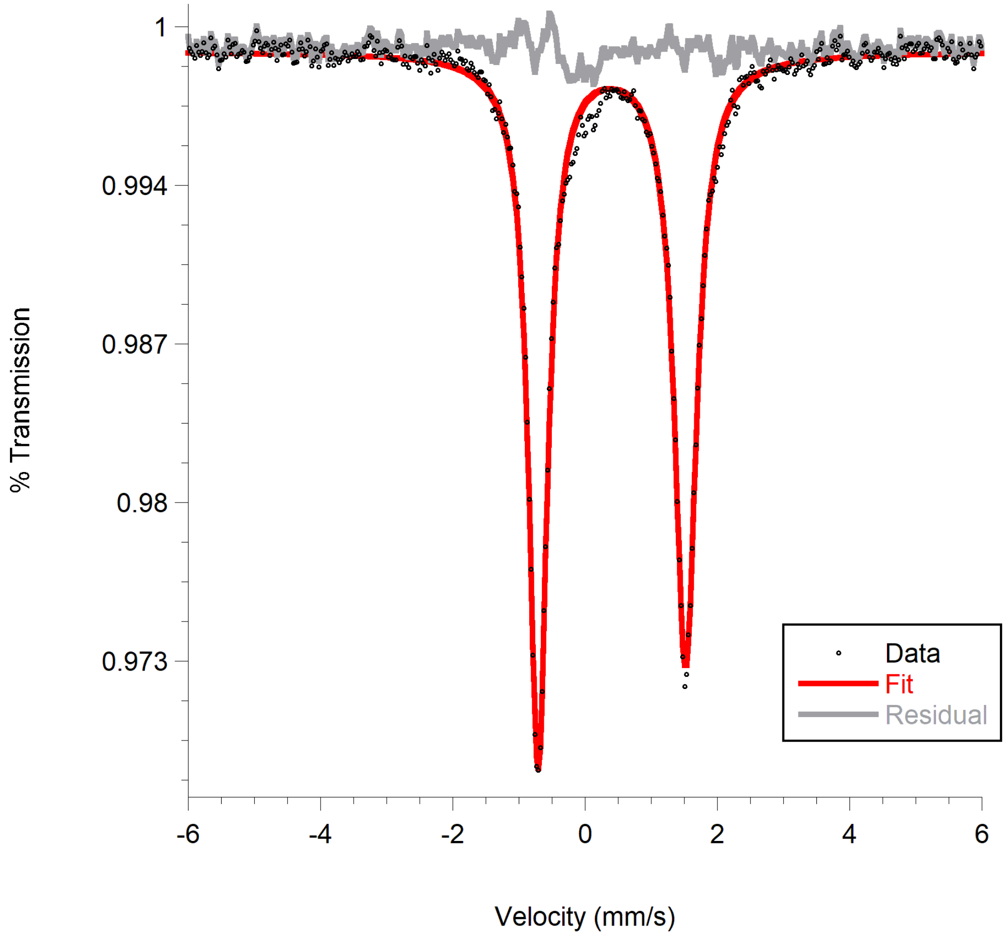

Figure S24. Zero-field Mössbauer spectrum of solid **4** recorded at 80 K. This figure shows the fit where the black circles represent the data, the red line is the one-component simulation with parameters δ = 0.41 mm s^{-1}, $|\Delta E_Q|$ = 2.23 mm s^{-1}, Γ_L = 0.35, Γ_R = 0.41, and the gray line is the residual.

IR Spectra

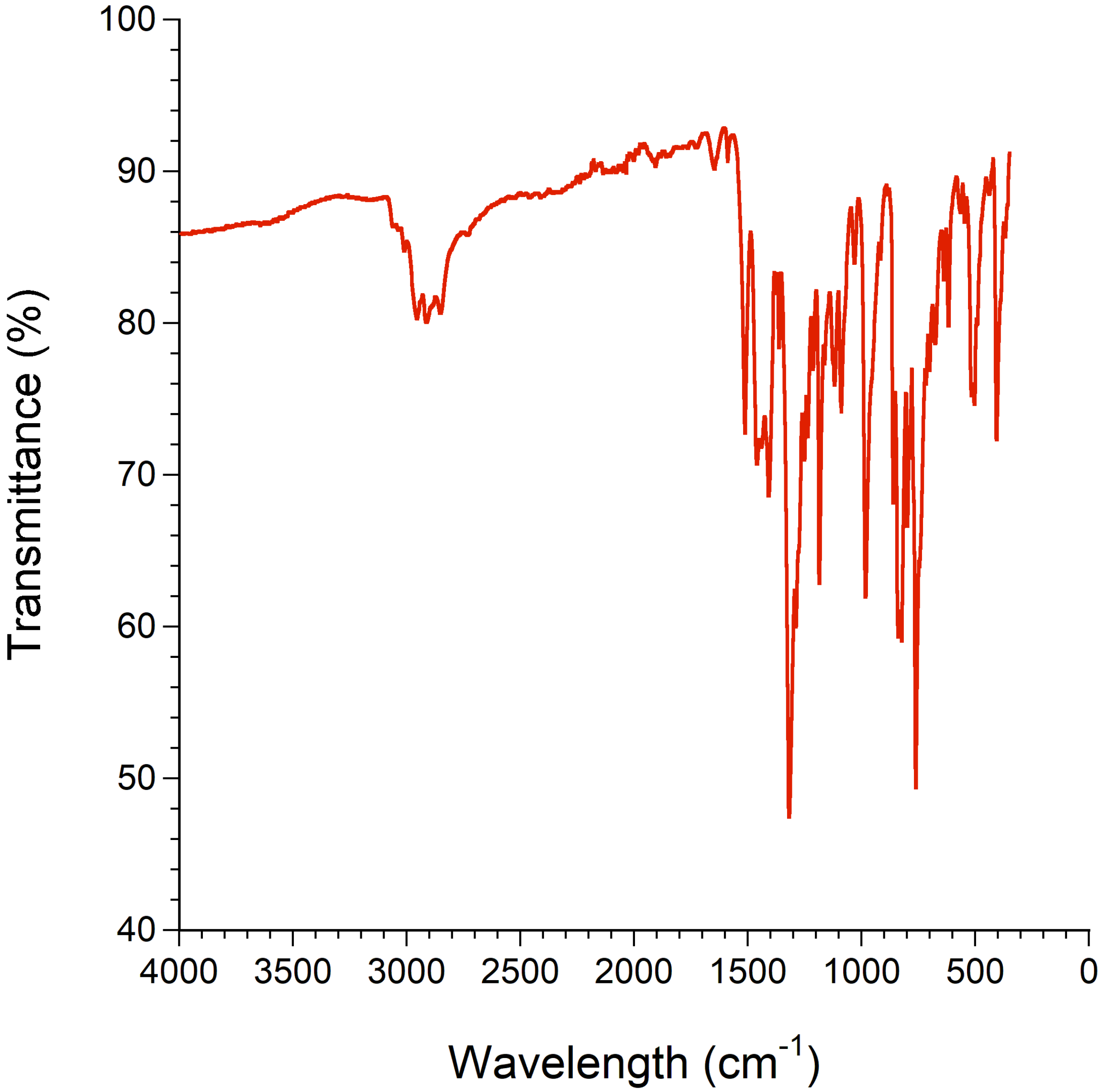

Figure S25. IR spectrum of solid **2**.

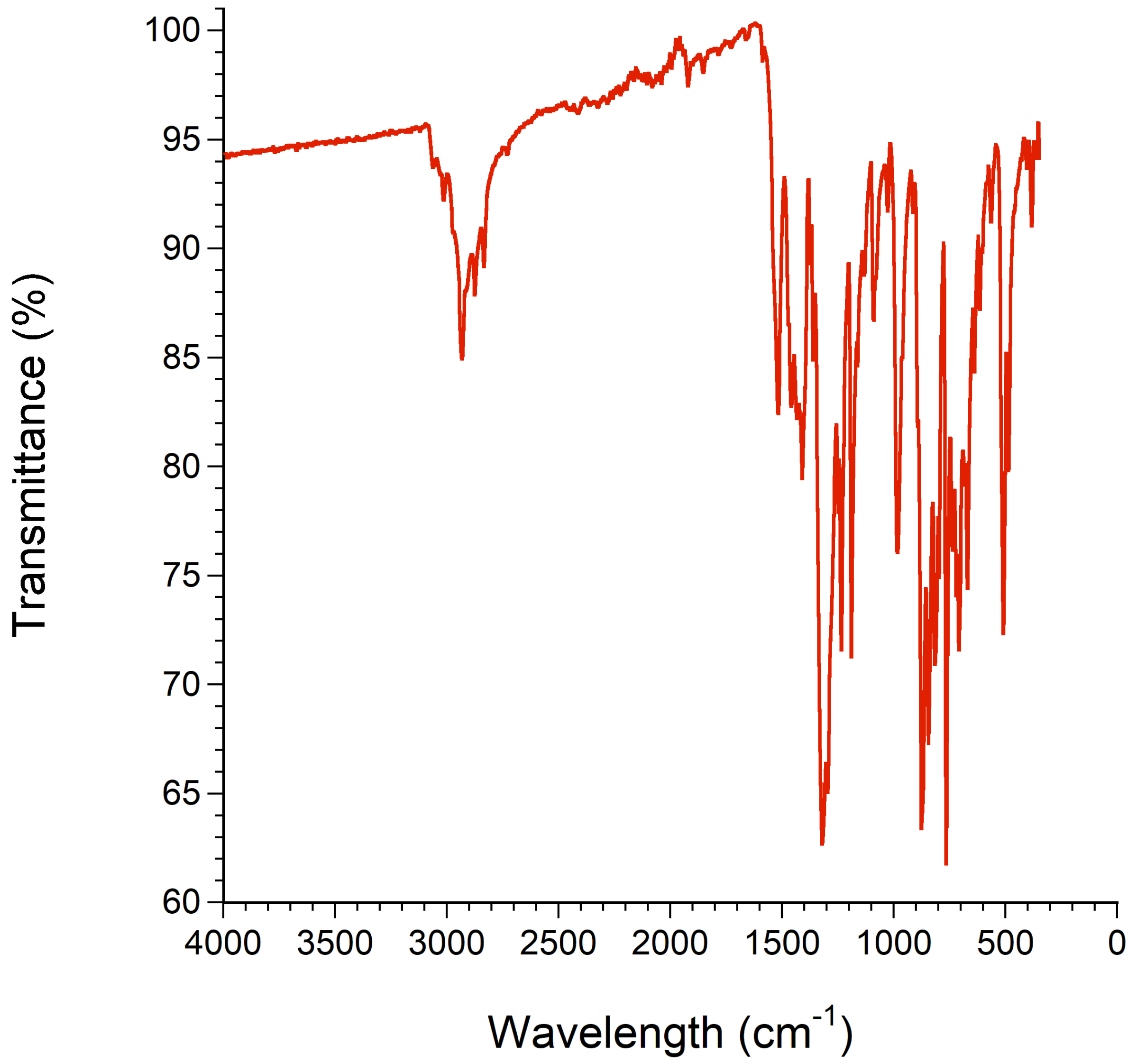

Figure S26. IR spectrum of solid **3**.

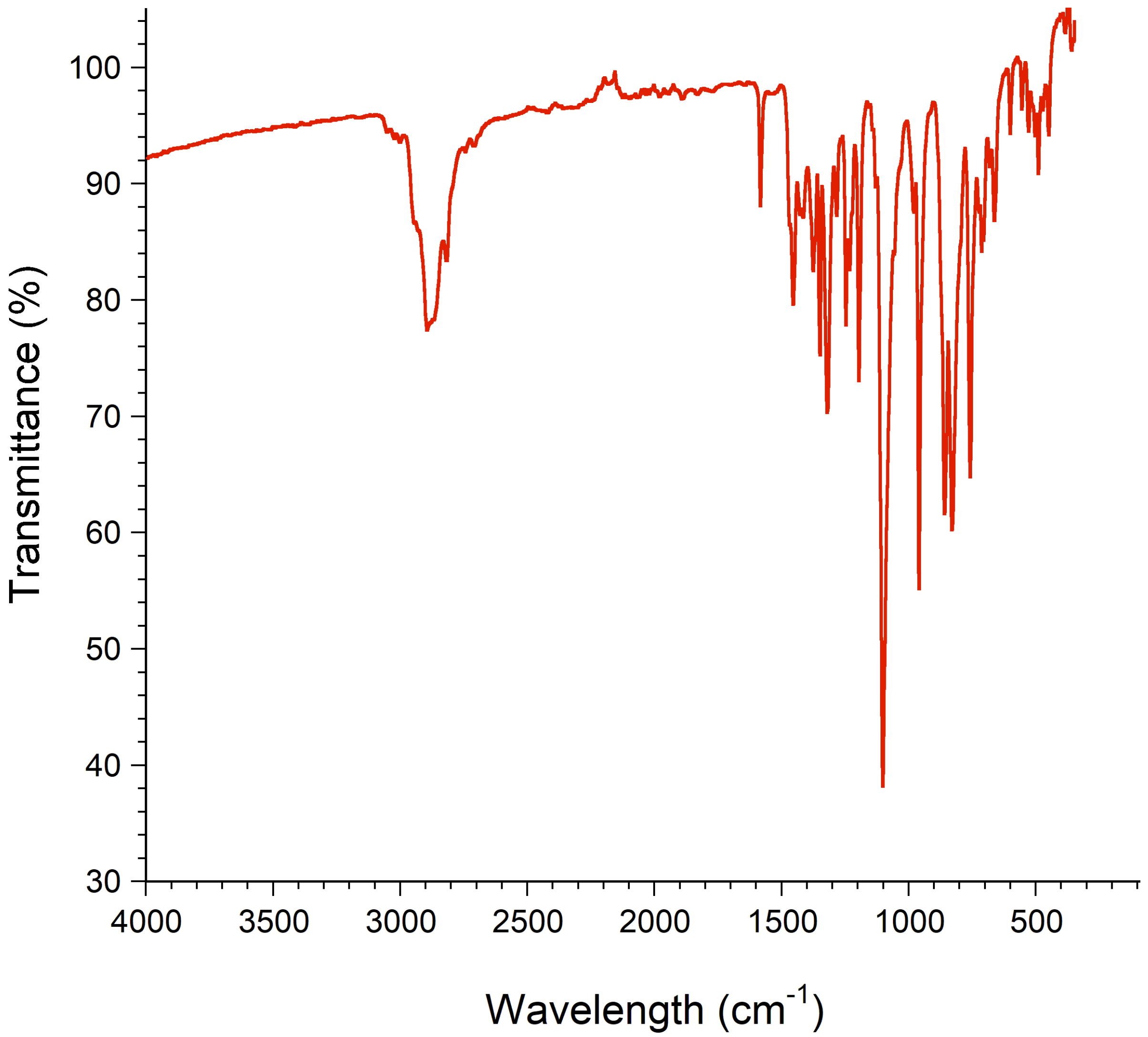

Figure S27. IR spectrum of solid **4**.

Magnetic measurements. Magnetic data were collected using a Quantum Design MPMS 3 superconducting quantum interference device (SQUID) magnetometer. Magnetic measurements for all compounds were performed on ground microcrystalline solids. The samples were prepared under an atmosphere of N_2, restrained with Krytox, and flame sealed in quartz tubes under vacuum. Dc magnetic measurements were collected in the temperature range of 2–225 K. Data collected above 225 K were unreliable owing to the melting of Krytox that resulted in movement of the sample. Variable field magnetization curves were collected at 100 K to check for curvature associated with the presence of ferromagnetic impurities. Dc magnetic susceptibility measurements were performed under applied magnetic fields of 0.05 T and 0.10 T, and corrected for the diamagnetism of each sample and Krytox, estimated using Pascal's constants.[18] Variable field, variable temperature magnetization measurements (reduced magnetization) were performed under applied magnetic fields of 1, 2, 3, 4, 5, 6 and 7 T in the temperature range of 2–10 K. Dc magnetic susceptibility data were simulated using the program PHI[19] and reduced magnetization data were simulated in the program DAVE 2.0.[20] The spin Hamiltonians employed accounted for g-anisotropy, axial and transverse zero-field splitting (D and E) and exchange interactions for compounds **1** and **2**. Reduced magnetization measurements were not performed on the dinuclear iron complexes given their antiferromagnetic coupling resulting in $S = 0$ ground states. Simulation of the Dc magnetic susceptibility for **1** was performed with the spin Hamiltonian $\hat{H} = \sum_i^{1,2} D\hat{S}_{iz}^2 + E(\hat{S}_{ix}^2 - \hat{S}_{iy}^2) + g_i \mu_B \boldsymbol{S}_i \boldsymbol{H} - 2J\boldsymbol{S}_1\boldsymbol{S}_2$. The subscripts 1 and 2 refer to each Fe^{2+} center. To avoid overparameterization of the simulation, we treated both Fe^{2+} centers as magnetically identical, and used values of D, E, and g_{iso} values determined from simulation of magnetic data for **3**. Simulation of the dc magnetic susceptibility data for **2** used the same Hamiltonian describe for **1**, and we treated both Fe^{3+} centers as magnetically identical. For simulation of the data for **2**, we restrained the value of D to < 5 cm^{-1}, the isotropic g-value to the range of 1.95 to 2.1, and E to be zero.[21] Modelling the data with larger values of D had little impact on the curvature of the simulation, as it is dominated by strong antiferromagnetic exchange coupling. Additionally, simulation of the dc magnetic susceptibility data for **1** and **2** accounted for paramagnetic impurities that were assumed to be mononuclear high spin Fe^{3+} species. Simulation of **1** included a 2.1% paramagnetic impurity, while the data for **2** was simulated with a 10.7% paramagnetic impurity to account for the non-zero $\chi_M T$ value at low temperature.

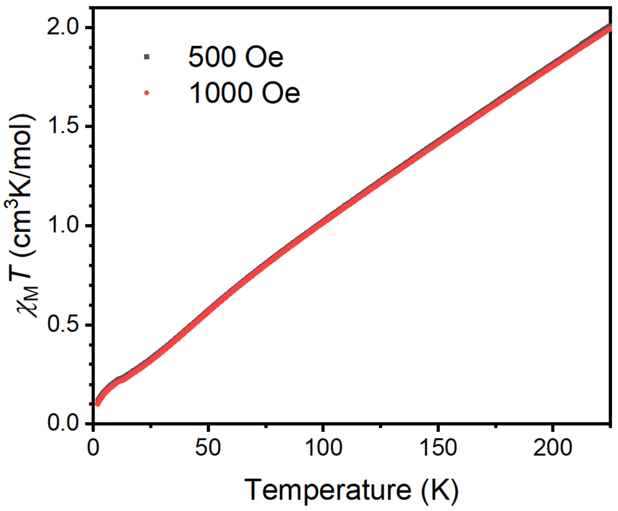

Figure S28. Dc magnetic susceptibility data for **1** collected under applied magnetic fields of 500 (black) and 1000 Oe (red).

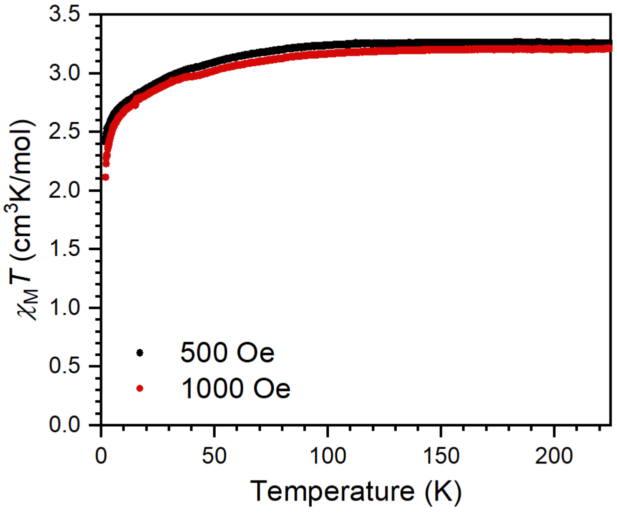

Figure S29. Dc magnetic susceptibility data for **3** collected under applied magnetic fields of 500 (black) and 1000 Oe (red).

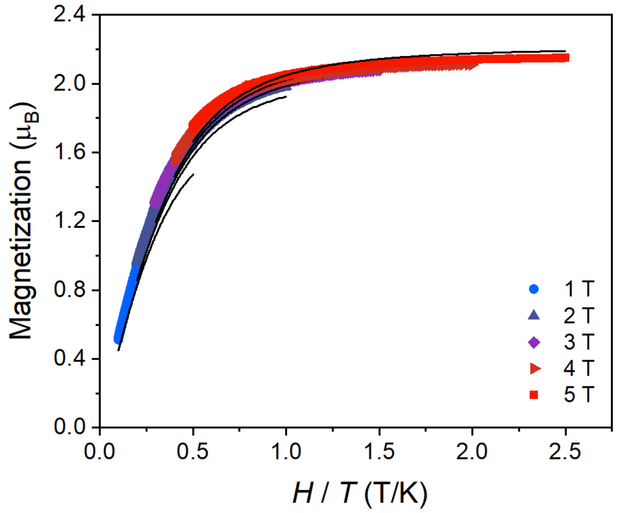

Figure S30. Variable-temperature, variable-field magnetization data for **3.** The data were collected between 2 and 10 K, and from 1 to 5 T in 1 T increments. Black lines are simulations of the data obtained using DAVE 2.0 and the spin Hamiltonian, $\hat{H} = D\hat{S}_z^2 + E(\hat{S}_x^2 - \hat{S}_y^2) + (g_\perp + g_{||})\mu_B \boldsymbol{SH}$. The parameters used to obtain the simulation are $D = -43$ cm^{-1}, $|E| = 5$ cm^{-1}, $g_\perp = 1.88$ and $g_{||} = 2.31$ ($g_{iso} = 2.03$). No satisfactory simulation could be obtained with positive D values.

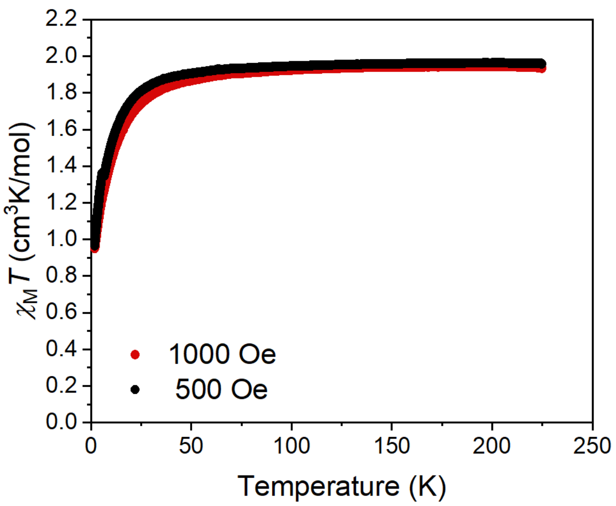

Figure S31. Dc magnetic susceptibility data for **4** collected under applied magnetic fields of 500 (black) and 1000 Oe (red).

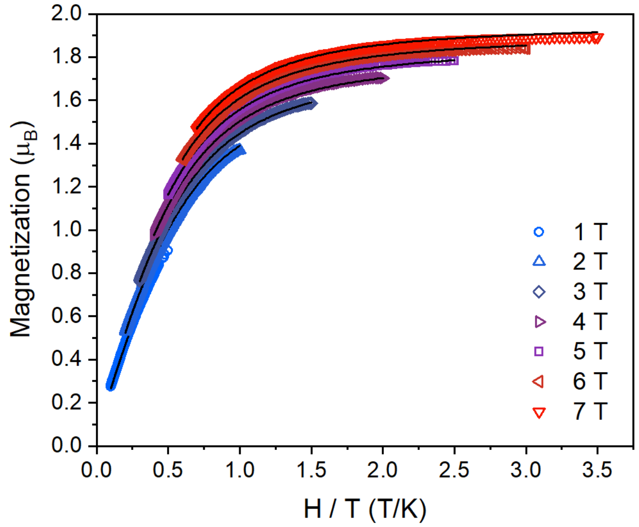

Figure S32. Variable-temperature, variable-field magnetization data for **4.** The data were collected between 2 and 10 K, and from 1 to 7 T in 1 T increments. Black lines are simulations of the data obtained using DAVE 2.0 and the spin Hamiltonian, $\hat{H} = D\hat{S}_z^2 + E(\hat{S}_x^2 - \hat{S}_y^2) + g_{iso}\mu_B\boldsymbol{SH}$. The parameters used to obtain the simulation are $D = -14.9$ cm^{-1}, $|E| = 1.9$ cm^{-1} and $g_{iso} = 2.04$. No satisfactory simulation could be obtained with positive D values.

Computations

DFT calculations were used to yield the geometry optimized structures of **2**, **3**, and **4** in all reasonable spin states. These calculations were performed with the ORCA program package, version 4.2.1.[22, 23] Optimized geometries were computed using the B3LYP functional.[24-28] Atom-pairwise dispersion correction with the Becke-Johnson damping scheme (D3BJ),[29, 30] the scalar relativistic zero-order regular approximation (ZORA),[31, 32] and the scalar relativistically recontracted version of the Aldrichs triple-ζ basis set, def2-TZVP, was used on all atoms. In the geometry optimizations on **2** using the B3LYP functional, the iron and alkylidene C, S and N atoms used the def2-TZVP basis set and all other atoms were modeled with def2-SVP. Mössbauer parameters were calculated using the B3LYP functional with either all atoms modeled with the def2-TZVP basis set or a combination of the def2-TZVP for the iron and core atoms with all other atoms modeled with def2-SVP. The conductor-like screening model[33] (COSMO) was used to simulate a toluene solution ($\varepsilon = 2.4$). Resolution of identity (RI) was used to approximate two electron integrals during geometry optimizations. Initial geometries were obtained from the X-ray crystallographic models. The SCF calculations were tightly converged (TightSCF) with unrestricted spin (UKS). Optimizations were tightly converged (TightOpt). Numerical integrations during all DFT calculations were done on a dense grid (ORCA grid4), and for Mössbauer calculations a very dense grid (ORCA grid7) was used on the iron atoms. The geometry-optimized structures were confirmed to be minima on the potential energy surface by the absence of imaginary frequencies after numerical frequency calculations.

Intrinsic Atomic Orbitals and Intrinsic Bond Orbitals (IAOIBO) analysis was used to analyze the bonding interactions in **2** by orbital localization.[34] The IAOIBO analysis was performed on the broken symmetry (5,5) solution from the crystal structure of **2**. For the purposes of direct comparison with the Mayer Bond Orders in the resting state of FeMoco,[35] the functional TPSSh[36, 37] and the basis set ZORA-def2-TZVP were used for these calculations, and gave very similar results to IAOIBO calculations with the B3LYP functional.

For the XAS DFT calculations, the electronic structure and spectroscopic calculations were performed using the ORCA computational chemistry package version 4.002. Crystallographic coordinates were used for DFT and subsequent TD-DFT calculations of S K-edge and Fe K-edge XAS. Calculations employed the B3LYP functional with the ZORA-def2-SVP basis set. Fe, C, N, and Si atoms were modeled with the ZORA-def2-TZVP basis set. For **2**, the alkylidene atoms were modeled with the def2-TZVP basis set. ZORA for relativistic effects was included in all calculations. Calculations employed broken symmetry (5,5) to account for the antiferromagnetic interaction between high-spin Fe^{III} centers. The coupling constant J was calculated based on the spin Hamiltonian $\hat{H} = -2J\hat{S}_A \cdot \hat{S}_B$,[38, 39] where $J = -(E_{HS} - E_{BS})/(\langle S\rangle^2_{HS} - \langle S\rangle^2_{BS})$.[40, 41] E_{HS} and E_{BS} are the energies of the high symmetry and broken symmetry spin states respectively, and $\langle S\rangle^2_{HS}$ and $\langle S\rangle^2_{BS}$ are the corresponding expectation values for the spin squared operators. The lowest energy solution was used for TD-DFT calculations. S K-edge spectra were plotted using 0.7 eV FWHM broadening and Fe K-edge spectra were plotted using 1 eV FWHM broadening. A shift of 40.34 eV was applied to the calculated S K-edge spectra to correct for the energies.[42] Covalencies were obtained by averaging the percentages of S 3p character in the unoccupied d-orbitals from the broken-symmetry solution.

Discussion of the Electronic Structure of **2** from Computations

Table S3 Comparison of Mössbauer parameters between experiment and computations for various spin states of **2**

**2**	δ_1 (mm s^{-1})	$\|\Delta E_Q\|_1$ (mm s^{-1})	δ_2 (mm s^{-1})	$\|\Delta E_Q\|_2$ (mm s^{-1})	ΔG° (kcal/mol)
Experimental	0.26	1.95	–	–	–
Calculated *S* = 0	0.26	1.65	0.34	2.56	53
Calculated *S* = 2	0.20	0.83	0.40	2.00	16
Calculated *S* = 2 BS	0.21	1.02	0.47	1.32	43
Calculated *S* = 3	0.38	1.54	0.45	1.80	13
Calculated *S* = 3 BS	0.36	1.79	0.45	1.80	11
Calculated *S* = 4	0.44	2.59	0.41	2.42	12
Calculated *S* = 4 BS	0.34	1.90	0.33	1.97	0
Calculated *S* = 5	0.33	2.00	0.35	1.99	14
Calculated *S* = 5 BS	0.35	1.62	0.36	1.67	1

Note: The *S* = 1 solution did not converge, despite multiple efforts.

The calculated BS (4,4) solution, the *S* = 5 solution, and the BS (5,5) solutions were analyzed to give predicted Mössbauer spectra. The isomer shifts came from a linear fit of the electron density at the iron nucleus according to a published correlation derived for other diketiminate-iron complexes.[43] All of these predicted Mössbauer parameters within error of the experimental parameters. The calculated *S* = 5 solution gives a free energy that is too high to be considered the ground state electronic structure solution, and also the *S* = 5 ground state conflicts with the experimentally observed antiferromagnetic coupling from the magnetic measurements (Fig. 4). The calculated BS (4,4) and BS (5,5) solutions both have low free energies, suggesting they both are plausible solutions. The overlap between the lowest-lying corresponding orbitals (d_{x2-y2}) in the calculated BS (4,4) solution is 0.66. This intermediate value is difficult to interpret, though does not indicate electron pairing (typically 0.85 or higher).[44-47] Overall, the simplest description of **2** that has a low energy and has spectroscopic parameters consistent with experiment is that of two antiferromagnetically coupled high spin iron(III) centers that give an *S* = 0 ground state. This assignment is supported by the dc magnetic susceptibility data which show antiferromagnetic coupling between the iron sites, and the low temperature $\chi_M T$ value supports a *S* = 0 ground state, indicating similar electron configurations at each iron subsite.

Though the broken-symmetry (5,5) geometry best aligned with the experimental data of **2**, the predicted Fe–Fe bond distance (2.891 Å) was approximately 0.3 Å longer than the Fe–Fe distance in the crystal structure (2.6027(3) Å). Additionally, the predicted Fe–L bond lengths deviated by an average of 0.038 Å (and up to 0.077 Å) compared to the crystal structure. For this reason, the XAS and IAOIBO computations reported in the text utilized the crystallographic coordinates instead of the optimized geometries to minimize

inaccuracies in the core geometry. Nevertheless, we note that the predicted values from the geometry optimization (calc S p character 5.84%) agree well with experiment (6.17%) and the predicted value from the crystallographic geometry (5.94%).

Discussion of the Electronic Structure of **3** from Computations

The calculated *S* = 2 structure gives the lowest energy for **3**, and the calculated Mössbauer parameters for this state are in good agreement with the experimental values.

Table S4 Comparison of Mössbauer parameters between experiment and computations for various spin states of **3**

**3**	δ (mm s^{-1})	$\lvert\Delta E_Q\rvert$ (mm s^{-1})	ΔG° (kcal/mol)
Experimental	0.43	1.28	–
Calculated *S* = 3	0.31	2.23	15
Calculated *S* = 5	0.37	1.45	0

Note: The *S* = 1 solution did not converge, despite multiple efforts.

Discussion of the Electronic Structure of **4** from Computations

The calculated *S* = 3/2 structure yields the lowest energy for **4**, and the calculated Mössbauer parameters for this state are in good agreement with the experimental values.

Table S5 Comparison of Mössbauer parameters between experiment and computations for various spin states of **4**. RMSD of structure compared to crystal structure.

**4**	δ (mm s^{-1})	$\lvert\Delta E_Q\rvert$ (mm s^{-1})	ΔG° (kcal/mol)	RMSD (Å)
Experimental	0.40	2.23	–	–
Calculated *S* = 1/2	0.40	1.15	18	0.667
Calculated *S* = 3/2	0.49	2.29	0	0.17

IAOIBO Calculations on the Fe–S Bonds in **2** and $[L^{Me}FeS]_2$

Table S6. Mulliken populations for each localized Fe–S orbital with a bond-like localized orbital interaction in the alkylidene sulfide (**2**, left) versus $[L^{Me}FeS]_2$ (right)

Alkylidene Sulfide (**2**)						$[L^{Me}FeS]_2$					
σ	%S	%Fe	SUM	%S	%Fe	σ	S	Fe	SUM	%S	%Fe
103a	0.69	0.29	0.99	70	30	144a	0.68	0.32	1.00	68	32
102b	0.70	0.28	0.97	71	29	141a	0.70	0.29	0.98	71	29
**AVG**	**0.69**	**0.28**		**71**	**29**	143b	0.70	0.29	0.98	71	29
						84b	0.68	0.31	1.00	68	32
						**AVG**	**0.69**	**0.30**		**70**	**30**
π	S	Fe	SUM	%S	%Fe	π	S	Fe	SUM	%S	%Fe
101a	0.82	0.05	0.86	95	5	83a	0.79	0.18	0.97	82	18
100a	0.80	0.16	0.96	84	16	143a	0.81	0.16	0.97	84	16
101b	0.79	0.09	0.88	90	10	142a	0.79	0.15	0.94	84	16
100b	0.80	0.15	0.95	84	16	140a	0.81	0.15	0.97	84	16
**AVG**	**0.80**	**0.11**		**88**	**12**	83b	0.81	0.16	0.97	84	16
						144b	0.79	0.18	0.97	82	18
						142b	0.81	0.15	0.97	84	16
						141b	0.79	0.15	0.94	84	16
						**AVG**	**0.80**	**0.16**		**83**	**17**
**BOTH**				**82**	**18**	**BOTH**				**79**	**21**

Table S7. Individual atom contributions in Fe–S bonds from IAOIBO analysis in alkylidene Sulfide (**2**, left) versus $[L^{Me}FeS]_2$ (right)

Alkylidene Sulfide (**2**)						$[L^{Me}FeS]_2$					
σ	%S	%Fe	SUM	%S	%Fe	σ	S	Fe	SUM	%S	%Fe
103a	0.69	0.32	1.01	68	32	144a	0.67	0.32	0.99	67	33
102b	0.70	0.31	1.00	70	31	141a	0.69	0.29	0.98	70	30
**AVG**				**69**	**31**	143b	0.69	0.29	0.98	70	30
						84b	0.67	0.32	0.99	67	33
						**AVG**				**69**	**31**
π	S	Fe	SUM	%S	%Fe	π	S	Fe	SUM	%S	%Fe
101a	0.82	0.18	0.99	82	18	83a	0.79	0.22	1.01	78	22
100a	0.80	0.19	1.00	81	19	143a	0.81	0.18	1.00	82	18
101b	0.79	0.21	1.00	79	21	142a	0.78	0.21	0.99	79	21
100b	0.80	0.20	1.00	80	20	140a	0.81	0.18	0.99	82	18
**AVG**				**80**	**20**	83b	0.81	0.18	0.99	81	19
						144b	0.79	0.22	1.00	70	30
						142b	0.81	0.18	1.00	82	18
						141b	0.78	0.21	0.99	79	21
						**AVG**	**0.79**	**0.18**		**79**	**21**
**BOTH**				**77**	**23**	**BOTH**				**76**	**24**

The orbital decomposition (Mulliken reduced orbital populations per MO, Table S7) of these IAOIBO localized orbitals provides more accurate values of electron density on each atom in a bond compared to the Mulliken populations output for each localized molecular orbital in the IAOIBO calculations (see Table S6, *vide supra*). This orbital analysis demonstrates that there is no difference in the Fe–S bonding covalency between **2** and $[L^{Me}FeS]_2$, nor is there a difference between the Fe–S covalency in the σ-bonding interactions as well as the π-bonding interactions. This comparable Fe–S covalency between **2** and $[L^{Me}FeS]_2$ is also reflected in the sulfur K-edge XAS spectra. Further, the Mössbauer isomer shifts of both iron(III) sites in **2** (δ = 0.26 mm s^{-1}) and the $[L^{Me}FeS]_2$ analogue (δ = 0.28 mm s^{-1}) reflects the similarity in the σ-donor and the π-acceptor strengths of the ligands.[48]

The comparable Fe–S covalency between these two complexes is especially interesting due to the structural difference observed in the crystal structure, where the Fe–S–Fe angle in the alkylidene sulfide being 16.22(6)° smaller than in $[L^{Me}FeS]_2$ and the average Fe–S bond being 0.116(2) Å shorter. The impact of these structural differences is visible in the localized orbitals with π-bonding character (see Fig. S34, *vide infra*). The IAOIBO analysis shows the implications of the shorter Fe–S bonds and the contracted Fe–S–Fe angle on the Fe–S π-interactions in **2**, where these orbitals are slightly twisted compared to the corresponding interactions in the $[L^{Me}FeS]_2$.

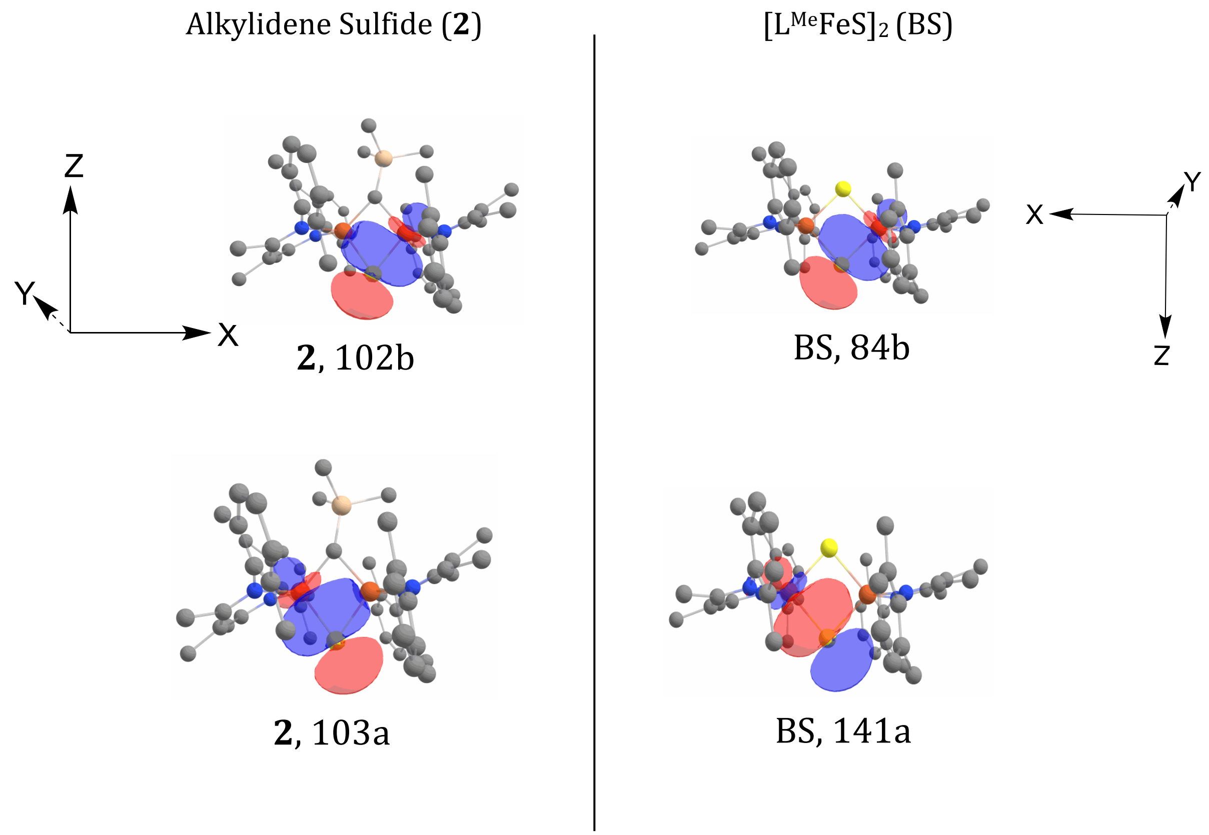

Figure S33. Fe–S σ orbitals from IAOIBO analysis of **2** (left) and $[L^{Me}FeS]_2$ (a = alpha, b = beta)

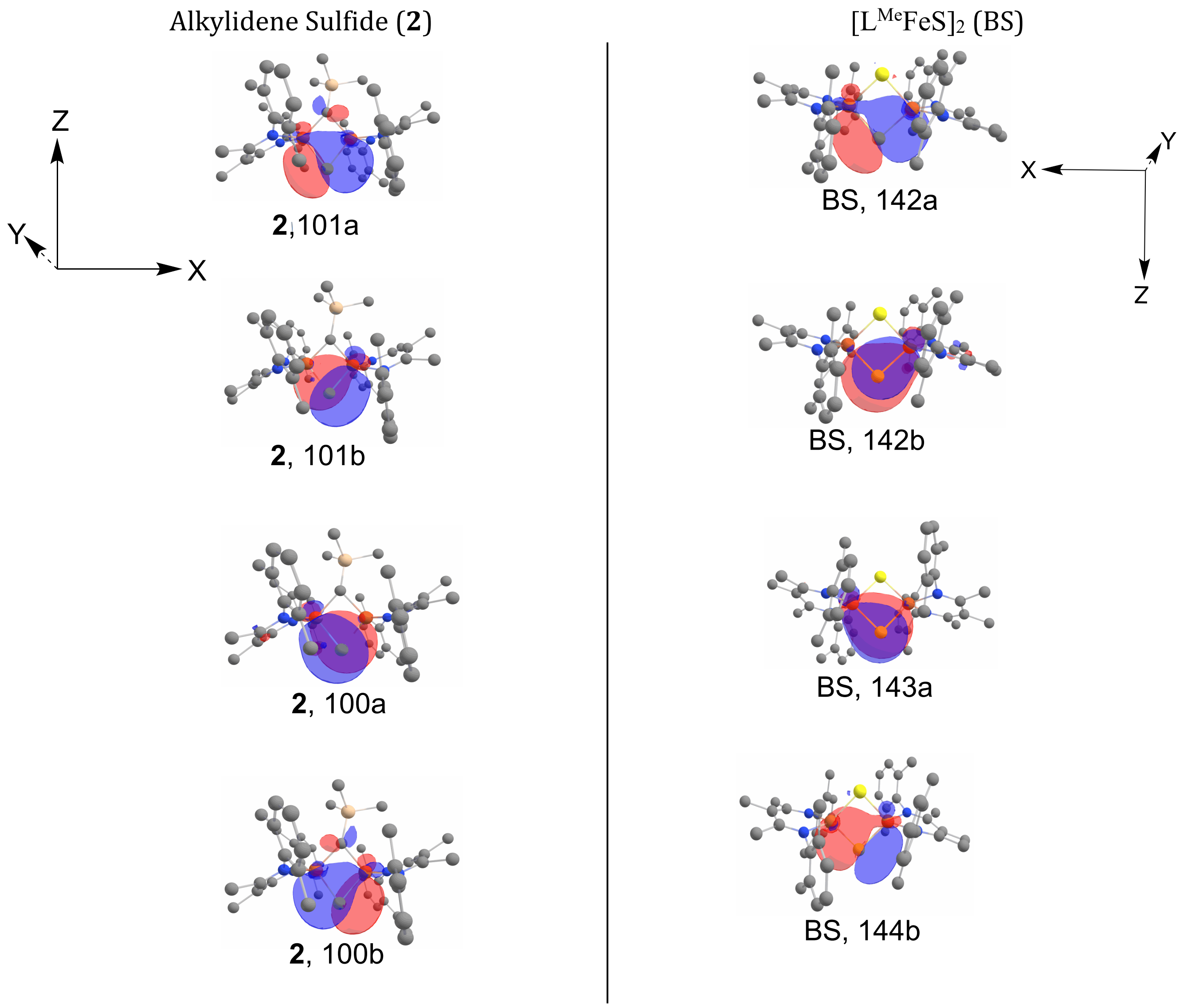

Figure S34. Fe–S π orbitals from IAOIBO analysis (a = alpha, b = beta) of the alkylidene Sulfide (**2**, left) and $[L^{Me}FeS]_2$ (right)

IAOIBO Calculations on the Fe–C Bonds in **1** and **2**

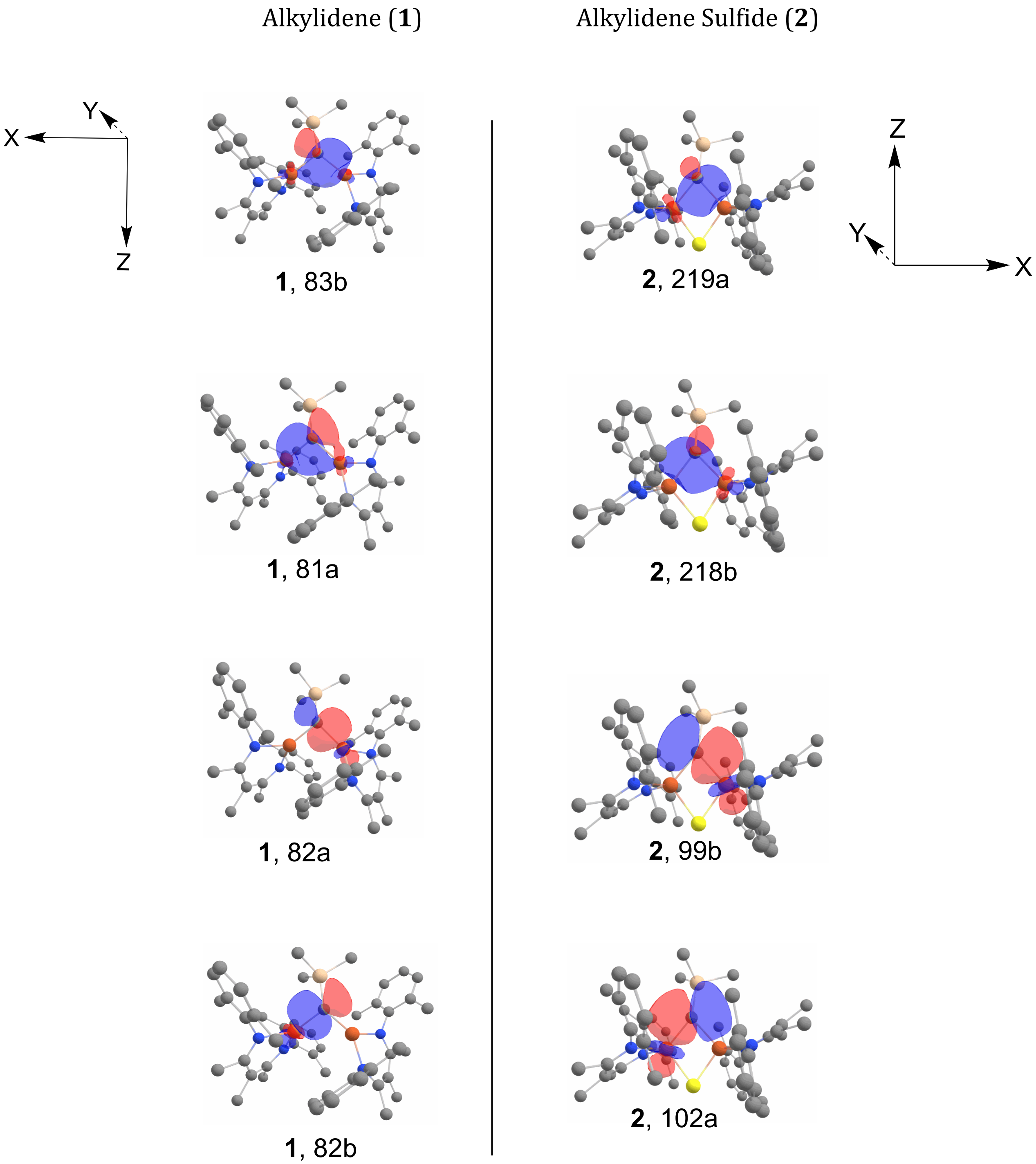

Figure S35. Fe–C σ orbitals from IAOIBO analysis in the alkylidene (**1**, left) and the alkylidene sulfide (**2**, right) (a = alpha, b = beta)

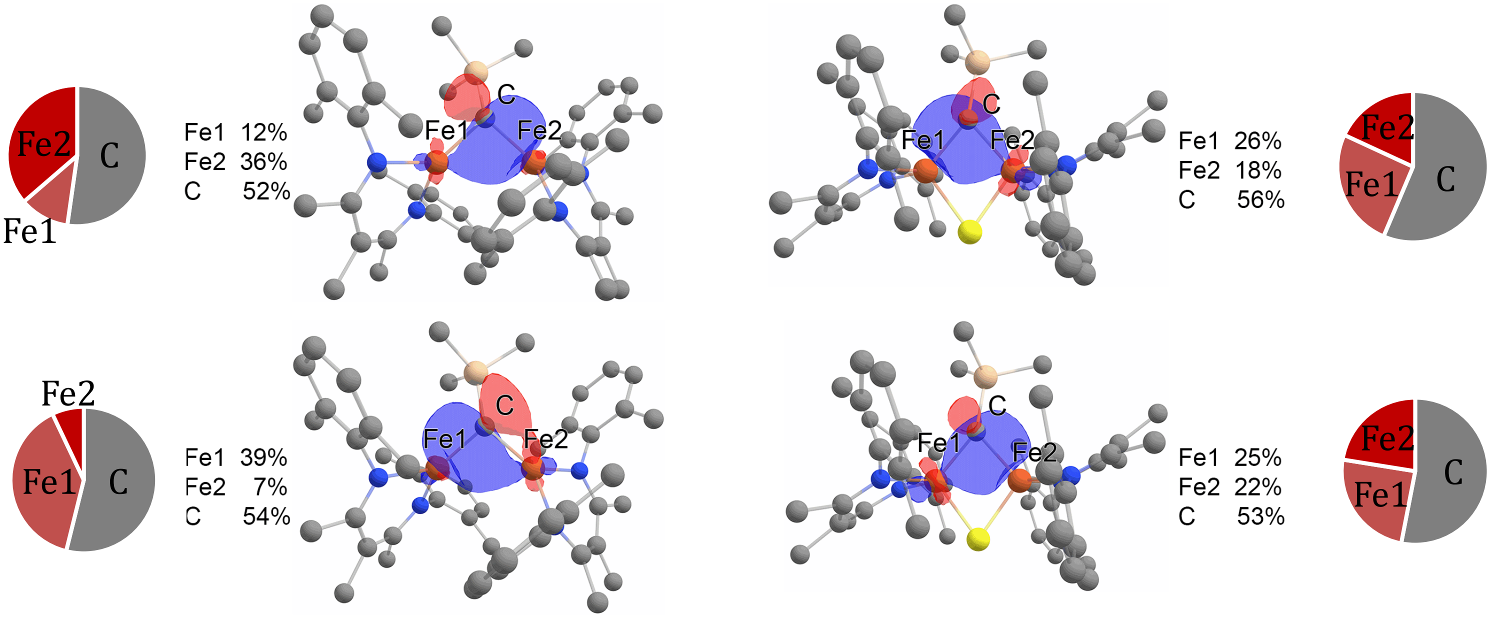

1 **2**

Figure S36. Isosurface plot of select Fe–C interactions in **1** (left) and **2** (right) with the total spin-density for Fe and the alkylidene carbon atom listed for each plot.

Sample Input Files

Example Orca Input for Geometry Optimization

```
! UKS B3LYP TightOpt ZORA-def2-SVP ZORA Grid4 NoFinalGrid RIJCOSX SARC/J
! TightSCF D3BJ CPCMC(toluene)

%basis newgto Fe "ZORA-def2-TZVP" end
 newgto N "ZORA-def2-TZVP" end
 newgto Si "ZORA-def2-TZVP" end
 newgto S "ZORA-def2-TZVP" end
end

%pal nprocs 20 end

%rel OneCenter true
end

%output
Print [ P_Basis ] 2
Print [ P_MOs ] 1
end

%scf MaxIter 1600
 shift shift 0.3 erroff 0.0 end
 end

* xyz 0 11
COORDINATES
*
```

Example Orca Input for Broken Symmetry Calculations

```
! UKS B3LYP ZORA-def2-SVP ZORA UNO UCO Grid4 NoFinalGrid RIJCOSX
SARC/J
! TightSCF D3BJ CPCMC(toluene)

%basis newgto Fe "ZORA-def2-TZVP" end
 newgto N "ZORA-def2-TZVP" end
 newgto Si "ZORA-def2-TZVP" end
 newgto S "ZORA-def2-TZVP" end
end

%pal nprocs 20 end

%rel OneCenter true
end

%output
```

```
Print [ P_Basis ] 2
Print [ P_MOs ] 1
end

%scf MaxIter 800
 shift shift 0.3 erroff 0.0 end
 BrokenSym 5,5
end

* xyz 0 1
COORDINATES
*
```

Example Orca Input for Mossbauer Calculations

```
! UKS B3LYP ZORA-def2-TZVP ZORA Grid4 NoFinalGrid RIJCOSX SARC/J NoRI
! TightSCF D3BJ CPCMC(toluene) MOread
%moinp "file.gbw"

%Method SpecialGridAtoms 26
 SpecialGridIntAcc 7 end

%pal nprocs 20 end

%scf MaxIter 800
 shift shift 0.3 erroff 0.0 end
 end

* xyz 0 11
COORDINATES
*
```

Example Orca Input for IAOIBO Calculations

```
! UKS B3LYP ZORA-def2-TZVP ZORA Grid4 GridX5 RIJCOSX SARC/J
! TightSCF SlowConv MOread
%moinp "#.gbw"

%loc
locMet IAOIBO
end

* xyzfile 0 1 #.xyz
```

Example Orca Input for XAS Calculations

```
!B3LYP RIJCOSX ZORA-def2-SVP ZORA SARC/J D3BJ CPCMC(toluene) UKS
!NormalPrint VeryTightSCF SlowConv Grid4 NoFinalGrid

%pal nprocs 2 end

%basis newgto Fe "ZORA-def2-TZVP" end
          newgto N "ZORA-def2-TZVP" end
          newgto Si "ZORA-def2-TZVP" end
          newgto S "ZORA-def2-TZVP" end
          end

%method SpecialGridAtoms 26
       SpecialGridIntAcc 7
       end

%MaxCore 4000

%SCF   Directresetfreq 1
   DIIS MaxEq 15
        end
    Shift Shift 0.5
         Erroff 0.1
         end
    MaxIter 1500
          BrokenSym 5,5
   end

%tddft   NRoots 50
        MaxDim 500
        OrbWin[0] = 2, 2, -1, -1
        OrbWin[1] = 2, 2, -1, -1
        DoQuad true
        end

* xyz 0 11
```
COORDINATES

XAS Spectra

Data Collection All data were measured at the Stanford Synchrotron Radiation Lightsource (SSRL) under ring conditions of 3.0 GeV and 500 mA. Samples were prepared in an inert-atmosphere glovebox and were measured as solids. For Fe K-edge measurements, samples were ground with BN to a final concentration of 5 weight % Fe, pressed into 1 mm aluminum spacers and sealed with 37 μm Kapton tape. For S K-edge measurements, samples were prepared by grinding to a fine powder and spreading thinly onto 38 μm low-S Mylar tape.

Fe K-edge measurements were carried out at SSRL Beamline 9-3, which is equipped with a 16-pole, 2-Tesla wiggler source. Incident X-ray radiation was monochromated using a double Si(220) crystal monochromator. Samples were maintained at 10 K in a liquid He cryostat during data collection. Spectra were collected in fluorescence mode, with X-rays detected by a passivated implanted planar silicon (PIPS) detector placed at a 90° angle to the sample. Inelastic scatter was attenuated using a Soller slits fitted with a Ni filter. A Fe foil and a third ionization chamber upstream of the sample were used for internal energy calibration. Four scans were measured and averaged for each compound.

S K-edge measurements were carried out at SSRL Beamline 4-3, which is equipped with a 20-pole, 2-Tesla wiggler source. All samples were measured in a He atmosphere at room temperature in fluorescence mode using a Lytle detector. Intensity was normalized with respect to the incident beam using a He-filled ion chamber upstream of the sample. Data were collected from 2400 to 2800 eV. Four scans were measured and averaged for each compound.

Data Processing. Raw data were averaged and normalized using SIXPACK[49] and processed using IGOR 6.37. Sulfur K-edge energies were calibrated to the lowest energy peak in the S K-edge XAS spectrum of potassium thiosulfate at 2472.0 eV. Spectra were then averaged and the background was removed by applying a linear normalization to the pre-edge region below 2450 eV. The edge-jump was normalized to unit intensity by applying a quadratic normalization to the post-edge region above 2490 eV. Pre-edge peak positions were obtained from plots of the second derivatives (Figure S37). Normalized spectra were fit using a Monte-Carlo based, nonlinear least squares fitting algorithm[50] (Figures S38-39). An initial set of 300 fits was obtained using floating parameters and allowing for 8 peaks in the region of 2450 eV to 2600 eV and a FWHM of 5 eV. The edge-jump was constrained to a region between 2478 eV and 2490 eV. A histogram of these initial fits was used to determine bounds set for another 50 fits, from which the fit with the lowest R^2 was used to obtain pre-edge areas. Only peaks below 2472 eV were included in the pre-edge area.

Fe K-edge XAS energies were calibrated by setting the inflection point of an internal iron foil standard to 7111.2 eV. Spectra were then averaged and the background was removed by applying a linear normalization to the pre-edge region below 7063 eV. The post-edge region was normalized to unit intensity by applying a quadratic normalization to the post-edge region above 7130 eV. Pre-edge peak positions were obtained from plots of the second derivatives (Figure S40).

Calculation of percent S 3p

The pre-edge area of the S K-edge was used to calculate the percentage S 3p character in the unoccupied metal d orbitals by equation 1.[51]

$$D_o(S\ 1s \rightarrow \psi^*) = \alpha^2 h I_s / 3n \qquad (1)$$

where D_o is the pre-edge area, α^2 reflects the S 3p character, h is the number of holes, I_s is the radial dipole integral, and n is the number of absorbers. A linear correlation between I_s and the energy of the 1s to 4p transition reported by Solomon was used to determine the value of I_s for these compounds.[52] The 1s to 4p transition was assigned to the large peak at 2476.9 eV, giving an I_s value of 14.7.

Table S8. Analysis of the S K-edge XAS data.

	Pre-edge area	% S p (experimental)	% S p (calculated)
**[L^{Me}FeS]$_2$**	3.52 ± 0.25	14.39	11.94
2	3.01 ± 0.18	6.17	5.94

Table S9. Analysis of the Fe K-edge XAS data.

	Pre-edge (eV)	Edge (eV)
1	7111.4, 7111.9	7115.0
**[L^{Me}FeS]$_2$**	7111.4, 7112.6	7118.0
2	7111.4, 7112.9	7117.8

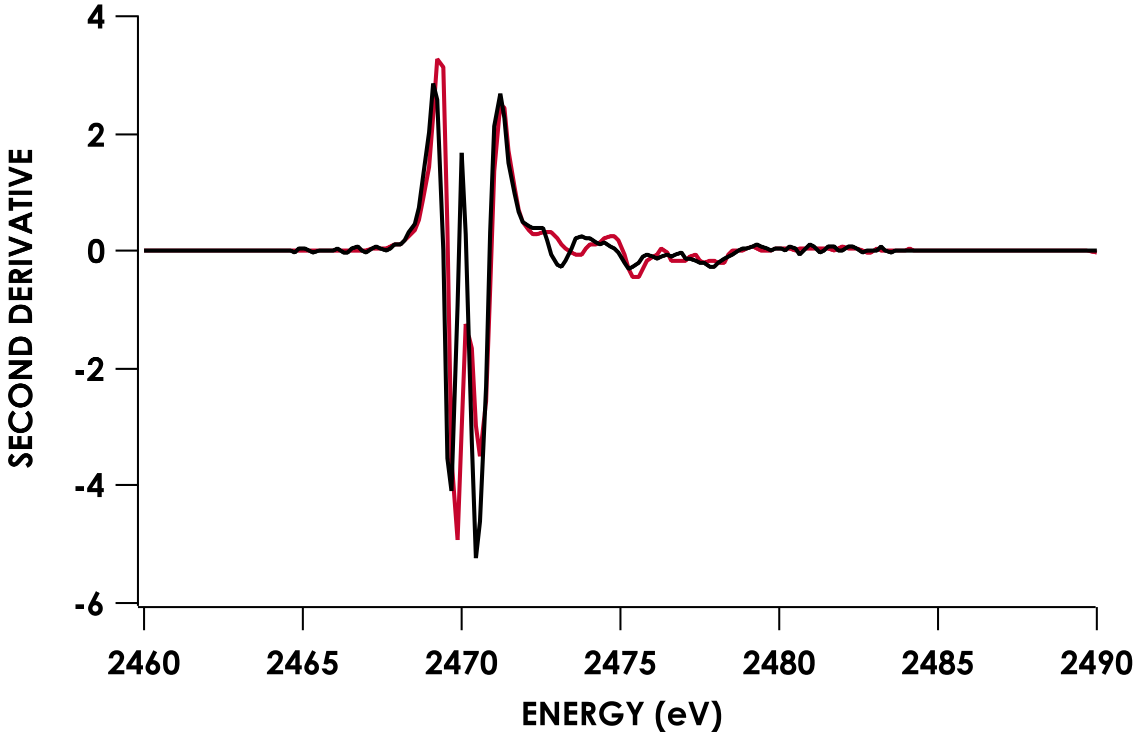

Figure S37. Second derivates of the S K-edge XAS spectra of **2** (black) and $[L^{Me}FeS]_2$ (red).

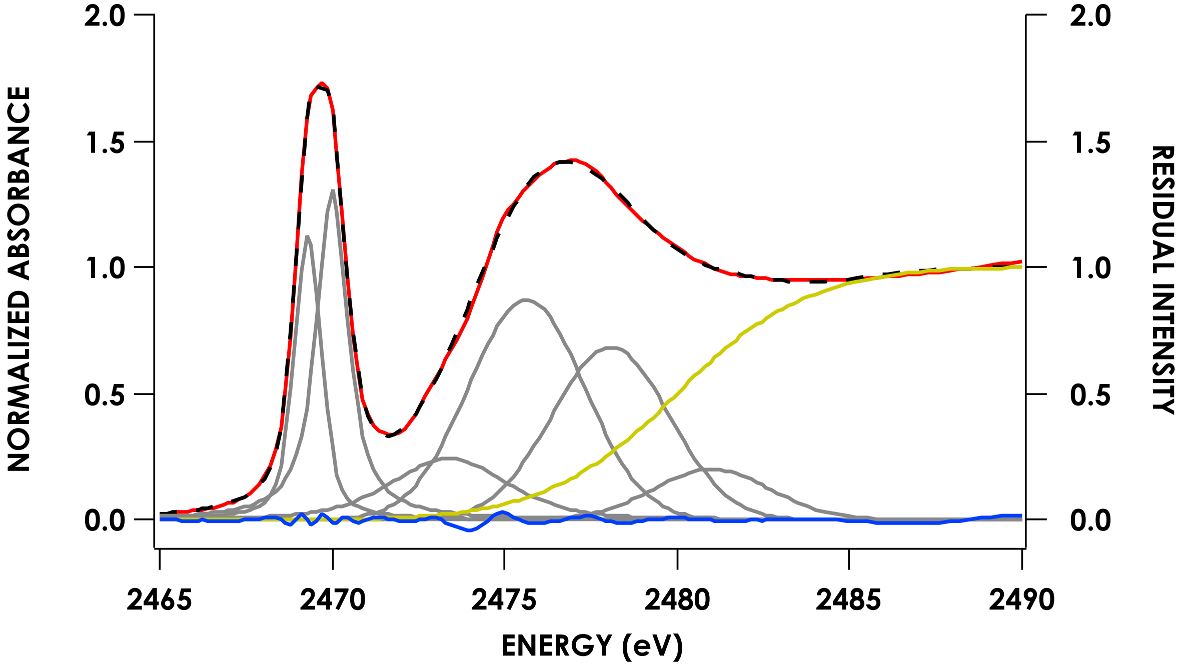

Figure S38. Final fit of the S K-edge XAS spectrum of $[L^{Me}FeS]_2$. The experimental spectrum is shown in red along with the overall fit (dashed black), individual peaks (grey), edge jump (yellow) and residual (blue).

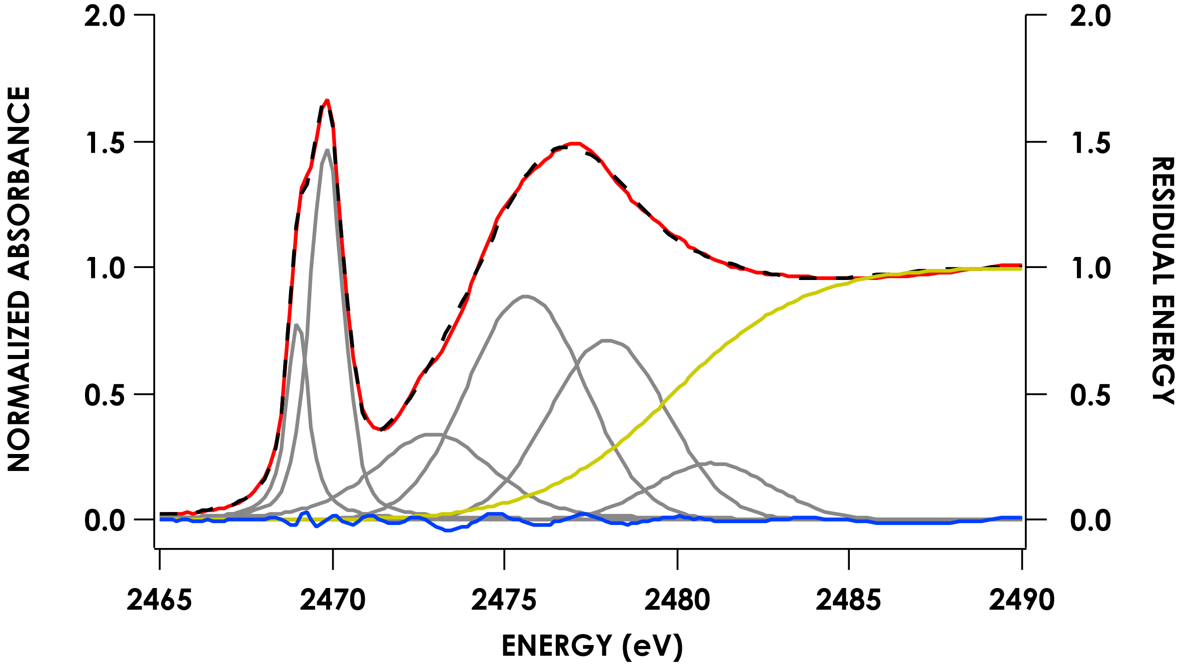

Figure S39. Final fit S K-edge XAS spectrum of **2**. The experimental spectrum is shown in red along with the overall fit (dashed black), individual peaks (grey), edge jump (yellow) and residual (blue).

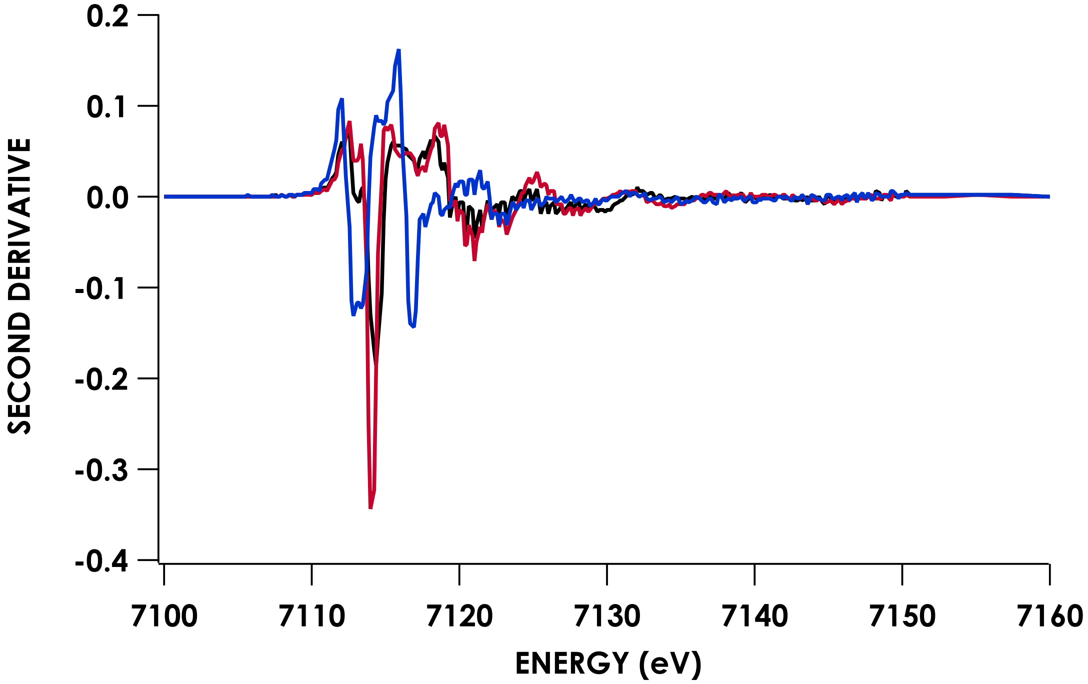

Figure S40. Second derivates of the Fe K-edge XAS spectra of **1** (blue), **2** (black), and $[L^{Me}FeS]_2$ (red).

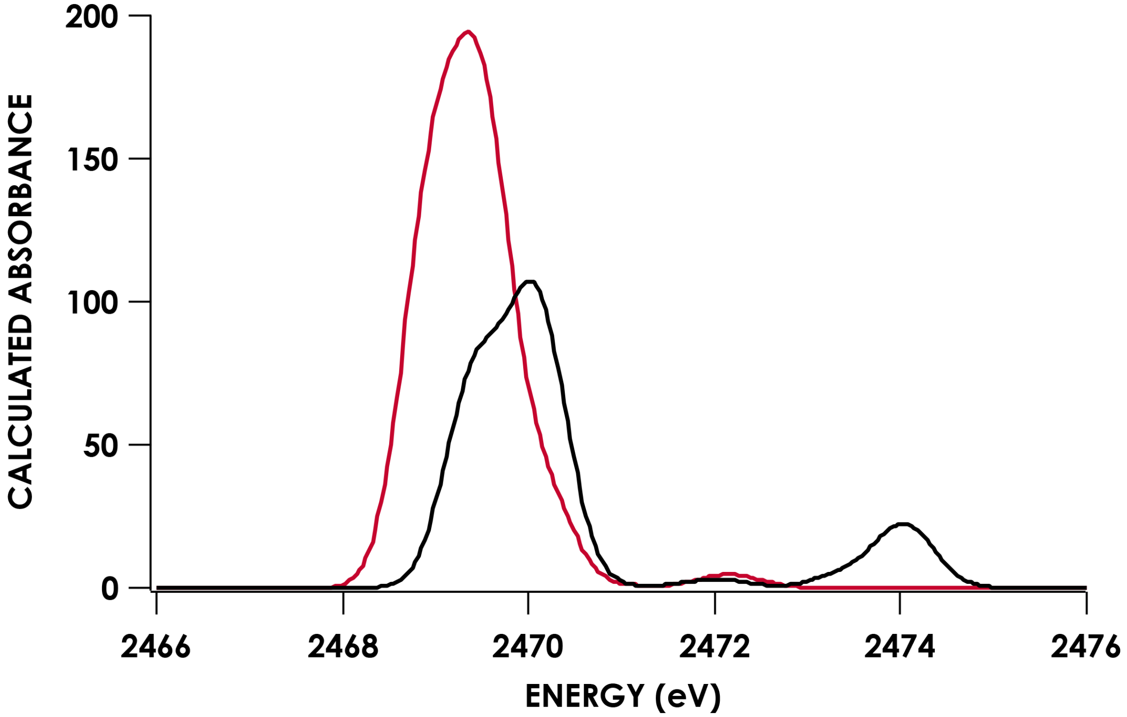

Figure S41. Calculated sulfur K-edge XAS spectra of **2** (black) and $[L^{Me}FeS]_2$ (red).

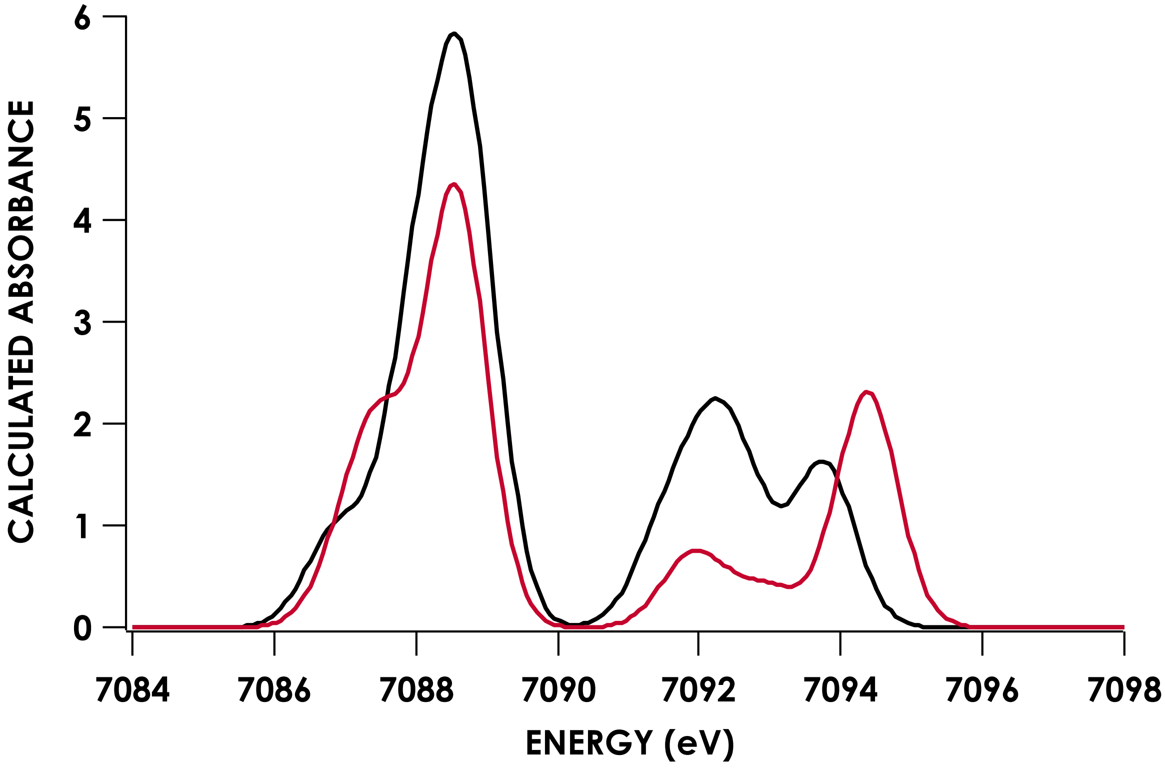

Figure S42. Calculated Fe K-edge XAS spectra of **2** (black) and $[L^{Me}FeS]_2$ (red).

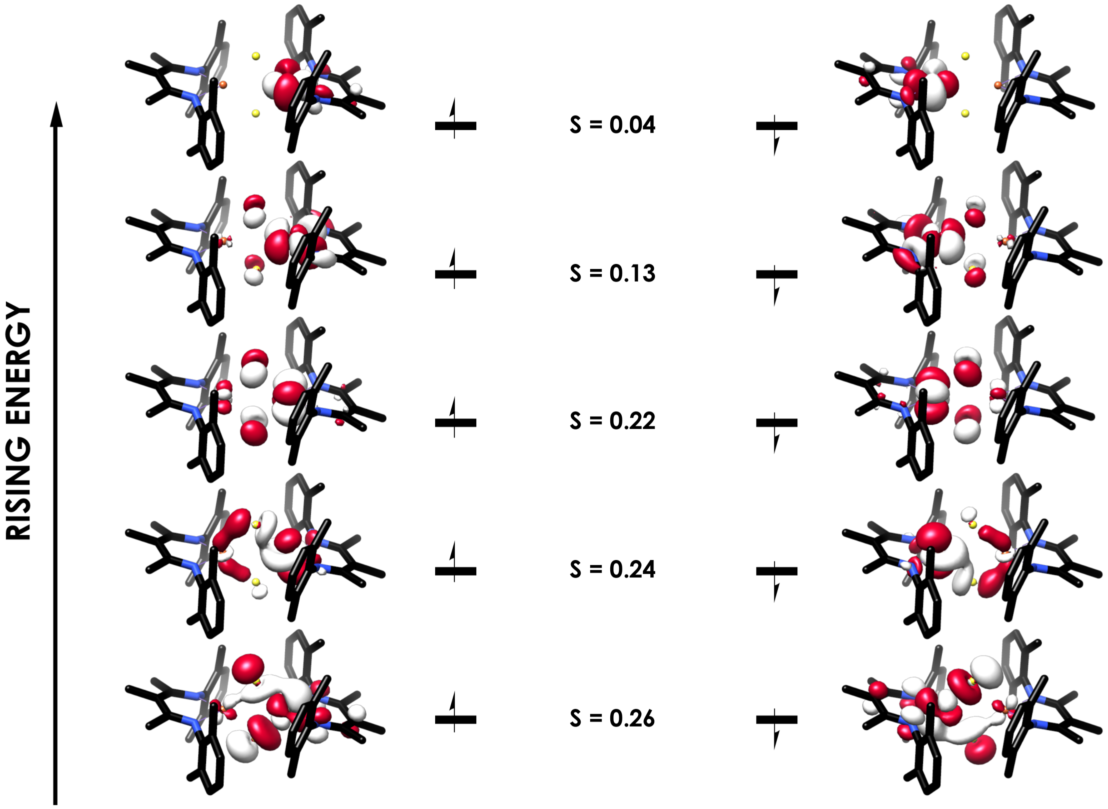

Figure S43. Truncated molecular orbital diagram of **2** generated from the broken symmetry UKS solution. UCOs are shown with the alpha orbitals on the left and the beta orbitals on the right. Orbitals are plotted at an isovalue of 0.03 au. S represents the overlap between the alpha and beta orbitals.

Crystallographic Studies

Low-temperature diffraction data (ω-scans) were collected on a Rigaku MicroMax-007HF diffractometer coupled to a Dectris Pilatus3R detector with Mo Kα (λ = 0.71073 Å) for the structure of **2** and **4**. Similar data were collected for **3** on a Rigaku MicroMax-007HF diffractometer coupled to a Saturn994+ CCD detector with Cu Kα (λ = 1.54178 Å). The diffraction images were processed and scaled using Rigaku Oxford Diffraction software (CrysAlisPro; Rigaku OD: The Woodlands, TX, 2015). The structure was solved with SHELXT and was refined against F^2 on all data by full-matrix least squares with SHELXL (Sheldrick, G. M. Acta Cryst. 2008, A64, 112–122). All non-hydrogen atoms were refined anisotropically. Hydrogen atoms were included in the model at geometrically calculated positions and refined using a riding model. The isotropic displacement parameters of all hydrogen atoms were fixed to 1.2 times the U value of the atoms to which they are linked (1.5 times for methyl groups). The full numbering scheme of compounds **2**, **3**, and **4** can be found in the full details of the X-ray structure determination (CIF), which is included as Supporting Information. Structures have been deposited with the CCDC, with deposition numbers 2009741 (**2**), 2009739 (**3**), and 2009740 (**4**). These data can be obtained free of charge from The Cambridge Crystallographic Data Center via www.ccdc.cam.ac.uk/data_request/cif.

Table S10. Details of Crystal Structures

Compound	2	3	4
Data code	007c-20026	007b-17080	007c-19063
CCDC Number	2009741	2009739	2009740
Empirical formula	C50.50 H70 Fe2 N4 S Si	C26 H38 Fe N2 Si	C44 H74.63 Fe K N2 O7.50 Si
Temperature (K)	93(2)	93(2) K	93(2)
Wavelength	0.71073 Å	1.54184 Å	0.71073 Å
Crystal system	Monoclinic	Monoclinic	Triclinic
Space group	$P2_1/c$	$P2_1/m$	P-1
a (Å)	15.2840(3)	a = 7.46458(13) Å	12.7212(4)
b (Å)	14.0745(3)	b = 17.4351(3) Å	13.5703(4)
c (Å)	22.2861(4)	c = 10.11223(13) Å	14.5849(5)
α (°)	90	90	73.777(2)
β (°)	94.457(2)	99.0427(14)	85.650(3)
γ (°)	90	90	86.169(2)
V ($Å^3$)	4779.57(16)	1299.70(3) Å3	2407.84(14)
Z	4	2	2
ρ (Mg/m^3)	1.258	1.182	1.206
μ (mm^{-1})	0.713	5.182	0.472
Completeness	99.80%	99.90%	99.80%
Data / restraints / parameters	11850 / 51 / 573	2375 / 0 / 183	9163 / 139 / 583
$R1$, w$R2$ (I > 2σ(I))	0.0307, 0.0785	0.0303, 0.0850	0.0419, 0.1003
$R1$, $wR2$ (all data)	0.0449, 0.0870	0.0320, 0.0864	0.0481, 0.1033
GOF	1.092	1.063	1.034
Largest Diff. Peak, Hole ($e/Å^3$)	0.456, -0.380	0.240, -0.278	0.658, -0.653

Refinement and Model Details for 2

All non-hydrogen atoms were refined anisotropically. Hydrogen atoms were included in the model at geometrically calculated positions and refined using a riding model. The isotropic displacement parameters of all hydrogen atoms were fixed to 1.2 times the U value of the atoms to which they are linked (1.5 times for methyl groups). The only exception is H1 which was found in the difference map and freely refined. The pentane crystallized near the inversion center. The best model was obtained by suppressing the special position constraints and placing a whole model pentane (see Guzei, I. A. (2014). J. Appl. Crystallogr. 47, 806-809). Several reflections were improperly recorded and omitted from the refinement.

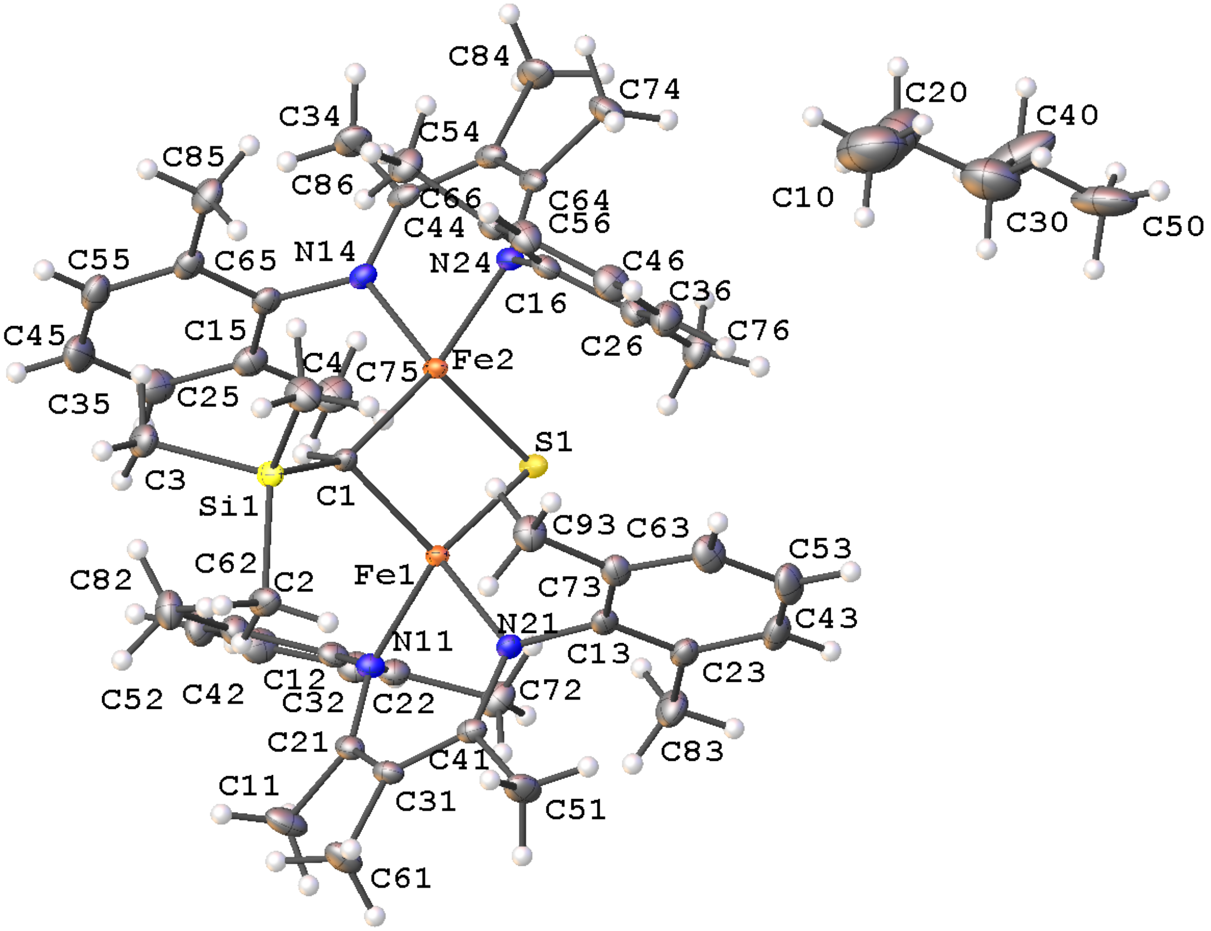

Figure S44. The complete numbering scheme of **2** with 50% thermal ellipsoid probability levels. The hydrogen atoms are shown as circles for clarity.

Refinement and Model Details for 3

The iron complex sits near the crystallographic mirror plane, where disordered modeling of atoms Fe1, C41, C14, C24, C34, C44, and Si14. All atoms were constrained to 0.5 site occupancy factors, and the special position constraints were suppressed. The only exception to this condition was atom H34, which was coincident with the crystallographic mirror plane.

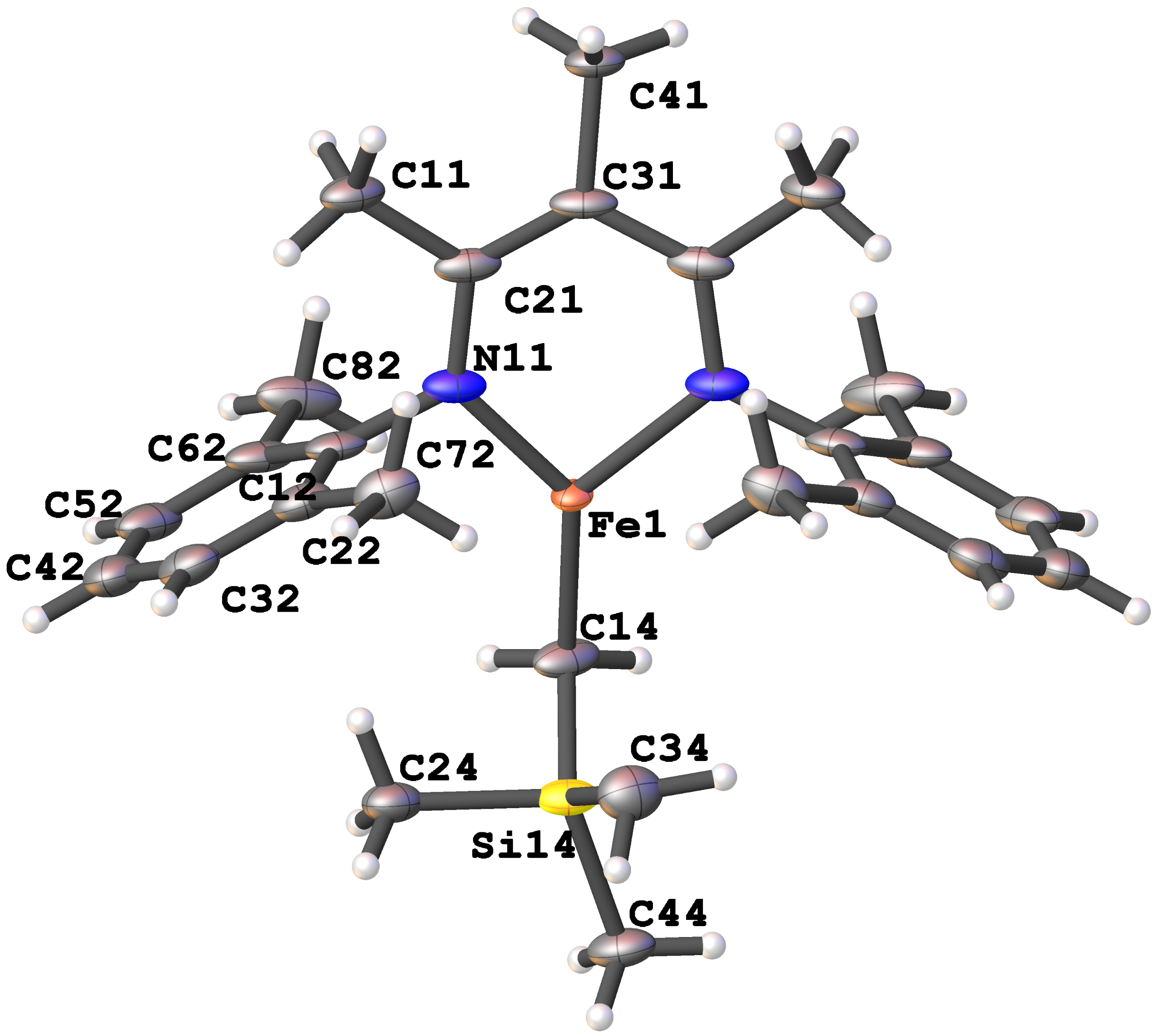

Figure S45. The complete numbering scheme of **3** with 50% thermal ellipsoid probability levels. The hydrogen atoms are shown as circles for clarity.

Refinement and Model Details for 4

The coordinating solvents on the 18-crown-6 are all disordered. The THF with atom labels that contain the suffix "C" are discorded across the crystallographic inversion center. The model is constrained to have ideal geometries; the thermal parameters are restrained to behave as a rigid group with similar values. The site occupancy was constrained to a value of 0.5. There is a mix of ether and THF at the other axial position of the 18-crown-6. These positions were found in the difference map. The thermal parameters in the model are restrained to behave as a rigid group with similar values. The site occupancy were freely refined o values of 0.69/0.31. All hydrogen atoms in the disordered model were geometrically placed in expected locations.

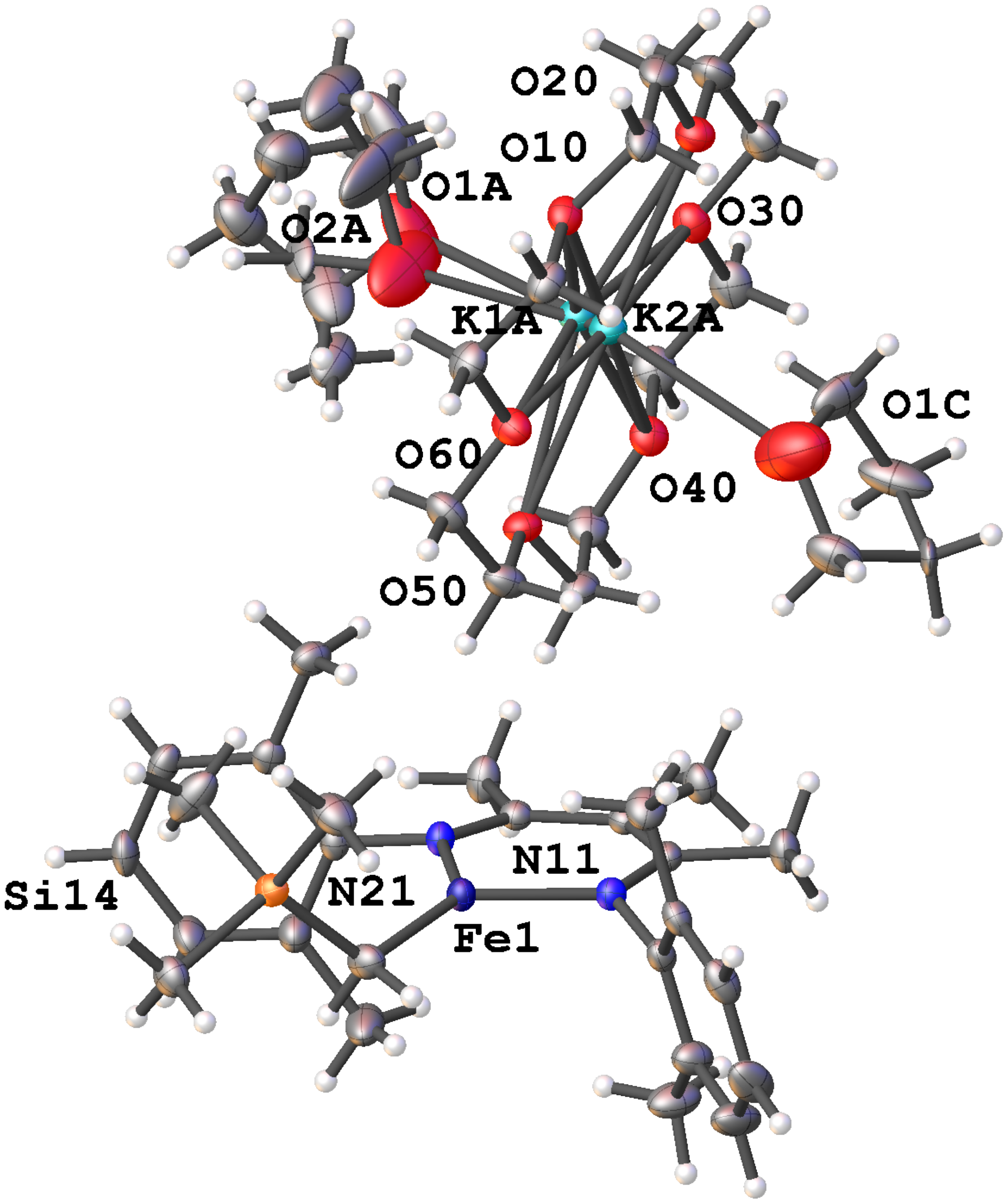

Figure S46. The complete numbering scheme of **4** with 50% thermal ellipsoid probability levels. The hydrogen atoms are shown as circles for clarity.

Discussion of τ_4 Parameter in **1** and **2**

The structural parameter analysis of the alkylidene carbons in **1** and **2** was performed using the crystal structures. In these structures, the alkylidene proton can be found in the difference map, but this is tangential since the largest angles (Fe–C–Si angles) are independent of the H atom.[53, 54]

Table S11 Structural Parameter Analysis of the Alkylidene Carbon in **1** and **2**

	α	β	τ_4	τ_4'
**1**	111.7	128.1	0.85	0.80
**2**	127.2	141.3	0.65	0.61

References

1. M. M. Rodriguez, E. Bill, W. W. Brennessel and P. L. Holland, *Science*, 2011, **334**, 780.
2. D. E. DeRosha, N. A. Arnet, B. Q. Mercado and P. L. Holland, *Inorg. Chem.*, 2019, **58**, 8829-8834.
3. R. J. Kern, *J. Inorg. Nucl. Chem.*, 1962, **24**, 1105-1109.
4. M. Brookhart, B. Grant and A. F. Volpe, *Organometallics*, 1992, **11**, 3920-3922.
5. R. García-Rodríguez and H. Liu, *J. Am. Chem. Soc.*, 2012, **134**, 1400-1403.
6. I. M. Smallwood, in *Handbook of Organic Solvent Properties*, ed. I. M. Smallwood, Butterworth-Heinemann, Oxford, 1996, DOI: https://doi.org/10.1016/B978-0-08-052378-1.50003-7, p. vii.
7. E. M. Schubert, *J. Chem. Educ.*, 1992, **69**, 62.
8. S. Stoll and A. Schweiger, *J. Magn. Reson.*, 2006, **178**, 42-55.
9. M. E. Reesbeck, K. Grubel, D. Kim, W. W. Brennessel, B. Q. Mercado and P. L. Holland, *Inorg. Chem.*, 2017, **56**, 1019-1022.
10. S. F. McWilliams, D. L. J. Broere, C. J. V. Halliday, S. M. Bhutto, B. Q. Mercado and P. L. Holland, *Nature*, in press.
11. S. F. McWilliams, E. Bill, G. Lukat-Rodgers, K. R. Rodgers, B. Q. Mercado and P. L. Holland, *J. Am. Chem. Soc.*, 2018, **140**, 8586-8598.
12. J. B. Geri, J. P. Shanahan and N. K. Szymczak, *J. Am. Chem. Soc.*, 2017, **139**, 5952-5956.
13. K. C. MacLeod, S. F. McWilliams, B. Q. Mercado and P. L. Holland, *Chem. Sci.*, 2016, **7**, 5736-5746.
14. M. W. Weatherburn, *Anal. Chem.*, 1967, **39**, 971-974.
15. R. Battino, T. R. Rettich and T. Tominaga, *J. Phys. Chem. Ref. Data*, 1984, **13**, 563-600.
16. D. L. M. Suess, C. Tsay and J. C. Peters, *J. Am. Chem. Soc.*, 2012, **134**, 14158-14164.
17. G. Ung and J. C. Peters, *Angew. Chem. Int. Ed.*, 2015, **54**, 532-535.
18. G. A. Bain and J. F. Berry, *J. Chem. Educ.*, 2008, **85**, 532.
19. N. F. Chilton, R. P. Anderson, L. D. Turner, A. Soncini and K. S. Murray, *J. Comput. Chem.*, 2013, **34**, 1164-1175.
20. R. T. Azuah, L. R. Kneller, Y. Qiu, P. L. W. Tregenna-Piggott, C. M. Brown, J. R. D. Copley and R. M. Dimeo, *J. Res. Natl. Inst. Stand. Technol.*, 2009, **114**, 341-358.
21. J. Krzystek, A. Ozarowski and J. Telser, *Coord. Chem. Rev.*, 2006, **250**, 2308-2324.
22. F. Neese, *WIREs Comp. Mol. Sci.*, 2012, **2**, 73-78.
23. F. Neese, *WIREs Comp. Mol. Sci.*, 2018, **8**, e1327.
24. A. D. Becke, *J. Chem. Phys.*, 1993, **98**, 5648-5652.
25. C. Lee, W. Yang and R. G. Parr, *Phys. Rev. B*, 1988, **37**, 785-789.
26. S. H. Vosko, L. Wilk and M. Nusair, *Can. J. Phys.*, 1980, **58**, 1200-1211.
27. F. Weigend and R. Ahlrichs, *Phys. Chem. Chem. Phys.*, 2005, **7**, 3297-3305.
28. P. J. Stephens, F. J. Devlin, C. F. Chabalowski and M. J. Frisch, *J. Phys. Chem.*, 1994, **98**, 11623-11627.

29. S. Grimme, J. Antony, S. Ehrlich and H. Krieg, *J. Chem. Phys.*, 2010, **132**, 154104.
30. S. Grimme, S. Ehrlich and L. Goerigk, *J. Comput. Chem.*, 2011, **32**, 1456-1465.
31. C. van Wüllen, *J. Chem. Phys.*, 1998, **109**, 392-399.
32. E. van Lenthe, A. van der Avoird and P. E. S. Wormer, *J. Chem. Phys.*, 1998, **108**, 4783-4796.
33. A. Klamt and G. Schüürmann, *J. Chem. Soc., Perkin 2*, 1993, 799-805.
34. G. Knizia, *J. Chem. Theor. Comp.*, 2013, **9**, 4834-4843.
35. B. Benediktsson and R. Bjornsson, *submitted*, 2020.
36. J. P. Perdew, S. Kurth, A. Zupan and P. Blaha, *Phys. Rev. Lett.*, 1999, **82**, 2544-2547.
37. J. P. Perdew, J. Tao, V. N. Staroverov and G. E. Scuseria, *J. Chem. Phys.*, 2004, **120**, 6898-6911.
38. L. Noodleman and E. R. Davidson, *J. Chem. Phys* 1986, **109**, 131-143.
39. L. Noodleman, *J. Chem. Phys.*, 1981, **74**, 5737-5743.
40. K. Yamaguchi, Y. Takahara, T. Fueno, in: V.H. Smith (Ed.), Applied Quantum Chemistry, V. Reidel, Dordrecht, 1986, p. 155.
41. T. Soda, Y. Kitagawa, T. Onishi, Y. Takano, Y. Shigeta, H. Nagao, Y. Yoshioka and K. Yamaguchi, *Chem. Phys. Lett.*, 2000, **319**, 223-230.
42. S. DeBeer George and F. Neese, *Inorg. Chem.*, 2010, **49**, 1849-1853.
43. S. F. McWilliams, E. Brennan-Wydra, K. C. MacLeod and P. L. Holland, *ACS Omega*, 2017, **2**, 2594-2606.
44. S. Zhang, Q. Wang, L. M. Thierer, A. B. Weberg, M. R. Gau, P. J. Carroll and N. C. Tomson, *Inorg. Chem.*, 2019, **58**, 12234-12244.
45. S. Ye, C.-Y. Geng, S. Shaik and F. Neese, *Phys. Chem. Chem. Phys.*, 2013, **15**, 8017-8030.
46. W. A. Goddard, T. H. Dunning, W. J. Hunt and P. J. Hay, *Acc. Chem. Res.*, 1973, **6**, 368-376.
47. F. Neese, *J. Phys. Chem. Solids*, 2004, **65**, 781-785.
48. P. Gütlich, E. Bill and A. X. Trautwein, *Mössbauer spectroscopy and transition metal chemistry: fundamentals and applications*, Springer Science & Business Media, 2010.
49. Webb, S. https://www.sams-xrays.com/sixpack.
50. R. C. Walroth, K. C. Miles, J. T. Lukens, S. N. MacMillan, S. S. Stahl and K. M. Lancaster, *J. Am. Chem. Soc.*, 2017, **139**, 13507-13517.
51. F. Neese, B. Hedman, K. O. Hodgson and E. I. Solomon, *Inorg. Chem.*, 1999, **38**, 4854-4860.
52. R. Sarangi, S. DeBeer George, D. J. Rudd, R. K. Szilagyi, X. Ribas, C. Rovira, M. Almeida, K. O. Hodgson, B. Hedman and E. I. Solomon, *J. Am. Chem. Soc.*, 2007, **129**, 2316-2326.
53. L. Yang, D. R. Powell and R. P. Houser, *Dalton Trans.*, 2007, 955-964.
54. A. Okuniewski, D. Rosiak, J. Chojnacki and B. Becker, *Polyhedron*, 2015, **90**, 47-57.
